# The Aryl Hydrocarbon Receptor Pathway: A Key Component of the microRNA-Mediated AML Signalisome

**DOI:** 10.3390/ijerph9051939

**Published:** 2012-05-18

**Authors:** Julia E. Rager, Rebecca C. Fry

**Affiliations:** 1 Department of Environmental Sciences and Engineering, Gillings School of Global Public Health, The University of North Carolina, 135 Dauer Drive, CB 7431, UNC, Chapel Hill, NC 27599, USA; Email: jrager@live.unc.edu; 2 Curriculum in Toxicology, The University of North Carolina, 135 Dauer Drive, CB 7431, UNC, Chapel Hill, NC 27599, USA

**Keywords:** aryl hydrocarbon receptor, gene expression, leukemia, microRNA, systems biology

## Abstract

Recent research has spotlighted the role of microRNAs (miRNAs) as critical epigenetic regulators of hematopoietic stem cell differentiation and leukemia development. Despite the recent advances in knowledge surrounding epigenetics and leukemia, the mechanisms underlying miRNAs’ influence on leukemia development have yet to be clearly elucidated. Our aim was to identify high ranking biological pathways altered at the gene expression level and under epigenetic control. Specifically, we set out to test the hypothesis that miRNAs dysregulated in acute myeloid leukemia (AML) converge on a common pathway that can influence signaling related to hematopoiesis and leukemia development. We identified genes altered in AML patients that are under common regulation of seven key miRNAs. By mapping these genes to a global interaction network, we identified the “AML Signalisome”. The AML Signalisome comprises 53 AML-associated molecules, and is enriched for proteins that play a role in the aryl hydrocarbon receptor (AhR) pathway, a major regulator of hematopoiesis. Furthermore, we show biological enrichment for hematopoiesis-related proteins within the AML Signalisome. These findings provide important insight into miRNA-regulated pathways in leukemia, and may help to prioritize targets for disease prevention and treatment.

## 1. Introduction

In the past decade, microRNAs (miRNAs) have emerged as major players in cellular regulation, especially related to carcinogenesis. The expression profiles of miRNAs can be used to classify tumor types and tumor differentiation state with high levels of accuracy [1]. Hematological malignancies, in particular, are considered to be largely influenced by miRNAs. For example, miRNAs are known to regulate hematopoietic stem and/or progenitor cell differentiation [2]. Bone marrow cells from leukemia patients also show significantly altered miRNA expression profiles, where miRNAs related to hematopoiesis are some of the most consistently dysregulated miRNAs in leukemia [3,4]. Because of their role in leukemogenesis, miRNAs are also considered promising therapeutic targets for new leukemia treatment strategies [4].

### 1.1. miRNA Background

The first miRNA was discovered in *Caenorhabditis elegans* in 1993 [5]. In 2001 miRNAs received a high level of interest, when they were first reported to act as gene silencers [6,7]. Now seen as important epigenetic regulators, miRNAs play a key regulatory role in mRNA abundance and protein production [8]. By base pairing to target mRNAs, miRNAs can cause mRNA degradation and/or translational repression [9]. Current estimates suggest that mammalian miRNAs regulate more than 60% of all protein-coding genes [10]. Because miRNAs play such pivotal roles in gene regulation, it is important to understand the relationship of miRNAs with disease.

### 1.2. Acute Myeloid Leukemia (AML) Background

Research involving epigenetics has recently focused on the study of cancers with high prevalence and/or mortality rates. For example, leukemia is prevalent in the United States, where it is estimated that 1 in 75 people will be diagnosed during their lifetime [11]. Acute myeloid leukemia (AML) is one of the most common types of leukemia among adults [11]. AML is characterized as a clonal disorder of hematopoietic progenitor cells which have lost the ability to differentiate normally and respond to regulators of proliferation or pro-apoptotic signals [12]. These changes result in the accumulation of progenitor cells arrested at various stages of development, which can eventually lead to fatal infection, bleeding, or organ infiltration [12].

### 1.3. Pathways Modulated in Leukemia

The mechanisms underlying leukemogenesis often involve changes in cellular signaling, which can inhibit hematopoietic progenitor cells from: (i) responding to normal signals regulating proliferation, and/or (ii) differentiating into mature red blood cells, monocytes, neutrophils, and platelets [12]. Many pathways have been shown to be altered in AML blood and bone marrow cells, including the nuclear factor kappa-B (NFκB) [13], mitogen-activated protein kinase (MAPK) [14], and Wnt/β-catenin pathways [15]. The aryl hydrocarbon receptor (AhR) pathway is also implicated in leukemogenesis [16], where, for example, primary human T-cell leukemia cells have shown up-regulated AhR expression and activation [17]. Furthermore, dysregulated AhR signaling within hematopoietic stem cells has been proposed as a possible mechanism linking benzene exposure to AML development [18].

### 1.4. Previous Studies on miRNAs in AML

Since the initial miRNA tumor classification studies [1], many other researchers have investigated miRNAs and their involvement in AML. For example, numerous studies have correlated miRNA expression levels to cytogenetics in AML patients with the goal of identifying diagnostic and prognostic indicators, as reviewed by Marcucci *et al*. [3]. Some studies have also correlated the expression levels of individual miRNAs with genome-wide mRNA expression levels, and identified subsets of genes likely regulated by individual miRNAs in AML. In cytogenetically normal AML patients, for example, miR-181a expression levels have been shown to inversely correlate with the expression of genes involved in innate immunity [19].

### 1.5. Study Aim

We hypothesize that miRNAs likely act in a concert to exert their biological functions. More specifically, we propose that the coordinated action of miRNAs on their transcriptional targets may influence key biological pathways involved in leukemogenesis. A recent paper [20] in which the role of multiple miRNAs on transcriptional regulation has been shown supports this hypothesis. What remains to be identified is whether a key biological pathway may be the target of such concerted miRNA control. Our study addresses this issue using a novel analytical strategy to compare epigenetically-regulated signaling within AML cells *versus* non-leukemia cells.

While previous studies have identified associations between individual miRNAs and leukemia-related gene expression profiles, we employed a systems-level approach to identify converging pathways regulated by several key AML-associated miRNAs. We predicted that the statistical integration of existing databases, along with systems-level pathway analyses, would reveal high ranking biological pathways altered at both the gene expression and epigenetic (e.g., miRNA) level in AML patients. Thus, this study’s goal was to elucidate novel biological relationships that may underlie leukemogenesis.

## 2. Methods

### 2.1. Identifying the AML-Associated Gene Expression Signature

In order to identify a genomic signature associated with AML, we performed a statistical analysis comparing gene expression levels between AML patients and non-leukemic individuals. Specifically, a publically available database containing microarray data from patients with AML (n = 202), myelodysplastic syndrome (n = 164), or from non-leukemic, control patients (n = 69) was downloaded from the National Center for Biotechnology Information’s Gene Expression Omnibus [21]. This dataset was generated in an investigation by Mills *et al*., where microarray-based classifiers were identified to predict the risk of AML transformation in patients with myelodysplastic syndrome [22]. Of the 202 AML patients, 21 were categorized with complex aberrant karyotype, while 181 were categorized with normal karyotype or other abnormalities. This differential karyotype was included in the subsequent analysis. To note, all samples were obtained from untreated patients at the time of diagnosis, as part of the collaborative Microarray Innovations in Leukemia (MILE) study [22,23].

Total RNA from bone marrow mononuclear cells was extracted and hybridized to microarrays, as described previously [22,24]. Gene expression data derived from probe set intensity signals from 202 AML and 69 non-leukemic patients were used. To identify genes that were differentially expressed in AML patients, statistical analyses were performed as follows. Data were filtered for removal of background (<abs [100]) in 20% of the samples. Differential expression was defined as a significant difference in transcript levels between AML *versus* non-leukemic patients, where three statistical requirements were set: (i) a fold change of ≥2.5 or ≤−2.5 (AML *versus* non-leukemic); (ii) *p* value <0.01 (ANOVA); and (iii) a false discovery rate corrected q-value <0.01. Analysis of variance (ANOVA) *p* values were calculated using Partek^®^ Genomics Suite^TM^ software, version 6.5 [25]. To control the rate of false positives, q-values were calculated as the minimum “positive false discovery rate” that can occur when identifying significant hypotheses [26]. This criteria was set based on our published methods [27,28], where statistical stringency is employed to allow the identification of genes significantly associated with disease status, while allowing minimal false positives. The genes that met these statistical requirements were identified as significantly differentially expressed in AML patients, and represent the AML-associated gene expression signature.

### 2.2. Identifying AML-Associated miRNAs and Their Transcriptional Targets

A set of miRNAs modulated in AML patients was identified through an extensive literature review. The miRNAs were selected based on their altered expression levels in AML *versus* normal cells and their relevance to leukemia development. The studies included were Bousquet *et al*. [29], Cammarata *et al*. [30], Garzon *et al*. [31], Han *et al*. [32], O’Connell *et al*. [33], and Wang *et al*. [34].

In order to understand the effects that the AML-associated miRNAs (n = 7) may cause at the gene expression level, computational predictions of the transcriptional targets of the AML-associated miRNAs were carried out. Here, TargetScanHuman [35] algorithms were employed to identify potential matches between the 3’-untranslated mRNA regions and miRNA seed sequences for each of the miRNAs [36]. The resulting predicted miRNA-mRNA interactions were filtered for the probability of preferentially conserved targeting (P_CT_) ≥0.5. This P_CT_ filter controlled for background conservation across mammals by accounting for mutational biases, dinucleotide conservation rates, and individual untranslated region conservation rates [10].

### 2.3. Network, Pathway, and Functional Enrichment Analysis

Network analysis was performed to identify biological pathways that are targets for miRNA-mediated control in AML. For this analysis, a list of AML-associated genes predicted to be targeted by at least one of the AML-associated miRNAs was overlaid onto a global interaction network. Networks were algorithmically constructed based on connectivity, as enabled through Ingenuity Pathway Analysis [37]. Canonical pathways within the constructed networks were then identified. Over-represented pathways were defined as pathways that contain more AML-associated genes than expected by random chance, as calculated using the right-tailed Fisher’s Exact Test. Pathways with enrichment *p* values <0.05 were considered significant. In a similar fashion, functional enrichment analysis was performed to identify biological functions and disease signatures significantly associated with the AML-associated gene signature predicted to be regulated by AML-associated miRNAs. Significance was calculated in the same manner as the pathway enrichment analysis, where functions with enrichment *p* values <0.0001 were considered significant.

## 3. Results

### 3.1. Identifying the AML Signalisome

**Figure 1:**
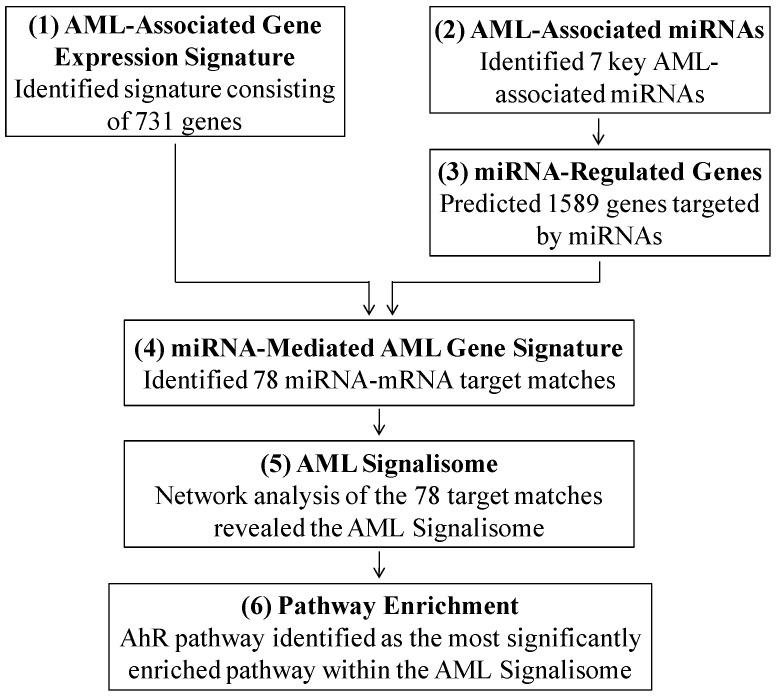
Steps in identifying the AML Signalisome.

In this study, we predicted that miRNAs act together to control genomic signaling of biological pathways associated with AML. To test this hypothesis, we analyzed genomic data from individuals with or without AML, and integrated the genomic data with miRNAs and their transcriptional targets in a six step process (Figure 1). Through this process we uncovered for the first time an AML network, or Signalisome, that is regulated by multiple miRNAs.

#### 3.1.1. AML-Associated Gene Expression Signature Is Identified

**Figure 2:**
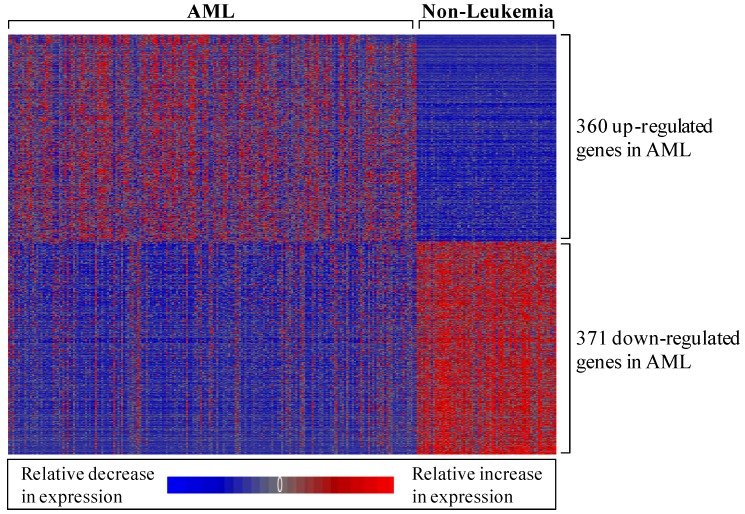
Heat map displaying the relative expression levels of the 731 AML-associated genes. Expression levels are z-score normalized.

To identify a set of genes differentially expressed in AML patients relative to non-leukemic patients, we performed a statistical analysis using publically available data. Data from Mills *et al*. [22] were used in a new manner to compare the genome-wide expression levels between 202 AML patients and 69 non-leukemic patients. Statistical analysis revealed 731 genes (represented by 1,119 probe sets) that were significantly differentially expressed in bone marrow mononuclear cell samples from AML *versus* non-leukemic patients (Figure 2; see electronic supplementary information, Table S1). As the samples were collected at the time of diagnosis for patients enrolling in the MILE study, this genomic signature represents a baseline state and is not confounded by treatment. This gene list was used as the AML-associated gene expression signature throughout the remaining analyses. Interestingly, several of the AML-associated genes overlapped with those highlighted in the Mills *et al*. report [22] and are involved in AML progression, including fms-related tyrosine kinase 3 (*FLT3*), v-kit Hardy-Zuckerman 4 feline sarcoma viral oncogene homolog (*KIT*), runt-related transcription factor 1 (*RUNX1*), and Wilms tumor 1 (*WT1*).

A comparison between the AML-associated gene signature and a previously published set of AML-related genes was also performed. The gene set to which we compared the AML-associated gene expression signature consisted of 66 genes that have been identified as accurate predictors of overall survival for AML patients [38]. Given the difference in study designs, the degree of overlap between the gene signatures is robust. Specifically, a total of 24 of the 66 genes (36%) identified as predictors of survival in AML patients were also present in our AML-associated gene signature (see electronic supplementary information, Table S2).

#### 3.1.2. AML-Associated miRNAs Are Identified

Based on previous studies that have highlighted the changes in sets of miRNAs, we hypothesized that miRNAs likely act in a coordinated manner to mediate their regulatory effects through key biological pathways. Thus to identify miRNAs known to be altered in expression in AML patients, a thorough literature review was conducted. A total of seven AML-associated miRNAs were identified, consisting of miRNAs with significant differential expression in blood and/or bone marrow samples collected from AML patients in comparison to non-leukemic patients. To detail, miR-125b, miR-126, miR-142-3p, miR-155, miR-223, miR-29a, and miR-29b have all shown altered expression levels in AML samples *versus* non-leukemic samples [29,30,31,32,33,34] (Table 1). Furthermore, all of these miRNAs are suggested to play roles in leukemogenesis [32,33,39,40,41,42,43,44].

**Table 1:** AML-associated miRNA database.

miRNA	Reference(s) for Differential Expression in AML Patients	Reference(s) for Suggested Role in Leukemogenesis
miR-125b	[29]	[39]
miR-126	[30,31]	[40]
miR-142-3p	[34]	[41]
miR-155	[31,33]	[33]
miR-223	[30]	[42,44]
miR-29a	[30,32,34]	[32]
miR-29b	[31]	[43]

#### 3.1.3. miRNA-Regulated Genes Are Predicted

To understand genomic changes that occur in AML patients likely via changes in miRNA expression, we computationally predicted transcriptional targets of the seven AML-associated miRNAs. Using seed match-based algorithms, 196 genes were predicted to be targeted by miR-125b, 22 by miR-126, 308 by miR-142-3p, 164 by miR-155, 68 by miR-223, 805 by miR-29a, and 179 by miR-29b. In total, 1,589 unique genes were predicted to be targeted by at least one of the seven AML-associated miRNAs (see electronic supplementary information, Table S3).

#### 3.1.4. The miRNA-Mediated AML Gene Signature Is Identified

With the goal of identifying genes with altered expression levels in AML that are likely regulated by miRNAs, we compared the 731 genes of the AML-associated gene expression signature (identified in step 1 of the analysis) to the 1,589 genes predicted to be targeted by AML-associated miRNAs (identified in step 3 of the analysis). This comparison showed that 78 genes contained in the AML-associated gene signature are likely regulated by AML-associated miRNAs (see electronic supplementary information, Table S4).

#### 3.1.5. The AML Signalisome Is Identified

**Figure 3:**
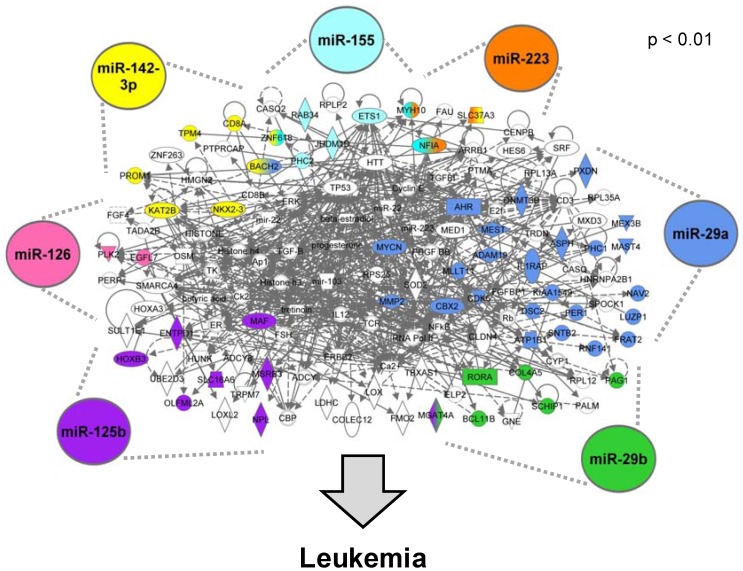
The AML Signalisome. Proteins encoded by genes dysregulated in AML patients are shaded in colors corresponding to their regulatory miRNA(s). White molecules are associated with the AML-associated genes.

In order to identify whether the miRNA-mediated genomic changes occur in the context of high level interactions, molecular networks were constructed using the 78 AML-associated genes that were predicted to be regulated by the AML-associated miRNAs. A total of five significant (*p* value < 0.01) sub-networks were constructed (see electronic supplementary information, Table S5). Interestingly, four of the five sub-networks interact, which when combined, form the AML Signalisome (Figure 3). This AML Signalisome consists of 133 proteins, 53 of which are encoded by genes altered in AML and predicted to be targeted by AML-associated miRNAs.

#### 3.1.6. Members of the AhR Pathway Are Enriched within the AML Signalisome

**Figure 4:**
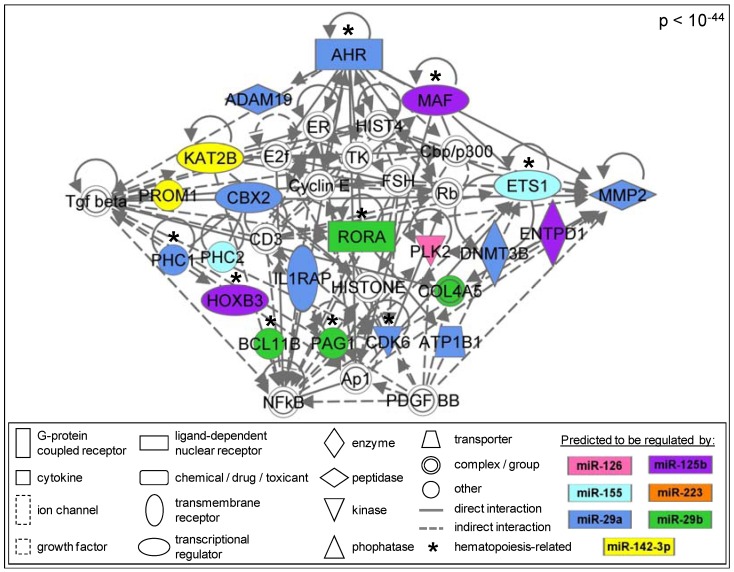
AhR-related signaling molecules are enriched within the AML Signalisome.

Our study set out to identify high ranking biological pathways altered at the gene expression level and likely regulated by miRNAs in AML patients. Through pathway enrichment analysis, we identified six canonical pathways that are significantly enriched within the AML Signalisome (Table 2). Biologically, these six pathways represent well-characterized metabolic and cell signaling pathways that are enriched among proteins encoded by AML-associated genes likely regulated by miRNAs. The most significant pathway within the AML Signalisome is the aryl hydrocarbon receptor (AhR) signaling pathway (*p* = 0.017). AhR pathway signaling is also present in the most significant (*p* < 10^−44^) sub-network within the AML Signalisome (Figure 4). In addition, we analyzed genes identified as AML-associated after exclusion of gene expression obtained from patients with an aberrant karyotype (n = 21). Indeed, the identification of the enrichment of genes that play a role in the AhR pathway was independent of the exclusion of aberrant karyotype samples (data not shown).

To further establish the biological relevance of our findings, we performed a biological function and disease signature enrichment analysis. Here, 18 functions or disease signatures were identified as significantly associated with the AML Signalisome (see electronic supplementary information, Table S6). Of note, the four most significant functions or disease signatures were cellular development (*p* = 1 × 10^−6^), cancer (*p* = 3 × 10^−6^), hematological system development and function (*p* = 3 × 10^−6^), and hematopoiesis (*p* = 3 × 10^−6^).

**Table 2:** Canonical pathways enriched within the AML Signalisome.

Pathway	*p* value
Aryl Hydrocarbon Receptor Signaling	0.017
Hepatic Fibrosis/Hepatic Stellate Cell Activation	0.019
Airway Pathology in Chronic Obstructive Pulmonary Disease	0.032
Calcium Signaling	0.032
Aminosugars Metabolism	0.032
HER-2 Signaling in Breast Cancer	0.038

## 4. Discussion

In this study, we set out to identify key biological pathways that are under concerted miRNA-mediated regulation in AML patients. Specifically, we hypothesized that miRNAs likely act together to control pathway signaling related to hematopoiesis and leukemia development. Previous studies have evaluated the influence of individual miRNAs on gene expression signatures present in AML cells [43,45,46]. However, there is a paucity of data regarding AML-associated genomic signatures and altered pathways that are regulated through the combined effects of multiple miRNAs. Our study aimed to fill this knowledge gap by performing statistical analyses on genomic databases, predicting miRNA-mRNA interactions, and constructing molecular networks likely regulated by multiple miRNAs in AML patients.

The first step in our analysis was to identify an AML-associated gene expression signature. Here, publicly available data containing gene expression microarray data from 271 patients’ bone marrow mononuclear cell samples [22] were statistically assessed. Of the 202 AML patients used in this study, 90% of them were described as having normal karyotype. Using stringent statistical parameters, we identified an AML-associated gene signature consisting of 731 genes altered in AML patients in comparison to non-leukemic individuals. Not surprisingly, many of the genes within the AML-associated gene signature have known involvement in AML disease progression. For example, several of the AML-associated genes have been recognized previously for their involvement in AML progression, including *FLT3*, *KIT*, *RUNX1*, and *WT1* [22]. In addition, a gene set of 66 genes has been identified as an accurate predictor of overall survival in AML patients [38]. Of these 66 genes, 24 (36%) are present in our AML-associated gene signature.

In order to understand epigenetic regulation in leukemia, we next identified miRNAs that are dysregulated in AML patients and predicted their transcriptional targets. Here, miRNAs were selected if they have shown significant differential expression in AML *versus* non-leukemic blood or bone marrow samples. In addition, the AML-associated miRNAs were required to have suggested roles in leukemogenesis. With these criteria, seven miRNAs were selected as AML-associated miRNAs: miR-125b, miR-126, miR-142-3p, miR-155, miR-223, miR-29a, and miR-29b. Seed match-based algorithms were then employed to predict 1589 genes regulated by the miRNAs. Of the 1,589 predicted genes, 78 were also within the AML-associated gene signature. Therefore, we find that 78 of the 731 genes (10.7%) with altered expression levels in AML patients are likely targets of the seven key AML-associated miRNAs.

The percentage of genes likely regulated by AML-associated genes may seem low, as mammalian miRNAs are estimated to regulate more than 60% of all protein-coding genes [10]. Furthermore, ectopic transfection of miR-29a and miR-29b has been associated with the altered expression levels of 572 and 480 genes, respectively, in myeloid leukemia cells [43]. However, these analyses were highly focused and limited to the role of seven miRNAs in the transcriptional response. Thus, the finding is in line with the analytical strategy we employed.

We set out to determine whether the miRNA-regulated AML-associated genes interact and uncovered the AML Signalisome. This AML Signalisome was identified based on molecular interactions between proteins encoded by the 78 genes within the miRNA-mediated AML gene signature. The resulting AML Signalisome includes network interactions of molecules whose transcription are altered in AML cells. The AML Signalisome clearly illustrates that molecular signaling altered in AML cells is likely influenced by the coordinated actions of multiple miRNAs acting on common pathways. This finding provides novel evidence supporting our prediction that miRNAs likely act together to regulate important pathways altered in AML cells.

Within the AML Signalisome, there is a significant enrichment for genes involved in AhR signaling, as well as functional enrichment for cellular development, cancer, hematological system development/function, and hematopoiesis. In particular, all seven AML-associated miRNAs, miR-125b, miR-126, miR-142-3p, miR-155, miR-223, miR-29a, and miR-29b, were predicted to regulate the most significant sub-network within the AML Signalisome containing AhR pathway signaling. Interestingly, the AhR pathway is known to play a major role in the regulation of hematopoiesis, where altered AhR signaling can contribute to hematological disorders, including leukemia [16]. Although the exact mechanisms by which AhR regulates hematopoietic stem cell function or number are unknown, some plausible mechanisms have been proposed [16]. One of the proposed mechanisms involves altered AhR levels and/or activation, leading to altered expression of AhR-regulated genes. The resulting transcriptional dysregulation may cause blocked differentiation of hematopoietic stem cells and the accumulation of progenitor stem cells [16]. Alternative mechanisms linking altered AhR signaling to leukemogenesis may also exist [16]. 

Altered AhR signaling, potentially regulated via AML-associated miRNAs, is of high significance and may represent a key mechanism potentially underlying leukemogenesis. It is important to note that the AhR pathway is a key regulator of stress and xenobiotic response [47]. As such, the AhR pathway could be influenced by chemotherapeutic treatment. In our study, the dysregulation of the AhR pathway is clearly disease-associated, as the genomic profiles used to study systems-level interactions were abstracted from AML samples collected at time of diagnosis and not post-treatment. Interestingly, a study on another epigenetic regulator, cytosine DNA methylation, showed that the *AHR* promoter region is frequently hypermethylated in acute lymphoblastic leukemia [48]. To our knowledge, this is the first study to identify the AhR pathway as potentially dysregulated and under the control of multiple miRNAs in leukemia patients.

Other pathways besides AhR signaling have been identified as altered through miRNA regulation in AML. For example, miR-29b has been shown to influence signaling related to NFκB, Sp1 transcription factor (SP1), and histone deacetylase (HDAC) [45]. In addition, the nuclear factor [erythroid-derived 2]-like 2 (NRF2), tumor necrosis factor-α (TNF), and heme oxygenase-1 (HO-1) have all been shown to be influenced by miRNAs in AML cells, and have been suggested to play a role in AML chemo-resistance [4]. Our results do not highlight these previously identified interactions for several potential reasons. For instance, we require the influence of multiple miRNAs instead of just a single miRNA, unlike most other related investigations. In addition, our AML-associated gene expression signature was identified through a comparison of AML patients and non-leukemic patients. Most other studies compare only within AML subtypes. Lastly, studies investigating miRNA regulation in AML are currently lacking systems biology-based analyses, making it difficult to compare our results to previous research on pathway modifications. Future research should focus on molecular pathways altered at the epigenetic level in AML.

Our study assessed miRNA-mediated pathway interactions that are altered in AML patients. We identified that multiple miRNAs likely act together to regulate key biological pathways altered in AML. The implications of this finding suggest that AML targeted therapies focusing on single miRNAs may prove ineffective in some cases. Our data suggests that targeting of multiple miRNAs or their downstream pathways may be more effective. Future research will further evaluate transcriptomic changes induced by multiple AML-associated miRNAs, while considering AML patient subtypes as well as potential cytogenetic abnormalities. Understanding the epigenetically modified pathways underlying AML progression is extremely important, as increased pathophysiological understanding will foster the development of new effective therapies.

## 5. Conclusions

We used a systems-level approach to identify AML-associated biological pathways targeted for transcriptional control by a set of miRNAs. We found that AML-associated transcripts targeted by critical miRNAs interact in a highly significant manner. These interactions, when combined, form the AML Signalisome. Within this AML Signalisome, there is a significant enrichment for proteins of the AhR pathway and within hematopoiesis-related functions. These findings have implications for understanding biological pathways perturbed in disease that may inform targeted therapeutic strategy.

## Supplementary Files

Supplementary File 1: PDF-Document (PDF, 4309 KB)
