# Supplementary material for: The Aryl Hydrocarbon Receptor Pathway: A Key Component of the microRNA-Mediated AML Signalisome
Source: Int J Environ Res Public Health. 2012 May 18;9(5):1939–53. doi: 10.3390/ijerph9051939 (PMC3386597; doi:10.3390/ijerph9051939)
Supplement: Supplementary File 1: — PDF-Document (PDF, 4309 KB) [file ijerph-09-01939-s001.pdf]

Int. J. Environ. Res. Public Health **2012**, *9*, 1939-1953; doi:10.3390/ijerph9051939

OPEN ACCESS

International Journal of
Environmental Research and Public Health
ISSN 1660-4601
www.mdpi.com/journal/ijerph

Supplementary Information

The Aryl Hydrocarbon Receptor Pathway: A Key Component of the microRNA-Mediated AML Signalisome

Julia E. Rager [1] and Rebecca C. Fry [1,2,*]

[1] Department of Environmental Sciences and Engineering, Gillings School of Global Public Health, The University of North Carolina, 135 Dauer Drive, CB 7431, UNC, Chapel Hill, NC 27599, USA; E-Mail: jrager@live.unc.edu

[2] Curriculum in Toxicology, The University of North Carolina, 135 Dauer Drive, CB 7431, UNC, Chapel Hill, NC 27599, USA

* Author to whom correspondence should be addressed; E-Mail: rfry@unc.edu; Tel.: +1-919-843-6864; Fax: +1-919-843-9047.

Received: 21 March 2012; in revised form: 27 April 2012 / Accepted: 8 May 2012 / Published: 18 May 2012

Abstract: Recent research has spotlighted the role of microRNAs (miRNAs) as critical epigenetic regulators of hematopoietic stem cell differentiation and leukemia development. Despite the recent advances in knowledge surrounding epigenetics and leukemia, the mechanisms underlying miRNAs' influence on leukemia development have yet to be clearly elucidated. Our aim was to identify high ranking biological pathways altered at the gene expression level and under epigenetic control. Specifically, we set out to test the hypothesis that miRNAs dysregulated in acute myeloid leukemia (AML) converge on a common pathway that can influence signaling related to hematopoiesis and leukemia development. We identified genes altered in AML patients that are under common regulation of seven key miRNAs. By mapping these genes to a global interaction network, we identified the "AML Signalisome". The AML Signalisome comprises 53 AML-associated molecules, and is enriched for proteins that play a role in the aryl hydrocarbon receptor (AhR) pathway, a major regulator of hematopoiesis. Furthermore, we show biological enrichment for hematopoiesis-related proteins within the AML Signalisome. These findings provide important insight into miRNA-regulated pathways in leukemia, and may help to prioritize targets for disease prevention and treatment.

Keywords: aryl hydrocarbon receptor; gene expression; leukemia; microRNA; systems biology

---

Table of Contents

Table S1. Genes of the AML-associated gene expression signature. 731 unique genes (represented by 1119 probe sets) are listed, along with their corresponding fold change (FC) in expression (AML / non-leukemic).. 3

Table S2. 24 genes that overlap between the AML-associated gene expression signature and a gene signature used to predict AML survival, as identified in Metzeler *et al.* [1]......................... 29

Table S3. 1589 genes targeted by AML-associated miRNAs. All genes are listed alongside the miRNA(s) predicted to target each gene, and the probability of preferentially conserved targeting (PCT) for each predicted miRNA-mRNA interaction. 30

Table S4. The miRNA-mediated AML gene signature, consisting of 78 genes. 63

Table S5. Networks constructed using the miRNA-mediated AML gene signature..................... 65

Table S6. Biological functions and disease signatures associated with the AML Signalisome. 66

References 67

Table S1. Genes of the AML-associated gene expression signature. 731 unique genes (represented by 1119 probe sets) are listed, along with their corresponding fold change (FC) in expression (AML / non-leukemic).

Probe Set	Gene Symbol	Gene Title	Entrez Gene	*p*-value	q-value	FC
206067_s_at	*WT1*	Wilms tumor 1	7490	3.05E-09	2.05E-08	43.57
236892_s_at	*LOC100130740*	similar to hCG2042068	100130740	1.32E-10	1.15E-09	40.86
1553808_a_at	*NKX2-3*	NK2 transcription factor related, locus 3 (Drosophila)	159296	3.70E-10	2.94E-09	33.19
204561_x_at	*APOC2 / APOC4*	apolipoprotein C-II / apolipoprotein C-IV	344 / 346	5.73E-05	1.57E-04	32.65
205366_s_at	*HOXB6*	homeobox B6	3216	1.15E-12	1.55E-11	24.84
228885_at	*MAMDC2*	MAM domain containing 2	256691	7.29E-07	3.07E-06	22.40
213110_s_at	*COL4A5*	collagen, type IV, alpha 5	1287	1.12E-09	8.21E-09	19.22
1553613_s_at	*FOXC1*	forkhead box C1	2296	1.34E-04	3.38E-04	18.58
209905_at	*HOXA9*	homeobox A9	3205	4.97E-15	9.98E-14	16.81
226282_at				1.07E-03	2.13E-03	16.74
240766_at	*IL23A*	T cell receptor beta chain (TCRBV8S1-TCRBJ1S5)	51561	9.32E-05	2.43E-04	16.03
228904_at	*HOXB3*	homeobox B3	3213	8.77E-12	9.83E-11	15.20
235521_at	*HOXA3*	homeobox A3	3200	4.87E-13	7.03E-12	14.47
239791_at	*LOC100130740*	similar to hCG2042068	100130740	7.27E-10	5.50E-09	14.21
209488_s_at	*RBPMS*	RNA binding protein with multiple splicing	11030	7.50E-06	2.52E-05	13.52
214651_s_at	*HOXA9*	homeobox A9	3205	8.54E-20	3.65E-18	13.00
229638_at	*IRX3*	iroquois homeobox 3	79191	1.22E-05	3.92E-05	12.87
206674_at	*FLT3*	fms-related tyrosine kinase 3	2322	1.72E-17	5.21E-16	12.45
213844_at	*HOXA5*	homeobox A5	3202	6.01E-12	6.98E-11	12.27
238732_at	*COL24A1*	collagen, type XXIV, alpha 1	255631	4.01E-09	2.64E-08	11.76
1559477_s_at	*MEIS1*	Meis homeobox 1	4211	1.24E-11	1.34E-10	11.64
215382_x_at	*TPSAB1*	tryptase alpha/beta 1	7177	2.18E-04	5.22E-04	11.47
227923_at	*SHANK3*	SH3 and multiple ankyrin repeat domains 3	85358	8.17E-09	5.06E-08	11.23
210084_x_at	*TPSAB1*	tryptase alpha/beta 1	7177	1.79E-04	4.37E-04	11.18
243110_x_at	*NPW*	neuropeptide W	283869	5.84E-05	1.60E-04	11.08
209983_s_at	*NRXN2*	neurexin 2	9379	5.48E-09	3.51E-08	10.73
209757_s_at	*MYCN*	v-myc myelocytomatosis viral related oncogene, neuroblastoma derived (avian)	4613	2.17E-06	8.26E-06	10.69
207134_x_at	*TPSB2*	tryptase beta 2	64499	9.74E-05	2.53E-04	10.69
218899_s_at	*BAALC*	brain and acute leukemia, cytoplasmic	79870	1.02E-05	3.33E-05	10.67
205683_x_at	*TPSAB1 / TPSB2*	tryptase alpha/beta 1 / tryptase beta 2	64499 / 7177	6.09E-05	1.66E-04	10.32
206310_at	*SPINK2*	serine peptidase inhibitor, Kazal type 2 (acrosin-trypsin inhibitor)	6691	2.23E-13	3.41E-12	10.32
206847_s_at	*HOXA7*	homeobox A7	3204	6.93E-15	1.37E-13	10.21
216474_x_at	*TPSAB1 / TPSB2*	tryptase alpha/beta 1 / tryptase beta 2	64499 / 7177	9.67E-05	2.51E-04	10.11
213668_s_at	*SOX4*	SRY (sex determining region Y)-box 4	6659	2.09E-05	6.33E-05	9.86
209982_s_at	*NRXN2*	neurexin 2	9379	2.69E-08	1.50E-07	9.84
207741_x_at	*TPSAB1*	tryptase alpha/beta 1	7177	2.56E-04	6.01E-04	9.82
1553183_at	*UMODL1*	uromodulin-like 1	89766	5.63E-04	1.21E-03	9.61
223708_at	*C1QTNF4*	C1q and tumor necrosis factor related protein 4	114900	2.88E-08	1.60E-07	9.30
212776_s_at	*OBSL1*	obscurin-like 1	23363	1.82E-09	1.29E-08	9.22
201596_x_at	*KRT18*	keratin 18	3875	4.88E-07	2.13E-06	8.74
223627_at	*MEX3B*	mex-3 homolog B (C. elegans)	84206	2.09E-05	6.34E-05	8.66
226676_at	*ZNF521*	zinc finger protein 521	25925	3.02E-05	8.84E-05	8.57
229461_x_at	*NEGR1*	neuronal growth regulator 1	257194	3.26E-06	1.19E-05	8.47
204030_s_at	*SCHIP1*	schwannomin interacting protein 1	29970	7.66E-10	5.77E-09	8.34
220377_at	*FAM30A*	family with sequence similarity 30, member A	29064	3.67E-11	3.60E-10	8.32

Probe Set	Gene Symbol	Gene Title	Entrez Gene	*p*-value	q-value	FC
217023_x_at	*TPSAB1 / TPSB2*	tryptase alpha/beta 1 / tryptase beta 2	64499 / 7177	1.96E-04	4.74E-04	8.26
205899_at	*CCNA1*	cyclin A1	8900	7.36E-06	2.48E-05	8.09
209543_s_at	*CD34*	CD34 molecule	947	2.75E-07	1.26E-06	7.82
210239_at	*IRX5*	iroquois homeobox 5	10265	1.86E-04	4.52E-04	7.73
210664_s_at	*TFPI*	tissue factor pathway inhibitor (lipoprotein-associated coagulation inhibitor)	7035	3.81E-10	3.02E-09	7.67
205609_at	*ANGPT1*	angiopoietin 1	284	8.92E-09	5.48E-08	7.65
235753_at	*HOXA7*	homeobox A7	3204	2.28E-14	4.10E-13	7.63
205330_at	*MN1*	meningioma (disrupted in balanced translocation) 1	4330	7.04E-05	1.89E-04	7.53
222780_s_at	*BAALC*	brain and acute leukemia, cytoplasmic	79870	1.67E-04	4.10E-04	7.49
205601_s_at	*HOXB5*	homeobox B5	3215	1.02E-13	1.63E-12	7.46
222222_s_at	*HOMER3*	homer homolog 3 (Drosophila)	9454	5.58E-07	2.40E-06	7.45
230743_at	*LOC404266*	hypothetical LOC404266	404266	1.14E-10	1.00E-09	7.45
213150_at	*HOXA10*	homeobox A10	3206	3.06E-19	1.20E-17	7.42
235824_at				1.56E-05	4.87E-05	7.42
205600_x_at	*HOXB5*	homeobox B5	3215	6.33E-12	7.30E-11	7.40
230381_at	*C1orf186*	chromosome 1 open reading frame 186	440712	7.34E-05	1.96E-04	7.33
215116_s_at	*DNM1*	dynamin 1	1759	1.71E-09	1.21E-08	7.06
201655_s_at	*HSPG2*	heparan sulfate proteoglycan 2	3339	2.88E-06	1.07E-05	6.57
209487_at	*RBPMS*	RNA binding protein with multiple splicing	11030	4.57E-06	1.62E-05	6.53
226677_at	*ZNF521*	zinc finger protein 521	25925	2.36E-05	7.08E-05	6.51
1554974_at	*ACY3*	aspartoacylase (aminocyclase) 3	91703	2.05E-04	4.93E-04	6.48
201418_s_at	*SOX4*	SRY (sex determining region Y)-box 4	6659	1.72E-06	6.70E-06	6.46
210665_at	*TFPI*	tissue factor pathway inhibitor (lipoprotein-associated coagulation inhibitor)	7035	5.56E-08	2.92E-07	6.30
206298_at	*ARHGAP22*	Rho GTPase activating protein 22	58504	4.71E-07	2.07E-06	6.29
201431_s_at	*DPYSL3*	dihydropyrimidinase-like 3	1809	4.18E-04	9.31E-04	6.22
210237_at	*ARTN*	artemin	9048	7.96E-05	2.11E-04	6.14
210549_s_at	*CCL23*	chemokine (C-C motif) ligand 23	6368	2.53E-04	5.95E-04	6.10
205453_at	*HOXB2*	homeobox B2	3212	4.59E-11	4.42E-10	6.08
204304_s_at	*PROM1*	prominin 1	8842	5.09E-08	2.69E-07	6.06
231310_at				2.93E-07	1.34E-06	6.00
201069_at	*MMP2*	matrix metallopeptidase 2 (gelatinase A, 72kDa gelatinase, 72kDa type IV collagenase)	4313	5.65E-07	2.43E-06	6.00
219789_at	*NPR3*	natriuretic peptide receptor C/guanylate cyclase C (atrionatriuretic peptide receptor C)	4883	6.32E-07	2.69E-06	5.94
213260_at	*FOXC1*	forkhead box C1	2296	3.79E-04	8.53E-04	5.82
209576_at	*GNAI1*	guanine nucleotide binding protein (G protein), alpha inhibiting activity polypeptide 1	2770	9.15E-06	3.01E-05	5.82
238022_at	*hCG_1815491*	hCG1815491	643911	6.22E-09	3.95E-08	5.81
218825_at	*EGFL7*	EGF-like-domain, multiple 7	51162	1.63E-09	1.16E-08	5.77
206289_at	*HOXA4*	homeobox A4	3201	5.01E-11	4.78E-10	5.76
227860_at	*CPXM1*	carboxypeptidase X (M14 family), member 1	56265	2.13E-17	6.35E-16	5.75
204069_at	*MEIS1*	Meis homeobox 1	4211	8.41E-11	7.62E-10	5.73
221942_s_at	*GUCY1A3*	guanylate cyclase 1, soluble, alpha 3	2982	5.03E-07	2.19E-06	5.66
212775_at	*OBSL1*	obscurin-like 1	23363	2.29E-12	2.88E-11	5.64
241133_at				7.75E-06	2.59E-05	5.62
238021_s_at	*hCG_1815491*	hCG1815491	643911	2.66E-10	2.17E-09	5.51
227195_at	*ZNF503*	zinc finger protein 503	84858	4.15E-05	1.18E-04	5.51
219218_at	*BAHCC1*	BAH domain and coiled-coil containing 1	57597	6.69E-11	6.19E-10	5.50
214321_at	*NOV*	nephroblastoma overexpressed gene	4856	3.77E-05	1.08E-04	5.41

Probe Set	Gene Symbol	Gene Title	Entrez Gene	*p*-value	q-value	FC
213258_at	*TFPI*	tissue factor pathway inhibitor (lipoprotein-associated coagulation inhibitor)	7035	1.98E-12	2.53E-11	5.37
201416_at	*SOX4*	SRY (sex determining region Y)-box 4	6659	4.21E-14	7.23E-13	5.36
209386_at	*TM4SF1*	transmembrane 4 L six family member 1	4071	1.51E-04	3.76E-04	5.34
232424_at	*PRDM16*	PR domain containing 16	63976	1.38E-06	5.48E-06	5.28
243357_at	*NEGR1*	neuronal growth regulator 1	257194	4.48E-06	1.59E-05	5.21
211031_s_at	*CLIP2*	CAP-GLY domain containing linker protein 2	7461	1.62E-10	1.39E-09	5.18
236738_at	*LOC401097*	Similar to LOC166075	401097	1.97E-06	7.56E-06	5.16
201242_s_at	*ATP1B1*	ATPase, Na+/K+ transporting, beta 1 polypeptide	481	2.16E-12	2.73E-11	5.15
204548_at	*STAR*	steroidogenic acute regulatory protein	6770	8.66E-09	5.34E-08	5.15
219790_s_at	*NPR3*	natriuretic peptide receptor C/guanylate cyclase C (atrionatriuretic peptide receptor C)	4883	2.14E-06	8.15E-06	5.12
218686_s_at	*RHBDF1*	rhomboid 5 homolog 1 (Drosophila)	64285	1.73E-07	8.29E-07	4.94
210060_at	*PDE6G*	phosphodiesterase 6G, cGMP-specific, rod, gamma	5148	3.37E-10	2.69E-09	4.94
214455_at	*HIST1H2BC / HIST1H2BE / HIST1H2BF / HIST1H2BG / HIST1H2BI*	histone cluster 1, H2bc / histone cluster 1, H2be / histone cluster 1, H2bf / histone cluster 1, H2bg / histone cluster 1, H2bi	8339 / 8343 / 8344 / 8346 / 8347	2.31E-07	1.07E-06	4.89
209035_at	*MDK*	midkine (neurite growth-promoting factor 2)	4192	2.51E-05	7.47E-05	4.89
214639_s_at	*HOXA1*	homeobox A1	3198	8.95E-09	5.50E-08	4.88
218858_at	*DEPDC6*	DEP domain containing 6	64798	1.97E-10	1.65E-09	4.88
206478_at	*KIAA0125*	KIAA0125	9834	1.44E-05	4.54E-05	4.87
206726_at	*PGDS*	prostaglandin D2 synthase, hematopoietic	27306	1.89E-03	3.53E-03	4.86
204779_s_at	*HOXB7*	homeobox B7	3217	1.40E-06	5.56E-06	4.86
1554298_a_at	*WDR49*	WD repeat domain 49	151790	1.16E-07	5.73E-07	4.85
229530_at	*GUCY1A3*	guanylate cyclase 1, soluble, alpha 3	2982	4.53E-07	1.99E-06	4.83
205608_s_at	*ANGPT1*	angiopoietin 1	284	2.26E-09	1.57E-08	4.82
1559266_s_at	*C10orf140*	chromosome 10 open reading frame 140	387640	2.13E-05	6.45E-05	4.82
236193_at	*HIST1H2BC*	histone cluster 1, H2bc	8347	1.38E-06	5.49E-06	4.81
213823_at	*HOXA11*	homeobox A11	3207	8.53E-05	2.24E-04	4.81
239580_at	*GUCY1A3*	guanylate cyclase 1, soluble, alpha 3	2982	7.25E-05	1.94E-04	4.80
215034_s_at	*TM4SF1*	transmembrane 4 L six family member 1	4071	1.19E-04	3.03E-04	4.78
209387_s_at	*TM4SF1*	transmembrane 4 L six family member 1	4071	1.91E-04	4.62E-04	4.76
204468_s_at	*TIE1*	tyrosine kinase with immunoglobulin-like and EGF-like domains 1	7075	2.21E-08	1.26E-07	4.74
210033_s_at	*SPAG6*	sperm associated antigen 6	9576	2.07E-03	3.83E-03	4.69
210548_at	*CCL23*	chemokine (C-C motif) ligand 23	6368	8.31E-05	2.19E-04	4.69
226043_at	*GPSM1*	G-protein signaling modulator 1 (AGS3-like, C. elegans)	26086	1.18E-13	1.87E-12	4.68
213075_at	*OLFML2A*	olfactomedin-like 2A	169611	4.59E-04	1.01E-03	4.67
230266_at	*RAB7B*	RAB7B, member RAS oncogene family	338382	8.45E-05	2.23E-04	4.66
201015_s_at	*JUP*	junction plakoglobin	3728	6.16E-12	7.13E-11	4.66
239669_at				9.23E-04	1.87E-03	4.62
235171_at				2.45E-07	1.14E-06	4.61
227235_at	*GUCY1A3*	guanylate cyclase 1, soluble, alpha 3	2982	4.67E-06	1.65E-05	4.59
235142_at	*RP1-27O5.1 / ZBTB8*	zinc finger and BTB domain containing 8-like / zinc finger and BTB domain containing 8	653121 / 728116	2.69E-10	2.19E-09	4.58
222957_at	*NEU4*	sialidase 4	129807	1.18E-04	3.01E-04	4.55
207836_s_at	*RBPMS*	RNA binding protein with multiple splicing	11030	5.32E-06	1.85E-05	4.53
242172_at	*MEIS1*	Leukemogenic homolog protein (MEIS1)	4211	6.34E-07	2.70E-06	4.51
225842_at	*PHLDA1*	pleckstrin homology-like domain, family A, member 1	22822	7.98E-05	2.11E-04	4.48
219054_at	*C5orf23*	chromosome 5 open reading frame 23	79614	1.03E-06	4.21E-06	4.47

Probe Set	Gene Symbol	Gene Title	Entrez Gene	*p*-value	q-value	FC
227692_at	*GNAI1*	guanine nucleotide binding protein (G protein), alpha inhibiting activity polypeptide 1	2770	7.71E-05	2.05E-04	4.46
213147_at	*HOXA10*	homeobox A10	3206	2.57E-19	1.03E-17	4.38
202672_s_at	*ATF3*	activating transcription factor 3	467	1.21E-07	5.95E-07	4.35
205382_s_at	*CFD*	complement factor D (adipsin)	1675	3.95E-16	9.77E-15	4.33
229437_at	*BIC*	BIC transcript	114614	4.35E-09	2.84E-08	4.32
231365_at				8.29E-11	7.53E-10	4.31
243010_at	*MSI2*	musashi homolog 2 (Drosophila)	124540	8.86E-10	6.59E-09	4.30
225831_at	*LUZP1*	leucine zipper protein 1	7798	2.16E-13	3.32E-12	4.29
201243_s_at	*ATP1B1*	ATPase, Na+/K+ transporting, beta 1 polypeptide	481	2.28E-14	4.10E-13	4.27
215447_at	*TFPI*	Tissue factor pathway inhibitor (lipoprotein-associated coagulation inhibitor), mRNA (cDNA clone MGC:9251 IMAGE:3902987)	7035	2.16E-06	8.22E-06	4.27
218086_at	*NPDC1*	neural proliferation, differentiation and control, 1	56654	8.31E-08	4.21E-07	4.25
1558173_a_at	*LUZP1*	leucine zipper protein 1	7798	5.27E-12	6.18E-11	4.21
230481_at	*ACY3*	aspartoacylase (aminocyclase) 3	91703	1.24E-04	3.13E-04	4.19
229002_at	*FAM69B*	family with sequence similarity 69, member B	138311	6.47E-09	4.10E-08	4.18
1568838_at	*hCG_1742852 / LOC100132169*	hCG_1742852 / similar to hCG1742852	100128260 / 100132169	1.51E-04	3.75E-04	4.18
231767_at	*HOXB4*	homeobox B4	3214	1.43E-11	1.53E-10	4.18
207039_at	*CDKN2A*	cyclin-dependent kinase inhibitor 2A (melanoma, p16, inhibits CDK4)	1029	2.57E-03	4.62E-03	4.18
201564_s_at	*FSCN1*	fascin homolog 1, actin-bundling protein (Strongylocentrotus purpuratus)	6624	1.18E-14	2.24E-13	4.18
205309_at	*SMPDL3B*	sphingomyelin phosphodiesterase, acid-like 3B	27293	1.00E-06	4.10E-06	4.16
215489_x_at	*HOMER3*	homer homolog 3 (Drosophila)	9454	1.61E-10	1.37E-09	4.16
230670_at	*IGSF10*	immunoglobulin superfamily, member 10	285313	1.83E-07	8.72E-07	4.14
221831_at	*LUZP1*	leucine zipper protein 1	7798	2.61E-13	3.92E-12	4.13
232979_at				8.51E-06	2.82E-05	4.12
230894_s_at				6.07E-13	8.60E-12	4.10
204150_at	*STAB1*	stabilin 1	23166	2.25E-07	1.05E-06	4.10
204647_at	*HOMER3*	homer homolog 3 (Drosophila)	9454	1.97E-10	1.65E-09	4.09
204778_x_at	*HOXB7*	homeobox B7	3217	9.86E-09	6.00E-08	4.09
202609_at	*EPS8*	epidermal growth factor receptor pathway substrate 8	2059	1.28E-05	4.08E-05	4.08
209676_at	*TFPI*	tissue factor pathway inhibitor (lipoprotein-associated coagulation inhibitor)	7035	2.57E-11	2.60E-10	4.07
225962_at	*ZNRF1*	zinc and ring finger 1	84937	1.34E-10	1.17E-09	4.07
1553194_at	*NEGR1*	neuronal growth regulator 1	257194	4.34E-06	1.55E-05	4.06
236898_at				6.18E-06	2.12E-05	4.06
38487_at	*STAB1*	stabilin 1	23166	4.30E-07	1.90E-06	4.04
1554300_a_at	*SVOPL*	SVOP-like	136306	1.52E-04	3.76E-04	4.04
219631_at	*LRP12*	low density lipoprotein-related protein 12	29967	4.30E-07	1.90E-06	4.03
242051_at				5.19E-08	2.74E-07	4.03
204810_s_at	*CKM*	creatine kinase, muscle	1158	1.01E-08	6.11E-08	4.02
243617_at	*ZNF827*	CDNA clone IMAGE:5440683	152485	2.62E-03	4.72E-03	4.01
206148_at	*IL3RA*	interleukin 3 receptor, alpha (low affinity)	3563	8.37E-09	5.17E-08	3.99
200923_at	*LGALS3BP*	lectin, galactoside-binding, soluble, 3 binding protein	3959	3.02E-07	1.38E-06	3.98
1554980_a_at	*ATF3*	activating transcription factor 3	467	4.06E-05	1.15E-04	3.97
201417_at	*SOX4*	SRY (sex determining region Y)-box 4	6659	6.79E-20	2.98E-18	3.97
238498_at				1.78E-08	1.03E-07	3.93
219837_s_at	*CYTL1*	cytokine-like 1	54360	1.87E-06	7.20E-06	3.92
226132_s_at	*MANEAL*	mannosidase, endo-alpha-like	149175	2.02E-09	1.41E-08	3.90

Probe Set	Gene Symbol	Gene Title	Entrez Gene	*p*-value	q-value	FC
205051_s_at	*KIT*	v-kit Hardy-Zuckerman 4 feline sarcoma viral oncogene homolog	3815	7.40E-10	5.59E-09	3.86
205801_s_at	*RASGRP3*	RAS guanyl releasing protein 3 (calcium and DAG-regulated)	25780	8.91E-07	3.68E-06	3.86
244413_at	*CLECL1*	C-type lectin-like 1	160365	2.13E-03	3.92E-03	3.86
201325_s_at	*EMP1*	epithelial membrane protein 1	2012	1.42E-05	4.49E-05	3.85
243092_at				6.66E-08	3.45E-07	3.85
232370_at	*LOC254057*	hypothetical protein LOC254057	254057	5.87E-04	1.26E-03	3.85
1552623_at	*HSH2D*	hematopoietic SH2 domain containing	84941	2.44E-13	3.70E-12	3.82
216973_s_at	*HOXB7*	homeobox B7	3217	6.78E-07	2.87E-06	3.81
229739_s_at	*FAM116B*	family with sequence similarity 116, member B	414918	1.22E-16	3.28E-15	3.81
228678_at	*FAM116B*	family with sequence similarity 116, member B	414918	3.25E-18	1.12E-16	3.79
229309_at	*ADRB1*	adrenergic, beta-1-, receptor	153	1.95E-06	7.49E-06	3.79
201028_s_at	*CD99*	CD99 molecule	4267	1.37E-16	3.65E-15	3.79
237311_at				5.58E-06	1.93E-05	3.78
238097_at	*LOC100134226*	hypothetical protein LOC100134226	100134226	7.11E-10	5.39E-09	3.78
220253_s_at	*LRP12*	low density lipoprotein-related protein 12	29967	4.89E-07	2.13E-06	3.77
225233_at	*MSI2*	musashi homolog 2 (Drosophila)	124540	9.38E-10	6.95E-09	3.77
210365_at	*RUNX1*	runt-related transcription factor 1	861	7.54E-13	1.05E-11	3.76
235438_at				1.64E-06	6.40E-06	3.76
206106_at	*MAPK12*	mitogen-activated protein kinase 12	6300	3.40E-19	1.33E-17	3.76
211181_x_at	*RUNX1*	runt-related transcription factor 1	861	1.88E-04	4.57E-04	3.76
1554876_a_at	*S100Z*	S100 calcium binding protein Z	170591	1.34E-09	9.68E-09	3.74
228274_at	*SDSL*	serine dehydratase-like	113675	1.30E-09	9.43E-09	3.74
231233_at				4.57E-13	6.62E-12	3.73
230541_at	*LOC149134*	hypothetical LOC149134	149134	2.25E-05	6.76E-05	3.73
216705_s_at	*ADA*	adenosine deaminase	100	3.65E-10	2.90E-09	3.72
219686_at	*STK32B*	serine/threonine kinase 32B	55351	2.71E-06	1.01E-05	3.71
212089_at	*LMNA*	lamin A/C	4000	1.17E-06	4.73E-06	3.70
1555923_a_at	*C10orf114*	chromosome 10 open reading frame 114	399726	7.18E-08	3.68E-07	3.69
225240_s_at	*MSI2*	musashi homolog 2 (Drosophila)	124540	4.10E-11	3.99E-10	3.68
205349_at	*GNA15*	guanine nucleotide binding protein (G protein), alpha 15 (Gq class)	2769	4.99E-17	1.42E-15	3.67
201324_at	*EMP1*	epithelial membrane protein 1	2012	2.82E-06	1.05E-05	3.67
240180_at				8.89E-10	6.61E-09	3.66
221814_at	*GPR124*	G protein-coupled receptor 124	25960	2.18E-06	8.29E-06	3.64
211499_s_at	*MAPK11*	mitogen-activated protein kinase 11	5600	2.92E-10	2.36E-09	3.62
214522_x_at	*HIST1H2AD / HIST1H3D*	histone cluster 1, H2ad / histone cluster 1, H3d	3013 / 8351	3.63E-06	1.31E-05	3.61
219602_s_at	*FAM38B*	family with sequence similarity 38, member B	63895	1.26E-04	3.18E-04	3.60
227711_at	*GTSF1*	gametocyte specific factor 1	121355	3.18E-06	1.17E-05	3.55
232636_at	*SLITRK4*	SLIT and NTRK-like family, member 4	139065	5.97E-11	5.59E-10	3.54
221563_at	*DUSP10*	dual specificity phosphatase 10	11221	2.48E-13	3.75E-12	3.52
225237_s_at	*MSI2*	musashi homolog 2 (Drosophila)	124540	9.70E-11	8.70E-10	3.51
204501_at	*NOV*	nephroblastoma overexpressed gene	4856	1.30E-03	2.53E-03	3.51
218338_at	*PHC1 / PHC1B*	polyhomeotic homolog 1 (Drosophila) / polyhomeotic homolog 1B (Drosophila)	1911 / 653441	1.50E-15	3.35E-14	3.49
230896_at	*BEND4*	BEN domain containing 4	389206	9.96E-05	2.58E-04	3.46
208557_at	*HOXA6*	homeobox A6	3203	9.38E-12	1.05E-10	3.45
220450_at				7.27E-09	4.56E-08	3.43
203920_at	*NR1H3*	nuclear receptor subfamily 1, group H, member 3	10062	4.73E-16	1.16E-14	3.43
1564151_at				1.16E-09	8.49E-09	3.40

Probe Set	Gene Symbol	Gene Title	Entrez Gene	*p*-value	q-value	FC
239562_at	*MTHFD2L*	methylenetetrahydrofolate dehydrogenase (NADP+ dependent) 2-like	441024	1.18E-11	1.28E-10	3.40
209710_at	*GATA2*	GATA binding protein 2	2624	8.60E-09	5.31E-08	3.39
201037_at	*PFKP*	phosphofructokinase, platelet	5214	1.22E-16	3.28E-15	3.37
213779_at	*EMID1*	EMI domain containing 1	129080	7.20E-16	1.70E-14	3.36
230977_at	*NPM2*	nucleophosmin/nucleoplasmin, 2	10361	2.92E-15	6.08E-14	3.36
227556_at	*NME7*	non-metastatic cells 7, protein expressed in (nucleoside-diphosphate kinase)	29922	7.17E-11	6.59E-10	3.35
231369_at	*ZNF333*	MRNA; cDNA DKFZp547F2317 (from clone DKFZp547F2317)	84449	2.89E-07	1.32E-06	3.35
215501_s_at	*DUSP10*	dual specificity phosphatase 10	11221	1.42E-10	1.23E-09	3.34
202023_at	*EFNA1*	ephrin-A1	1942	5.17E-04	1.12E-03	3.34
210933_s_at	*FSCN1*	fascin homolog 1, actin-bundling protein (Strongylocentrotus purpuratus)	6624	2.39E-21	1.32E-19	3.34
208690_s_at	*PDLIM1*	PDZ and LIM domain 1	9124	4.08E-14	7.03E-13	3.33
211182_x_at	*RUNX1*	runt-related transcription factor 1	861	1.02E-04	2.65E-04	3.33
226125_at				6.84E-06	2.32E-05	3.32
215506_s_at	*DIRAS3*	DIRAS family, GTP-binding RAS-like 3	9077	9.69E-05	2.52E-04	3.32
1558871_at				2.14E-06	8.15E-06	3.31
225615_at	*IFFO2*	intermediate filament family orphan 2	126917	4.82E-09	3.12E-08	3.29
203946_s_at	*ARG2*	arginase, type II	384	4.07E-05	1.16E-04	3.29
218876_at	*TPPP3*	tubulin polymerization-promoting protein family member 3	51673	1.82E-03	3.41E-03	3.29
207550_at	*MPL*	myeloproliferative leukemia virus oncogene	4352	6.84E-06	2.32E-05	3.28
218641_at	*MGC3032*	hypothetical protein MGC3032	65998	1.73E-11	1.81E-10	3.28
240065_at	*FAM81B*	family with sequence similarity 81, member B	153643	8.41E-07	3.49E-06	3.28
208579_x_at	*H2BFS*	H2B histone family, member S	54145	4.94E-10	3.84E-09	3.27
225436_at	*FAM108C1*	family with sequence similarity 108, member C1	58489	2.19E-03	4.02E-03	3.27
206907_at	*TNFSF9*	tumor necrosis factor (ligand) superfamily, member 9	8744	1.54E-05	4.81E-05	3.27
215537_x_at	*DDAH2*	dimethylarginine dimethylaminohydrolase 2	23564	2.90E-14	5.10E-13	3.26
221832_s_at	*LUZP1*	leucine zipper protein 1	7798	4.61E-12	5.46E-11	3.25
1553247_a_at	*ZNF709*	zinc finger protein 709	163051	5.81E-10	4.46E-09	3.24
218788_s_at	*SMYD3*	SET and MYND domain containing 3	64754	8.50E-11	7.69E-10	3.23
214228_x_at	*TNFRSF4*	tumor necrosis factor receptor superfamily, member 4	7293	9.85E-04	1.98E-03	3.22
229971_at	*GPR114*	G protein-coupled receptor 114	221188	2.16E-09	1.50E-08	3.22
205227_at	*IL1RAP*	interleukin 1 receptor accessory protein	3556	2.13E-08	1.22E-07	3.21
244533_at				2.21E-04	5.27E-04	3.21
204639_at	*ADA*	adenosine deaminase	100	1.77E-13	2.75E-12	3.21
224710_at	*RAB34*	RAB34, member RAS oncogene family	83871	1.13E-16	3.06E-15	3.21
232051_at	*CCDC102A*	coiled-coil domain containing 102A	92922	1.24E-09	9.03E-09	3.18
225778_at	*RBMS2*	RNA binding motif, single stranded interacting protein 2	5939	3.88E-10	3.07E-09	3.18
239457_at	*ATP8B3*	ATPase, class I, type 8B, member 3	148229	2.57E-05	7.64E-05	3.18
226192_at				1.17E-03	2.31E-03	3.18
226461_at	*HOXB9*	homeobox B9	3219	1.53E-03	2.93E-03	3.18
226134_s_at				3.03E-12	3.71E-11	3.17
240084_at	*CBX2*	chromobox homolog 2 (Pc class homolog, Drosophila)	84733	4.74E-12	5.61E-11	3.17
202262_x_at	*DDAH2*	dimethylarginine dimethylaminohydrolase 2	23564	2.72E-14	4.81E-13	3.16
213035_at	*ANKRD28*	ankyrin repeat domain 28	23243	1.33E-08	7.91E-08	3.16
212013_at	*PXDN*	peroxidasin homolog (Drosophila)	7837	5.02E-04	1.09E-03	3.16
212012_at	*PXDN*	peroxidasin homolog (Drosophila)	7837	2.00E-03	3.71E-03	3.15
203476_at	*TPBG*	trophoblast glycoprotein	7162	5.10E-06	1.78E-05	3.15

Probe Set	Gene Symbol	Gene Title	Entrez Gene	*p*-value	q-value	FC
203819_s_at	*IGF2BP3*	insulin-like growth factor 2 mRNA binding protein 3	10643	1.81E-07	8.61E-07	3.14
224851_at	*CDK6*	cyclin-dependent kinase 6	1021	9.11E-10	6.76E-09	3.14
206960_at	*LPAR4*	lysophosphatidic acid receptor 4	2846	2.28E-10	1.88E-09	3.14
207160_at	*IL12A*	interleukin 12A (natural killer cell stimulatory factor 1, cytotoxic lymphocyte maturation factor 1, p35)	3592	2.17E-09	1.51E-08	3.13
207850_at	*CXCL3*	chemokine (C-X-C motif) ligand 3	2921	4.12E-05	1.17E-04	3.13
206574_s_at	*LOC100131062 / PTP4A3*	similar to protein tyrosine phosphatase type IVA, member 3 / protein tyrosine phosphatase type IVA, member 3	100131062 / 11156	1.44E-05	4.54E-05	3.13
203349_s_at	*ETV5*	ets variant 5	2119	7.90E-05	2.10E-04	3.12
203367_at	*DUSP14*	dual specificity phosphatase 14	11072	6.97E-14	1.15E-12	3.11
222240_s_at	*ISYNA1*	inositol-3-phosphate synthase 1	51477	1.59E-15	3.51E-14	3.11
214729_at	*TWISTNB*	TWIST neighbor	221830	7.21E-11	6.63E-10	3.10
229307_at	*ANKRD28*	ankyrin repeat domain 28	23243	2.06E-14	3.74E-13	3.10
202820_at	*AHR*	aryl hydrocarbon receptor	196	2.23E-09	1.55E-08	3.09
209806_at	*HIST1H2BK*	histone cluster 1, H2bk	85236	6.27E-20	2.78E-18	3.09
230348_at	*LATS2*	LATS, large tumor suppressor, homolog 2 (Drosophila)	26524	4.88E-08	2.59E-07	3.09
208029_s_at	*LAPTM4B*	lysosomal protein transmembrane 4 beta	55353	1.02E-05	3.33E-05	3.08
232227_at				1.89E-03	3.53E-03	3.07
202016_at	*MEST*	mesoderm specific transcript homolog (mouse)	4232	3.16E-06	1.16E-05	3.06
65718_at	*GPR124*	G protein-coupled receptor 124	25960	3.92E-06	1.41E-05	3.06
219557_s_at	*NRIP3*	nuclear receptor interacting protein 3	56675	3.60E-06	1.30E-05	3.05
201029_s_at	*CD99*	CD99 molecule	4267	3.30E-22	2.02E-20	3.05
227055_at	*METTL7B*	methyltransferase like 7B	196410	1.59E-04	3.93E-04	3.05
235287_at	*CDK6*	cyclin-dependent kinase 6	1021	2.36E-09	1.63E-08	3.05
244397_at				5.18E-15	1.04E-13	3.04
242832_at	*PER1*	period homolog 1 (Drosophila)	5187	5.76E-05	1.58E-04	3.04
242269_at	*FLJ42875*	hypothetical LOC440556	440556	8.99E-08	4.53E-07	3.03
216052_x_at	*ARTN*	artemin	9048	5.56E-05	1.53E-04	3.02
235309_at	*RPS15A*	Ribosomal protein S15a	6210	3.71E-16	9.23E-15	3.00
209235_at	*CLCN7*	chloride channel 7	1186	1.82E-12	2.34E-11	3.00
1552908_at	*C1orf150*	chromosome 1 open reading frame 150	148823	2.47E-05	7.37E-05	3.00
211597_s_at	*HOPX*	HOP homeobox	84525	1.66E-06	6.49E-06	3.00
201939_at	*PLK2*	polo-like kinase 2 (Drosophila)	10769	3.15E-05	9.16E-05	3.00
224767_at	*RPL37*	Ribosomal protein L37, mRNA (cDNA clone IMAGE:6671394)	6167	9.36E-20	4.00E-18	3.00
205193_at	*MAFF*	v-maf musculoaponeurotic fibrosarcoma oncogene homolog F (avian)	23764	4.42E-07	1.95E-06	2.99
215146_s_at	*TTC28*	tetratricopeptide repeat domain 28	23331	2.03E-07	9.59E-07	2.99
220952_s_at	*PLEKHA5*	pleckstrin homology domain containing, family A member 5	54477	3.93E-13	5.74E-12	2.98
242028_at	*ZNF709*	zinc finger protein 709	163051	3.65E-09	2.43E-08	2.98
208303_s_at	*CRLF2*	cytokine receptor-like factor 2	64109	1.37E-03	2.65E-03	2.98
232151_at	*MACC1*	metastasis associated in colon cancer 1	346389	4.44E-04	9.82E-04	2.97
241150_at	*SPTAN1*	Alpha II spectrin	6709	1.27E-07	6.21E-07	2.96
217997_at	*PHLDA1*	pleckstrin homology-like domain, family A, member 1	22822	1.49E-03	2.87E-03	2.96
227574_at	*OBSL1*	obscurin-like 1	23363	5.22E-09	3.36E-08	2.96
236281_x_at	*HTR7*	5-hydroxytryptamine (serotonin) receptor 7 (adenylate cyclase-coupled)	3363	1.43E-07	6.96E-07	2.95
200986_at	*SERPING1*	serpin peptidase inhibitor, clade G (C1 inhibitor), member 1	710	2.34E-11	2.39E-10	2.95
223575_at	*KIAA1549*	KIAA1549	57670	4.96E-13	7.14E-12	2.95

Probe Set	Gene Symbol	Gene Title	Entrez Gene	*p*-value	q-value	FC
205081_at	*CRIP1*	cysteine-rich protein 1 (intestinal)	1396	1.28E-09	9.30E-09	2.95
208527_x_at	*HIST1H2BC / HIST1H2BE / HIST1H2BF / HIST1H2BG / HIST1H2BI*	histone cluster 1, H2bc / histone cluster 1, H2be / histone cluster 1, H2bf / histone cluster 1, H2bg / histone cluster 1, H2bi	8339 / 8343 / 8344 / 8346 / 8347	3.78E-09	2.50E-08	2.94
214378_at	*TFPI*	tissue factor pathway inhibitor (lipoprotein-associated coagulation inhibitor)	7035	1.00E-10	8.93E-10	2.94
1554489_a_at	*CEP70*	centrosomal protein 70kDa	80321	2.93E-08	1.63E-07	2.93
212086_x_at	*LMNA*	lamin A/C	4000	1.93E-07	9.13E-07	2.92
1552474_a_at	*GAMT*	guanidinoacetate N-methyltransferase	2593	2.62E-17	7.73E-16	2.92
209344_at	*TPM4*	tropomyosin 4	7171	2.61E-09	1.78E-08	2.90
219559_at	*C20orf59*	chromosome 20 open reading frame 59	63910	7.89E-18	2.53E-16	2.90
219550_at	*ROBO3*	roundabout, axon guidance receptor, homolog 3 (Drosophila)	64221	6.91E-09	4.36E-08	2.90
216417_x_at	*HOXB9*	homeobox B9	3219	1.03E-11	1.14E-10	2.90
225238_at	*MSI2*	musashi homolog 2 (Drosophila)	124540	1.24E-05	3.98E-05	2.90
1560029_a_at	*C11orf57*	chromosome 11 open reading frame 57	55216	8.52E-10	6.36E-09	2.89
209803_s_at	*PHLDA2*	pleckstrin homology-like domain, family A, member 2	7262	3.70E-04	8.35E-04	2.89
237483_at				1.90E-03	3.54E-03	2.89
239066_at				1.24E-07	6.08E-07	2.89
214909_s_at	*DDAH2*	dimethylarginine dimethylaminohydrolase 2	23564	1.43E-15	3.20E-14	2.89
242260_at	*MATR3*	Major nuclear matrix protein mRNA	9782	4.22E-09	2.77E-08	2.89
243618_s_at	*ZNF827*	CDNA clone IMAGE:5440683	152485	2.41E-03	4.37E-03	2.89
205839_s_at	*BZRAP1*	benzodiazapine receptor (peripheral) associated protein 1	9256	9.92E-07	4.06E-06	2.88
203820_s_at	*IGF2BP3*	insulin-like growth factor 2 mRNA binding protein 3	10643	2.72E-09	1.85E-08	2.88
1559276_at	*LOC728606*	hypothetical LOC728606	728606	1.63E-05	5.07E-05	2.88
228083_at	*CACNA2D4*	calcium channel, voltage-dependent, alpha 2/delta subunit 4	93589	4.63E-06	1.64E-05	2.88
204363_at	*F3*	coagulation factor III (thromboplastin, tissue factor)	2152	2.71E-04	6.32E-04	2.88
206020_at	*SOCS6*	suppressor of cytokine signaling 6	9306	8.96E-15	1.72E-13	2.88
1554660_a_at	*C1orf71*	chromosome 1 open reading frame 71	163882	2.32E-17	6.87E-16	2.87
205551_at	*SV2B*	synaptic vesicle glycoprotein 2B	9899	3.62E-05	1.04E-04	2.87
206072_at	*UCN*	urocortin	7349	2.39E-07	1.11E-06	2.87
230795_at				2.19E-07	1.02E-06	2.87
216953_s_at	*WT1*	Wilms tumor 1	7490	3.07E-07	1.40E-06	2.87
219201_s_at	*TWSG1*	twisted gastrulation homolog 1 (Drosophila)	57045	2.30E-10	1.90E-09	2.87
227875_at	*KLHL13*	kelch-like 13 (Drosophila)	90293	3.36E-05	9.71E-05	2.86
217226_s_at	*SFXN3*	sideroflexin 3	81855	1.20E-15	2.71E-14	2.86
1555630_a_at	*RAB34*	RAB34, member RAS oncogene family	83871	1.90E-16	4.96E-15	2.86
211267_at	*HESX1*	HESX homeobox 1	8820	4.23E-10	3.32E-09	2.85
223075_s_at	*AIF1L*	allograft inflammatory factor 1-like	83543	4.83E-05	1.35E-04	2.85
215111_s_at	*TSC22D1*	TSC22 domain family, member 1	8848	1.11E-10	9.80E-10	2.85
224150_s_at	*CEP70*	centrosomal protein 70kDa	80321	5.37E-10	4.15E-09	2.85
210755_at	*HGF*	hepatocyte growth factor (hepapoietin A; scatter factor)	3082	7.35E-04	1.53E-03	2.84
220016_at	*AHNAK*	AHNAK nucleoprotein	79026	1.71E-06	6.66E-06	2.84
219569_s_at	*TMEM22*	transmembrane protein 22	80723	9.14E-07	3.77E-06	2.84
214873_at	*LRP5L*	low density lipoprotein receptor-related protein 5-like	91355	1.06E-05	3.45E-05	2.83
200962_at	*RPL31*	ribosomal protein L31	6160	3.97E-12	4.75E-11	2.83
238389_s_at				5.86E-15	1.16E-13	2.83
244555_at				1.12E-10	9.87E-10	2.83

Probe Set	Gene Symbol	Gene Title	Entrez Gene	*p*-value	q-value	FC
224901_at	*SCD5*	stearoyl-CoA desaturase 5	79966	1.14E-08	6.83E-08	2.83
225335_at	*ZNF496*	zinc finger protein 496	84838	3.29E-18	1.13E-16	2.81
205624_at	*CPA3*	carboxypeptidase A3 (mast cell)	1359	8.99E-06	2.96E-05	2.81
1552364_s_at	*MSI2*	musashi homolog 2 (Drosophila)	124540	1.97E-11	2.04E-10	2.81
220668_s_at	*DNMT3B*	DNA (cytosine-5-)-methyltransferase 3 beta	1789	1.47E-08	8.66E-08	2.80
205997_at	*ADAM28*	ADAM metallopeptidase domain 28	10863	1.07E-10	9.51E-10	2.80
210805_x_at	*RUNX1*	runt-related transcription factor 1	861	4.40E-09	2.88E-08	2.80
228011_at	*FAM92A1*	family with sequence similarity 92, member A1	137392	8.58E-07	3.55E-06	2.80
203759_at	*ST3GAL4*	ST3 beta-galactoside alpha-2,3-sialyltransferase 4	6484	2.10E-13	3.23E-12	2.80
236635_at	*ZNF667*	zinc finger protein 667	63934	5.58E-09	3.57E-08	2.79
206134_at	*ADAMDEC1*	ADAM-like, decysin 1	27299	6.71E-05	1.81E-04	2.79
1567107_s_at	*TPM4*	tropomyosin 4	7171	4.86E-11	4.66E-10	2.79
237470_at	*DOCK7*	dedicator of cytokinesis 7	85440	8.47E-12	9.53E-11	2.78
201063_at	*RCN1*	reticulocalbin 1, EF-hand calcium binding domain	5954	1.43E-20	7.08E-19	2.78
1558105_a_at				5.07E-11	4.82E-10	2.78
226025_at	*ANKRD28*	ankyrin repeat domain 28	23243	3.80E-16	9.42E-15	2.78
226550_at				4.17E-12	4.97E-11	2.78
229095_s_at	*LOC440895*	LIM and senescent cell antigen-like domains 3-like	440895	6.75E-05	1.82E-04	2.78
208614_s_at	*FLNB*	filamin B, beta (actin binding protein 278)	2317	6.56E-05	1.78E-04	2.77
222900_at				9.78E-08	4.90E-07	2.77
229140_at	*ZNF579*	zinc finger protein 579	163033	6.24E-17	1.74E-15	2.77
225308_s_at	*TANC1*	tetratricopeptide repeat, ankyrin repeat and coiled-coil containing 1	85461	8.35E-04	1.71E-03	2.77
244610_x_at				7.91E-05	2.10E-04	2.76
220911_s_at	*KIAA1305*	KIAA1305	57523	7.46E-07	3.13E-06	2.76
209365_s_at	*ECM1*	extracellular matrix protein 1	1893	2.24E-11	2.29E-10	2.76
225418_at	*PVRL2*	poliovirus receptor-related 2 (herpesvirus entry mediator B)	5819	1.40E-13	2.20E-12	2.76
226858_at	*CSNK1E*	casein kinase 1, epsilon	1454	3.86E-09	2.55E-08	2.76
232269_x_at	*METRN*	meteorin, glial cell differentiation regulator	79006	8.27E-06	2.75E-05	2.76
205354_at	*GAMT*	guanidinoacetate N-methyltransferase	2593	3.66E-17	1.06E-15	2.75
205518_s_at	*CMAH*	cytidine monophosphate-N-acetylneuraminic acid hydroxylase (CMP-N-acetylneuraminate monooxygenase) pseudogene	8418	2.30E-12	2.89E-11	2.75
226899_at	*UNC5B*	unc-5 homolog B (C. elegans)	219699	4.62E-05	1.30E-04	2.75
226545_at	*CD109*	CD109 molecule	135228	1.25E-04	3.15E-04	2.75
1554679_a_at	*LAPTM4B*	lysosomal protein transmembrane 4 beta	55353	3.36E-05	9.70E-05	2.74
202701_at	*BMP1*	bone morphogenetic protein 1	649	3.49E-22	2.13E-20	2.74
208890_s_at	*PLXNB2*	plexin B2	23654	2.29E-13	3.49E-12	2.74
216331_at	*ITGA7*	integrin, alpha 7	3679	1.29E-03	2.51E-03	2.74
238444_at	*ZNF618*	zinc finger protein 618	114991	1.83E-06	7.10E-06	2.74
206954_at	*WIT1*	Wilms tumor upstream neighbor 1	51352	4.90E-10	3.82E-09	2.73
243000_at	*CDK6*	cyclin-dependent kinase 6	1021	3.31E-13	4.90E-12	2.72
221458_at	*HTR1F*	5-hydroxytryptamine (serotonin) receptor 1F	3355	8.94E-06	2.95E-05	2.72
243579_at	*MSI2*	musashi homolog 2 (Drosophila)	124540	8.05E-11	7.33E-10	2.72
235500_at	*HNRNPC*	heterogeneous nuclear ribonucleoprotein C (C1/C2)	3183	2.35E-05	7.04E-05	2.71
205239_at	*AREG / AREGB*	amphiregulin / amphiregulin B	374 / 727738	7.28E-07	3.06E-06	2.71
224996_at	*ASPH*	Aspartyl(asparaginyl)beta-hydroxylase [human, hepatoblastoma cell line HepG2, mRNA Partial, 2324 nt]	444	1.57E-15	3.47E-14	2.71
202708_s_at	*HIST2H2BE*	histone cluster 2, H2be	8349	3.77E-08	2.05E-07	2.71
226817_at	*DSC2*	desmocollin 2	1824	6.10E-04	1.30E-03	2.71

Probe Set	Gene Symbol	Gene Title	Entrez Gene	*p*-value	q-value	FC
210299_s_at	*FHL1*	four and a half LIM domains 1	2273	4.36E-04	9.66E-04	2.70
240151_at	*LOC100130740*	similar to hCG2042068	100130740	2.22E-08	1.27E-07	2.70
229620_at	*SEPP1*	Selenoprotein P, plasma, 1, mRNA (cDNA clone MGC:47713 IMAGE:5770225)	6414	2.17E-05	6.56E-05	2.70
44040_at	*FBXO41*	F-box protein 41	150726	1.79E-17	5.41E-16	2.70
213058_at	*TTC28*	tetratricopeptide repeat domain 28	23331	9.33E-09	5.71E-08	2.70
226517_at	*BCAT1*	branched chain aminotransferase 1, cytosolic	586	6.34E-09	4.02E-08	2.70
224763_at	*RPL37*	Ribosomal protein L37, mRNA (cDNA clone IMAGE:6671394)	6167	1.29E-18	4.73E-17	2.69
1559617_at	*LOC732087*	hypothetical protein LOC732087	732087	8.46E-05	2.23E-04	2.69
217165_x_at	*MT1F*	metallothionein 1F	4494	1.15E-06	4.66E-06	2.69
242426_at	*NRG4*	neuregulin 4	145957	2.59E-09	1.77E-08	2.69
223284_at	*NAT14*	N-acetyltransferase 14 (GCN5-related, putative)	57106	1.54E-14	2.87E-13	2.68
219871_at	*FLJ13197*	hypothetical FLJ13197	79667	2.85E-08	1.59E-07	2.68
238127_at	*FLJ41484*	hypothetical LOC650669	650669	1.07E-07	5.32E-07	2.68
230127_at				1.86E-08	1.07E-07	2.68
233749_at	*LOC100129014*	hypothetical protein LOC100129014	100129014	3.53E-04	8.00E-04	2.68
220974_x_at	*SFXN3*	sideroflexin 3	81855	4.99E-16	1.21E-14	2.68
207636_at	*SERPINI2*	serpin peptidase inhibitor, clade I (pancpin), member 2	5276	4.18E-10	3.28E-09	2.67
222774_s_at	*NETO2*	neuropilin (NRP) and tolloid (TLL)-like 2	81831	2.19E-10	1.81E-09	2.67
224746_at	*KIAA1522*	KIAA1522	57648	7.17E-12	8.18E-11	2.67
211071_s_at	*MLLT11*	myeloid/lymphoid or mixed-lineage leukemia (trithorax homolog, Drosophila); translocated to, 11	10962	2.95E-06	1.09E-05	2.67
202933_s_at	*YES1*	v-yes-1 Yamaguchi sarcoma viral oncogene homolog 1	7525	1.71E-06	6.66E-06	2.67
1554242_a_at	*COCH*	coagulation factor C homolog, cochlin (Limulus polyphemus)	1690	4.26E-05	1.20E-04	2.67
219884_at	*LHX6*	LIM homeobox 6	26468	2.25E-04	5.38E-04	2.66
1553982_a_at	*RAB7B*	RAB7B, member RAS oncogene family	338382	4.35E-06	1.55E-05	2.66
225510_at	*OAF*	OAF homolog (Drosophila)	220323	1.04E-05	3.38E-05	2.66
224049_at	*KCNK17*	potassium channel, subfamily K, member 17	89822	4.86E-10	3.79E-09	2.66
238741_at	*FAM83A*	family with sequence similarity 83, member A	84985	2.61E-03	4.69E-03	2.65
230655_at				9.13E-05	2.39E-04	2.65
228113_at	*RAB37*	RAB37, member RAS oncogene family	326624	3.11E-16	7.84E-15	2.65
34726_at	*CACNB3*	calcium channel, voltage-dependent, beta 3 subunit	784	5.26E-10	4.07E-09	2.65
226071_at	*ADAMTSL4*	ADAMTS-like 4	54507	6.77E-07	2.87E-06	2.64
221560_at	*MARK4*	MAP/microtubule affinity-regulating kinase 4	57787	7.59E-12	8.62E-11	2.64
217999_s_at	*PHLDA1*	pleckstrin homology-like domain, family A, member 1	22822	5.92E-04	1.27E-03	2.64
238488_at	*hCG_2045899*	synleurin	353281	2.64E-06	9.89E-06	2.64
226028_at	*ROBO4*	roundabout homolog 4, magic roundabout (Drosophila)	54538	5.20E-06	1.81E-05	2.64
221710_x_at	*FAM176B / LOC100133999*	family with sequence similarity 176, member B / hypothetical protein LOC100133999	100133999 / 55194	2.41E-13	3.65E-12	2.64
236398_s_at				1.69E-10	1.44E-09	2.63
241017_at	*TBC1D8*	TBC1 domain family, member 8 (with GRAM domain)	11138	1.99E-08	1.14E-07	2.63
209648_x_at	*SOCS5*	suppressor of cytokine signaling 5	9655	6.69E-21	3.50E-19	2.63
224848_at	*CDK6*	cyclin-dependent kinase 6	1021	2.34E-10	1.93E-09	2.63
36711_at	*MAFF*	v-maf musculoaponeurotic fibrosarcoma oncogene homolog F (avian)	23764	1.02E-07	5.08E-07	2.63
203859_s_at	*PALM*	paralemmin	5064	2.40E-09	1.66E-08	2.62
241464_s_at				7.45E-05	1.99E-04	2.62
204540_at	*EEF1A2*	eukaryotic translation elongation factor 1 alpha 2	1917	1.43E-03	2.76E-03	2.62

Probe Set	Gene Symbol	Gene Title	Entrez Gene	*p*-value	q-value	FC
1564796_at	*EMP1*	epithelial membrane protein 1	2012	1.97E-04	4.75E-04	2.62
201538_s_at	*DUSP3*	dual specificity phosphatase 3	1845	4.00E-16	9.85E-15	2.62
227358_at	*ZBTB46*	zinc finger and BTB domain containing 46	140685	9.98E-08	4.99E-07	2.61
1552690_a_at	*CACNA2D4*	calcium channel, voltage-dependent, alpha 2/delta subunit 4	93589	7.68E-06	2.57E-05	2.61
204347_at	*AK3L1 / AK3L2*	adenylate kinase 3-like 1 / adenylate kinase 3-like 2	205 / 645619	6.36E-07	2.71E-06	2.61
225790_at	*MSRB3*	methionine sulfoxide reductase B3	253827	4.17E-13	6.07E-12	2.61
235439_at	*RBMS2*	RNA binding motif, single stranded interacting protein 2	5939	5.37E-07	2.32E-06	2.61
214457_at	*HOXA2*	homeobox A2	3199	3.16E-09	2.12E-08	2.61
210571_s_at	*CMAH*	cytidine monophosphate-N-acetylneuraminic acid hydroxylase (CMP-N-acetylneuraminate monooxygenase) pseudogene	8418	9.96E-11	8.90E-10	2.61
1552671_a_at	*SLC9A7*	solute carrier family 9 (sodium/hydrogen exchanger), member 7	84679	3.94E-11	3.84E-10	2.61
225590_at	*SH3RF1*	SH3 domain containing ring finger 1	57630	4.14E-05	1.17E-04	2.60
239123_at				1.90E-04	4.60E-04	2.60
230787_at				1.40E-18	5.09E-17	2.60
204745_x_at	*MT1G*	metallothionein 1G	4495	2.81E-06	1.05E-05	2.60
208767_s_at	*LAPTM4B*	lysosomal protein transmembrane 4 beta	55353	2.80E-05	8.22E-05	2.60
236893_at	*LOC100130740*	similar to hCG2042068	100130740	2.78E-08	1.55E-07	2.59
1556579_s_at	*IGSF10*	immunoglobulin superfamily, member 10	285313	1.48E-06	5.83E-06	2.59
216027_at	*TXNDC13*	thioredoxin domain containing 13	56255	4.26E-06	1.52E-05	2.59
214469_at	*HIST1H2AB / HIST1H2AE*	histone cluster 1, H2ab / histone cluster 1, H2ae	3012 / 8335	8.64E-05	2.27E-04	2.59
212803_at	*NAB2*	NGFI-A binding protein 2 (EGR1 binding protein 2)	4665	3.65E-09	2.43E-08	2.58
229507_at	*C3orf54*	chromosome 3 open reading frame 54	389119	2.58E-12	3.21E-11	2.58
223967_at	*ANGPTL6*	angiopoietin-like 6	83854	8.47E-10	6.33E-09	2.58
209911_x_at	*HIST1H2BD*	histone cluster 1, H2bd	3017	2.07E-09	1.45E-08	2.57
220193_at	*C1orf113*	chromosome 1 open reading frame 113	79729	4.89E-10	3.81E-09	2.57
217996_at	*PHLDA1*	pleckstrin homology-like domain, family A, member 1	22822	4.52E-04	9.98E-04	2.57
237108_x_at	*FLJ42875*	hypothetical LOC440556	440556	2.51E-08	1.41E-07	2.57
219051_x_at	*METRN*	meteorin, glial cell differentiation regulator	79006	9.08E-06	2.99E-05	2.57
204165_at	*WASF1*	WAS protein family, member 1	8936	7.56E-08	3.86E-07	2.57
228150_at	*SEC16B*	SEC16 homolog B (S. cerevisiae)	89866	2.77E-06	1.03E-05	2.57
228054_at	*TMEM44*	transmembrane protein 44	93109	4.65E-09	3.02E-08	2.56
244561_at				1.74E-15	3.81E-14	2.56
204083_s_at	*TPM2*	tropomyosin 2 (beta)	7169	1.17E-04	2.98E-04	2.56
217631_at	*GTPBP4 / IDI2*	GTP binding protein 4 / isopentenyl-diphosphate delta isomerase 2	23560 / 91734	2.35E-11	2.39E-10	2.56
225767_at	*LOC284801*	hypothetical protein LOC284801	284801	9.90E-04	1.99E-03	2.56
209661_at	*KIFC3*	kinesin family member C3	3801	2.36E-08	1.34E-07	2.55
1554020_at	*BICD1*	bicaudal D homolog 1 (Drosophila)	636	6.00E-10	4.59E-09	2.55
201819_at	*SCARB1*	scavenger receptor class B, member 1	949	4.82E-18	1.60E-16	2.55
212235_at	*PLXND1*	plexin D1	23129	1.12E-10	9.89E-10	2.55
40016_g_at	*LOC100128443 / MAST4*	hypothetical protein LOC100128443 / microtubule associated serine/threonine kinase family member 4	100128443 / 375449	6.26E-07	2.67E-06	2.55
228696_at	*SLC45A3*	solute carrier family 45, member 3	85414	1.94E-10	1.62E-09	2.55
232686_at	*SIGLECP3*	sialic acid binding Ig-like lectin, pseudogene 3	284367	4.01E-04	8.96E-04	2.54
220134_x_at	*FAM176B*	family with sequence similarity 176, member B	55194	7.97E-14	1.30E-12	2.54
227329_at	*ZBTB46*	zinc finger and BTB domain containing 46	140685	6.17E-09	3.93E-08	2.53
214682_at	*LOC399491*	LOC399491 protein	399491	1.89E-09	1.33E-08	2.53

Probe Set	Gene Symbol	Gene Title	Entrez Gene	*p*-value	q-value	FC
201329_s_at	*ETS2*	v-ets erythroblastosis virus E26 oncogene homolog 2 (avian)	2114	1.49E-12	1.95E-11	2.53
230218_at	*HIC1*	hypermethylated in cancer 1	3090	5.69E-05	1.56E-04	2.53
239915_at	*LOC100133311 / LOC100134764 / LOC285944*	similar to hCG1644697 / similar to hCG1644697 / hypothetical protein LOC285944	100133311 / 100134764 / 285944	1.59E-11	1.68E-10	2.53
203355_s_at	*PSD3*	pleckstrin and Sec7 domain containing 3	23362	1.89E-03	3.53E-03	2.53
208581_x_at	*MT1X*	metallothionein 1X	4501	1.26E-07	6.21E-07	2.53
214106_s_at	*GMDS*	GDP-mannose 4,6-dehydratase	2762	1.52E-09	1.09E-08	2.52
202932_at	*YES1*	v-yes-1 Yamaguchi sarcoma viral oncogene homolog 1	7525	1.86E-08	1.07E-07	2.52
204663_at	*ME3*	malic enzyme 3, NADP(+)-dependent, mitochondrial	10873	8.58E-08	4.33E-07	2.52
232201_at	*NKD2*	naked cuticle homolog 2 (Drosophila)	85409	6.53E-04	1.38E-03	2.52
223693_s_at	*RADIL*	Rap GTPase interactor	55698	4.23E-05	1.20E-04	2.52
235094_at				1.02E-04	2.64E-04	2.52
1554661_s_at	*C1orf71*	chromosome 1 open reading frame 71	163882	3.10E-15	6.45E-14	2.52
233328_x_at	*C20orf59*	chromosome 20 open reading frame 59	63910	2.76E-10	2.24E-09	2.52
1557814_a_at				7.99E-04	1.65E-03	2.51
210233_at	*IL1RAP*	interleukin 1 receptor accessory protein	3556	2.42E-07	1.12E-06	2.51
231982_at	*LOC284422*	similar to HSPC323	284422	2.70E-08	1.51E-07	2.51
219208_at	*FBXO11*	F-box protein 11	80204	6.74E-14	1.12E-12	2.51
208127_s_at	*SOCS5*	suppressor of cytokine signaling 5	9655	8.96E-22	5.21E-20	2.51
217988_at	*CCNB1IP1*	cyclin B1 interacting protein 1	57820	6.09E-20	2.71E-18	2.51
220494_s_at				6.78E-10	5.15E-09	2.51
206461_x_at	*MT1H*	metallothionein 1H	4496	1.29E-07	6.32E-07	2.50
1554621_at	*DGKE*	diacylglycerol kinase, epsilon 64kDa	8526	4.04E-11	3.93E-10	2.50
230570_at				6.05E-12	7.01E-11	2.50
204082_at	*PBX3*	pre-B-cell leukemia homeobox 3	5090	3.71E-08	2.01E-07	2.50
208937_s_at	*ID1*	inhibitor of DNA binding 1, dominant negative helix-loop-helix protein	3397	5.08E-05	1.41E-04	2.50
227708_at	*EEF1A1*	eukaryotic translation elongation factor 1 alpha 1	1915	4.35E-22	2.62E-20	2.50
214039_s_at	*LAPTM4B*	lysosomal protein transmembrane 4 beta	55353	4.35E-06	1.55E-05	2.50
238131_at	*PHC2*	polyhomeotic homolog 2 (Drosophila)	1912	2.78E-09	1.88E-08	2.50
241891_at				3.30E-12	4.01E-11	-2.50
238193_at				1.39E-19	5.75E-18	-2.50
215819_s_at	*RHCE / RHD*	Rh blood group, CcEe antigens / Rh blood group, D antigen	6006 / 6007	8.11E-08	4.12E-07	-2.51
238063_at	*TMEM154*	transmembrane protein 154	201799	9.05E-21	4.62E-19	-2.51
237442_at				4.71E-14	8.02E-13	-2.51
224975_at	*NFIA*	nuclear factor I/A	4774	2.10E-12	2.66E-11	-2.51
211005_at	*LAT / SPNS1*	linker for activation of T cells / spinster homolog 1 (Drosophila)	27040 / 83985	3.79E-14	6.56E-13	-2.51
222958_s_at	*DEPDC1*	DEP domain containing 1	55635	3.27E-16	8.19E-15	-2.51
228320_x_at	*CCDC64*	coiled-coil domain containing 64	92558	5.79E-13	8.24E-12	-2.51
233500_x_at	*CLEC2D*	C-type lectin domain family 2, member D	29121	4.01E-15	8.22E-14	-2.51
221724_s_at	*CLEC4A*	C-type lectin domain family 4, member A	50856	9.40E-15	1.81E-13	-2.52
221484_at	*B4GALT5*	UDP-Gal:betaGlcNAc beta 1,4-galactosyltransferase, polypeptide 5	9334	2.41E-28	3.13E-26	-2.52
231205_at				9.94E-16	2.28E-14	-2.52
209767_s_at	*GP1BB / SEPT5*	glycoprotein Ib (platelet), beta polypeptide / septin 5	2812 / 5413	7.92E-27	8.83E-25	-2.52
207220_at	*ART4*	ADP-ribosyltransferase 4 (Dombrock blood group)	420	9.22E-11	8.29E-10	-2.52
206283_s_at	*TAL1*	T-cell acute lymphocytic leukemia 1	6886	4.30E-15	8.76E-14	-2.52
211207_s_at	*ACSL6*	acyl-CoA synthetase long-chain family member 6	23305	4.67E-08	2.48E-07	-2.52

Probe Set	Gene Symbol	Gene Title	Entrez Gene	*p*-value	q-value	FC
228109_at	*RASGRF2*	CDNA clone IMAGE:5301750	5924	4.50E-17	1.28E-15	-2.53
1554892_a_at	*MS4A3*	membrane-spanning 4-domains, subfamily A, member 3 (hematopoietic cell-specific)	932	2.44E-19	9.80E-18	-2.53
220404_at	*GPR97*	G protein-coupled receptor 97	222487	1.35E-18	4.93E-17	-2.53
216333_x_at	*TNXA / TNXB*	tenascin XA pseudogene / tenascin XB	7146 / 7148	2.34E-07	1.09E-06	-2.53
236044_at	*PPAPDC1A*	phosphatidic acid phosphatase type 2 domain containing 1A	196051	6.07E-06	2.09E-05	-2.53
210430_x_at	*RHCE / RHD*	Rh blood group, CcEe antigens / Rh blood group, D antigen	6006 / 6007	7.52E-08	3.85E-07	-2.53
242405_at				1.69E-08	9.79E-08	-2.53
221011_s_at	*LBH*	limb bud and heart development homolog (mouse)	81606	8.39E-23	5.59E-21	-2.54
239205_s_at	*CR1 / CR1L*	complement component (3b/4b) receptor 1 (Knops blood group) / complement component (3b/4b) receptor 1-like	1378 / 1379	3.90E-09	2.57E-08	-2.54
231776_at	*EOMES*	eomesodermin homolog (Xenopus laevis)	8320	7.47E-11	6.85E-10	-2.54
206093_x_at	*TNXA / TNXB*	tenascin XA pseudogene / tenascin XB	7146 / 7148	4.69E-07	2.06E-06	-2.54
208601_s_at	*TUBB1*	tubulin, beta 1	81027	6.66E-14	1.10E-12	-2.55
222717_at	*SDPR*	serum deprivation response (phosphatidylserine binding protein)	8436	1.37E-13	2.16E-12	-2.55
210586_x_at	*RHD*	Rh blood group, D antigen	6007	1.52E-07	7.33E-07	-2.55
243819_at				1.55E-12	2.02E-11	-2.55
240027_at	*LIN7A*	lin-7 homolog A (C. elegans)	8825	1.71E-22	1.08E-20	-2.56
234764_x_at	*IGL@ / IGLV1-36 / IGLV1-44*	immunoglobulin lambda locus / immunoglobulin lambda variable 1-36 / immunoglobulin lambda variable 1-44	28823 / 28826 / 3535	7.98E-11	7.27E-10	-2.56
214032_at	*ZAP70*	zeta-chain (TCR) associated protein kinase 70kDa	7535	3.10E-08	1.71E-07	-2.56
211645_x_at				1.70E-11	1.78E-10	-2.56
228214_at				9.91E-14	1.59E-12	-2.56
240413_at	*PYHIN1*	pyrin and HIN domain family, member 1	149628	1.40E-08	8.27E-08	-2.56
215528_at				1.55E-15	3.43E-14	-2.56
1558827_a_at	*ZNF831*	zinc finger protein 831	128611	1.93E-15	4.15E-14	-2.57
216430_x_at	*IGL@*	immunoglobulin lambda locus	3535	4.00E-11	3.90E-10	-2.57
201906_s_at	*CTDSPL*	CTD (carboxy-terminal domain, RNA polymerase II, polypeptide A) small phosphatase-like	10217	6.74E-27	7.62E-25	-2.57
237340_at	*SLC26A8*	solute carrier family 26, member 8	116369	1.23E-17	3.82E-16	-2.57
210948_s_at	*LEF1*	lymphoid enhancer-binding factor 1	51176	1.16E-13	1.84E-12	-2.57
201393_s_at	*IGF2R*	insulin-like growth factor 2 receptor	3482	3.64E-20	1.68E-18	-2.58
215435_at				4.82E-18	1.60E-16	-2.58
227177_at	*CORO2A*	coronin, actin binding protein, 2A	7464	1.19E-27	1.46E-25	-2.58
216365_x_at	*IGL@ / IGLV3-19*	immunoglobulin lambda locus / immunoglobulin lambda variable 3-19	28797 / 3535	1.94E-13	3.00E-12	-2.58
210429_at	*RHCE / RHD*	Rh blood group, CcEe antigens / Rh blood group, D antigen	6006 / 6007	6.62E-07	2.81E-06	-2.58
210254_at	*MS4A3*	membrane-spanning 4-domains, subfamily A, member 3 (hematopoietic cell-specific)	932	1.67E-27	1.98E-25	-2.58
221602_s_at	*FAIM3*	Fas apoptotic inhibitory molecule 3	9214	9.95E-08	4.98E-07	-2.59
204714_s_at	*F5*	coagulation factor V (proaccelerin, labile factor)	2153	4.89E-13	7.05E-12	-2.59
240154_at				9.53E-16	2.19E-14	-2.59
210367_s_at	*PTGES*	prostaglandin E synthase	9536	7.17E-07	3.02E-06	-2.60
242055_at				1.24E-08	7.41E-08	-2.60
212372_at	*MYH10*	myosin, heavy chain 10, non-muscle	4628	6.73E-12	7.73E-11	-2.60
216491_x_at	*IGHM*	immunoglobulin heavy constant mu	3507	1.04E-10	9.29E-10	-2.60
207063_at	*CYorf14*	chromosome Y open reading frame 14	55410	3.31E-13	4.91E-12	-2.60
230559_x_at	*FGD4*	FYVE, RhoGEF and PH domain containing 4	121512	1.31E-22	8.47E-21	-2.60
1557632_at				1.84E-15	3.99E-14	-2.60

Probe Set	Gene Symbol	Gene Title	Entrez Gene	*p*-value	q-value	FC
241542_at				2.44E-08	1.37E-07	-2.60
217763_s_at	*RAB31*	RAB31, member RAS oncogene family	11031	1.97E-25	1.81E-23	-2.61
213288_at	*MBOAT2*	membrane bound O-acyltransferase domain containing 2	129642	2.71E-22	1.68E-20	-2.61
224833_at	*ETS1*	v-ets erythroblastosis virus E26 oncogene homolog 1 (avian)	2113	4.40E-18	1.48E-16	-2.61
229894_s_at	*RAB43*	RAB43, member RAS oncogene family	339122	1.02E-15	2.32E-14	-2.61
209238_at	*STX3*	syntaxin 3	6809	3.92E-35	1.01E-32	-2.61
219892_at	*TM6SF1*	transmembrane 6 superfamily member 1	53346	1.15E-24	9.55E-23	-2.61
229298_at	*KBTBD7*	kelch repeat and BTB (POZ) domain containing 7	84078	4.05E-31	6.88E-29	-2.61
236442_at	*DPF3*	D4, zinc and double PHD fingers, family 3	8110	2.91E-13	4.34E-12	-2.61
240178_at				3.72E-15	7.68E-14	-2.62
206765_at	*KCNJ2*	potassium inwardly-rectifying channel, subfamily J, member 2	3759	1.76E-07	8.41E-07	-2.62
224404_s_at	*FCRL5*	Fc receptor-like 5	83416	5.79E-13	8.24E-12	-2.62
237625_s_at				7.73E-15	1.50E-13	-2.62
227004_at				3.52E-31	6.03E-29	-2.63
209473_at	*ENTPD1*	ectonucleoside triphosphate diphosphohydrolase 1	953	1.50E-22	9.51E-21	-2.63
205844_at	*VNN1*	vanin 1	8876	3.79E-09	2.51E-08	-2.63
213241_at	*PLXNC1*	plexin C1	10154	4.30E-22	2.60E-20	-2.63
231484_at				4.13E-13	6.01E-12	-2.63
208364_at	*INPP4A*	inositol polyphosphate-4-phosphatase, type I, 107kDa	3631	5.95E-13	8.44E-12	-2.63
205315_s_at	*SNTB2*	syntrophin, beta 2 (dystrophin-associated protein A1, 59kDa, basic component 2)	6645	4.84E-33	9.35E-31	-2.63
207341_at	*PRTN3*	proteinase 3	5657	4.91E-21	2.61E-19	-2.63
227717_at	*FLJ41603*	FLJ41603 protein	389337	8.02E-18	2.57E-16	-2.63
209348_s_at	*MAF*	v-maf musculoaponeurotic fibrosarcoma oncogene homolog (avian)	4094	2.55E-12	3.17E-11	-2.64
212538_at	*DOCK9*	dedicator of cytokinesis 9	23348	3.89E-12	4.67E-11	-2.64
208052_x_at	*CEACAM3*	carcinoembryonic antigen-related cell adhesion molecule 3	1084	6.12E-27	6.98E-25	-2.64
204445_s_at	*ALOX5*	arachidonate 5-lipoxygenase	240	7.17E-15	1.41E-13	-2.64
230240_at				1.25E-14	2.35E-13	-2.64
209864_at	*FRAT2*	frequently rearranged in advanced T-cell lymphomas 2	23401	7.96E-28	9.92E-26	-2.65
219947_at	*CLEC4A*	C-type lectin domain family 4, member A	50856	4.16E-16	1.02E-14	-2.65
205786_s_at	*ITGAM*	integrin, alpha M (complement component 3 receptor 3 subunit)	3684	2.48E-33	5.01E-31	-2.65
212830_at	*MEGF9*	multiple EGF-like-domains 9	1955	9.53E-16	2.19E-14	-2.65
1556331_a_at				4.59E-11	4.42E-10	-2.65
208079_s_at	*AURKA*	aurora kinase A	6790	5.45E-20	2.45E-18	-2.66
244414_at				2.37E-08	1.34E-07	-2.66
217227_x_at	*IGL@*	immunoglobulin lambda locus	3535	1.64E-12	2.13E-11	-2.66
236280_at				2.02E-10	1.68E-09	-2.66
221601_s_at	*FAIM3*	Fas apoptotic inhibitory molecule 3	9214	4.87E-12	5.74E-11	-2.66
239108_at	*FAR2*	Fatty acyl CoA reductase 2, mRNA (cDNA clone MGC:22328 IMAGE:4732586)	55711	5.95E-20	2.66E-18	-2.66
1553514_a_at	*VNN3*	vanin 3	55350	1.02E-15	2.32E-14	-2.66
239492_at	*SEC14L4*	SEC14-like 4 (S. cerevisiae)	284904	2.80E-13	4.19E-12	-2.66
239808_at				3.04E-12	3.72E-11	-2.66
1568593_a_at	*NUDT16P*	nudix (nucleoside diphosphate linked moiety X)-type motif 16 pseudogene	152195	1.06E-24	8.84E-23	-2.66
226207_at	*RILPL1*	Rab interacting lysosomal protein-like 1	353116	3.94E-23	2.75E-21	-2.66
205557_at	*BPI*	bactericidal/permeability-increasing protein	671	1.22E-23	9.02E-22	-2.66

Probe Set	Gene Symbol	Gene Title	Entrez Gene	*p*-value	q-value	FC
207854_at	*GYPE*	glycophorin E	2996	4.93E-10	3.84E-09	-2.67
217235_x_at	*IGL@ / IGLC1 / IGLV2-11 / IGLV2-18 / IGLV2-23*	immunoglobulin lambda locus / immunoglobulin lambda constant 1 (Mcg marker) / immunoglobulin lambda variable 2-11 / immunoglobulin lambda variable 2-18 / immunoglobulin lambda variable 2-23	28813 / 28814 / 28816 / 3535 / 3537	2.34E-08	1.33E-07	-2.68
240265_at	*LOC100133233 / TRAF3IP3*	hypothetical protein LOC100133233 / TRAF3 interacting protein 3	100133233 / 80342	1.54E-11	1.64E-10	-2.68
212823_s_at	*PLEKHG3*	pleckstrin homology domain containing, family G (with RhoGef domain) member 3	26030	3.90E-25	3.46E-23	-2.68
204174_at	*ALOX5AP*	arachidonate 5-lipoxygenase-activating protein	241	7.76E-36	2.12E-33	-2.68
1555659_a_at	*TREML1*	triggering receptor expressed on myeloid cells-like 1	340205	8.17E-12	9.23E-11	-2.68
243099_at	*NFAM1*	NFAT activating protein with ITAM motif 1	150372	4.21E-30	6.52E-28	-2.68
221345_at	*FFAR2*	free fatty acid receptor 2	2867	3.73E-09	2.47E-08	-2.68
238532_at	*DPF3*	D4, zinc and double PHD fingers, family 3	8110	7.33E-16	1.72E-14	-2.69
1556761_at				2.99E-14	5.25E-13	-2.70
211650_x_at	*IGHA1 / IGHD / IGHG1 / IGHG3 / IGHM / IGHV4-31 / IGHV@ / LOC100126583*	immunoglobulin heavy constant alpha 1 / immunoglobulin heavy constant delta / immunoglobulin heavy constant gamma 1 (G1m marker) / immunoglobulin heavy constant gamma 3 (G3m marker) / immunoglobulin heavy constant mu / immunoglobulin heavy variable 4-31 / immunoglobulin heavy variable group / hypothetical LOC100126583	100126583 / 28396 / 3493 / 3495 / 3500 / 3502 / 3507 / 3509	7.00E-12	8.01E-11	-2.70
227233_at	*TSPAN2*	tetraspanin 2	10100	1.23E-12	1.64E-11	-2.70
213539_at	*CD3D*	CD3d molecule, delta (CD3-TCR complex)	915	1.13E-09	8.28E-09	-2.70
215621_s_at	*IGHD*	immunoglobulin heavy constant delta	3495	5.32E-07	2.30E-06	-2.70
239142_at	*RFESD*	Rieske (Fe-S) domain containing	317671	3.10E-13	4.61E-12	-2.71
205118_at	*FPR1*	formyl peptide receptor 1	2357	4.34E-08	2.32E-07	-2.71
217762_s_at	*RAB31*	RAB31, member RAS oncogene family	11031	1.46E-25	1.36E-23	-2.71
230802_at	*ARHGAP24*	Rho GTPase activating protein 24	83478	1.76E-16	4.66E-15	-2.71
216576_x_at	*IGKC / IGKV1-5 / LOC647506 / LOC652694*	immunoglobulin kappa constant / immunoglobulin kappa variable 1-5 / similar to Ig kappa chain V-I region HK101 precursor / similar to Ig kappa chain V-I region HK102 precursor	28299 / 3514 / 647506 / 652694	2.41E-12	3.02E-11	-2.71
217281_x_at	*IGH@ / IGHA1 / IGHA2 / IGHG1 / IGHG2 / IGHG3 / IGHM / IGHV4-31 / LOC100126583 / LOC100133739 / LOC652494*	immunoglobulin heavy locus / immunoglobulin heavy constant alpha 1 / immunoglobulin heavy constant alpha 2 (A2m marker) / immunoglobulin heavy constant gamma 1 (G1m marker) / immunoglobulin heavy constant gamma 2 (G2m marker) / immunoglobulin heavy constant gamma 3 (G3m marker) / immunoglobulin heavy constant mu / immunoglobulin heavy variable 4-31 / hypothetical LOC100126583 / similar to hCG2038920 / similar to Ig heavy chain V-III region VH26 precursor	100126583 / 100133739 / 28396 / 3492 / 3493 / 3494 / 3500 / 3501 / 3502 / 3507 / 652494	2.78E-11	2.78E-10	-2.72
211634_x_at	*IGHM / LOC100133862*	immunoglobulin heavy constant mu / similar to hCG1773549	100133862 / 3507	2.00E-08	1.15E-07	-2.72
206366_x_at	*XCL1*	chemokine (C motif) ligand 1	6375	1.35E-12	1.78E-11	-2.72
219737_s_at	*PCDH9*	protocadherin 9	5101	2.51E-08	1.41E-07	-2.72
223944_at	*NLRP12*	NLR family, pyrin domain containing 12	91662	1.91E-17	5.76E-16	-2.72
235567_at	*RORA*	Hypothetical protein LOC283666, mRNA (cDNA clone IMAGE:4750925)	6095	4.94E-08	2.62E-07	-2.72
219396_s_at	*NEIL1*	nei endonuclease VIII-like 1 (E. coli)	79661	4.42E-25	3.85E-23	-2.72
211339_s_at	*ITK*	IL2-inducible T-cell kinase	3702	3.16E-12	3.86E-11	-2.72
220694_at	*DDEF1IT1*	DDEF1 intronic transcript 1 (non-protein coding)	29065	3.17E-09	2.13E-08	-2.72
214945_at	*FAM153A / FAM153B / FAM153C*	family with sequence similarity 153, member A / family with sequence similarity 153, member B / family with sequence similarity 153, member C	202134 / 285596 / 653316	5.39E-19	2.06E-17	-2.72

Probe Set	Gene Symbol	Gene Title	Entrez Gene	*p*-value	q-value	FC
211644_x_at	*IGK@ / IGKC / IGKV3-20 / IGKV3D-11 / IGKV3D-15 / LOC440871*	immunoglobulin kappa locus / immunoglobulin kappa constant / immunoglobulin kappa variable 3-20 / immunoglobulin kappa variable 3D-11 / immunoglobulin kappa variable 3D-15 (gene/pseudogene) / similar to hCG2043206	28875 / 28876 / 28912 / 3514 / 440871 / 50802	5.13E-11	4.88E-10	-2.73
227747_at	*MPZL3*	myelin protein zero-like 3	196264	3.63E-16	9.05E-15	-2.73
209765_at	*ADAM19*	ADAM metallopeptidase domain 19 (meltrin beta)	8728	9.01E-30	1.33E-27	-2.73
1560706_at				6.20E-25	5.30E-23	-2.73
218963_s_at	*KRT23*	keratin 23 (histone deacetylase inducible)	25984	1.13E-03	2.24E-03	-2.73
1560259_at				9.08E-13	1.24E-11	-2.73
202391_at	*BASP1*	brain abundant, membrane attached signal protein 1	10409	7.05E-30	1.06E-27	-2.73
207892_at	*CD40LG*	CD40 ligand	959	2.54E-23	1.79E-21	-2.73
234644_x_at				3.41E-17	9.96E-16	-2.73
225061_at	*DNAJA4*	DnaJ (Hsp40) homolog, subfamily A, member 4	55466	1.67E-16	4.41E-15	-2.73
206937_at	*SPTA1*	spectrin, alpha, erythrocytic 1 (elliptocytosis 2)	6708	3.28E-11	3.25E-10	-2.74
206245_s_at	*IVNS1ABP*	influenza virus NS1A binding protein	10625	4.42E-30	6.79E-28	-2.74
234986_at				1.02E-16	2.79E-15	-2.74
1553542_at	*CCDC125*	coiled-coil domain containing 125	202243	1.60E-35	4.27E-33	-2.75
221485_at	*B4GALT5*	UDP-Gal:betaGlcNAc beta 1,4- galactosyltransferase, polypeptide 5	9334	1.29E-39	5.36E-37	-2.75
211796_s_at	*TRBC1 / TRBC2*	T cell receptor beta constant 1 / T cell receptor beta constant 2	28638 / 28639	8.83E-13	1.21E-11	-2.76
214084_x_at	*LOC648998*	similar to Neutrophil cytosol factor 1 (NCF-1) (Neutrophil NADPH oxidase factor 1) (47 kDa neutrophil oxidase factor) (p47-phox) (NCF-47K) (47 kDa autosomal chronic granulomatous disease protein) (NOXO2)	648998	5.70E-23	3.90E-21	-2.76
219890_at	*CLEC5A*	C-type lectin domain family 5, member A	23601	7.18E-19	2.69E-17	-2.76
217378_x_at	*LOC100130100*	similar to hCG26659	100130100	7.73E-10	5.82E-09	-2.76
205728_at				5.06E-17	1.43E-15	-2.76
1557685_at	*C4orf38*	CDNA FLJ30278 fis, clone BRACE2002755	152641	2.68E-24	2.16E-22	-2.76
214768_x_at	*FAM20B*	(clone TR1.6VL) anti-thyroid peroxidase monoclonal autoantibody IgK chain, V region	9917	5.30E-15	1.06E-13	-2.76
221778_at	*JHDM1D*	jumonji C domain containing histone demethylase 1 homolog D (S. cerevisiae)	80853	4.98E-35	1.27E-32	-2.77
236583_at				4.63E-11	4.45E-10	-2.77
226338_at	*TMEM55A*	transmembrane protein 55A	55529	1.17E-36	3.75E-34	-2.77
239629_at	*CFLAR*	I-FLICE isoform 5	8837	3.36E-16	8.40E-15	-2.77
241060_x_at				2.73E-11	2.74E-10	-2.78
230803_s_at	*ARHGAP24*	Rho GTPase activating protein 24	83478	3.19E-20	1.50E-18	-2.78
210724_at	*EMR3*	egf-like module containing, mucin-like, hormone receptor-like 3	84658	2.17E-10	1.80E-09	-2.78
203485_at	*RTN1*	reticulon 1	6252	7.44E-06	2.50E-05	-2.79
225792_at	*HOOK1*	hook homolog 1 (Drosophila)	51361	4.67E-16	1.14E-14	-2.79
206390_x_at	*PF4*	platelet factor 4	5196	6.55E-15	1.29E-13	-2.79
214567_s_at	*XCL1 / XCL2*	chemokine (C motif) ligand 1 / chemokine (C motif) ligand 2	6375 / 6846	2.99E-12	3.66E-11	-2.79
239585_at	*KAT2B*	P300/CBP-associated factor (P/CAF)	8850	5.41E-24	4.17E-22	-2.79
207269_at	*DEFA4*	defensin, alpha 4, corticostatin	1669	5.17E-31	8.62E-29	-2.79
229952_at				3.79E-13	5.54E-12	-2.80
243546_at				4.94E-10	3.84E-09	-2.80
222285_at	*IGHD*	immunoglobulin heavy constant delta	3495	3.97E-21	2.13E-19	-2.80
239327_at				7.27E-13	1.01E-11	-2.80
244087_at				3.97E-13	5.78E-12	-2.80
234317_s_at	*STOX2*	storkhead box 2	56977	5.95E-19	2.25E-17	-2.80

Probe Set	Gene Symbol	Gene Title	Entrez Gene	*p*-value	q-value	FC
227232_at	*EVL*	Enah/Vasp-like	51466	7.38E-09	4.62E-08	-2.81
206109_at	*FUT1*	fucosyltransferase 1 (galactoside 2-alpha-L-fucosyltransferase, H blood group)	2523	9.95E-17	2.72E-15	-2.81
203827_at	*WIPI1*	WD repeat domain, phosphoinositide interacting 1	55062	7.08E-35	1.73E-32	-2.81
237207_at				1.79E-11	1.87E-10	-2.81
241893_at				2.09E-15	4.45E-14	-2.81
1552632_a_at	*ARSG*	arylsulfatase G	22901	3.95E-39	1.54E-36	-2.81
232330_at	*C7orf44*	CDNA: FLJ21208 fis, clone COL00363	55744	3.34E-24	2.65E-22	-2.81
237033_at	*FAM159A*	family with sequence similarity 159, member A	348378	3.05E-20	1.43E-18	-2.82
237104_at				7.66E-15	1.49E-13	-2.82
215949_x_at	*IGHM / LOC652494*	immunoglobulin heavy constant mu / similar to Ig heavy chain V-III region VH26 precursor	3507 / 652494	1.85E-10	1.56E-09	-2.82
217764_s_at	*RAB31*	RAB31, member RAS oncogene family	11031	1.08E-32	2.05E-30	-2.82
213193_x_at	*TRBC1*	T cell receptor beta constant 1	28639	5.47E-16	1.32E-14	-2.82
214366_s_at	*ALOX5*	arachidonate 5-lipoxygenase	240	2.88E-16	7.30E-15	-2.82
1556764_s_at				1.41E-12	1.85E-11	-2.83
207935_s_at	*KRT13*	keratin 13	3860	3.98E-14	6.86E-13	-2.83
206618_at	*IL18R1*	interleukin 18 receptor 1	8809	8.61E-18	2.75E-16	-2.83
209276_s_at	*GLRX*	glutaredoxin (thioltransferase)	2745	0.00E+00	0.00E+00	-2.83
222892_s_at	*TMEM40*	transmembrane protein 40	55287	7.19E-16	1.70E-14	-2.83
204777_s_at	*MAL*	mal, T-cell differentiation protein	4118	4.19E-12	4.99E-11	-2.84
227497_at				2.27E-13	3.46E-12	-2.84
230509_at	*SNX22*	sorting nexin 22	79856	5.09E-23	3.50E-21	-2.84
230748_at	*SLC16A6*	solute carrier family 16, member 6 (monocarboxylic acid transporter 7)	9120	1.61E-14	2.97E-13	-2.84
1555355_a_at	*ETS1*	v-ets erythroblastosis virus E26 oncogene homolog 1 (avian)	2113	3.65E-20	1.68E-18	-2.84
229725_at	*ACSL6*	Acyl-CoA synthetase long-chain family member 6, mRNA (cDNA clone MGC:26420 IMAGE:4830357)	23305	3.02E-10	2.44E-09	-2.84
211649_x_at	*IGH@ / IGHA1 / IGHA2 / IGHG1 / IGHM / LOC642131*	immunoglobulin heavy locus / immunoglobulin heavy constant alpha 1 / immunoglobulin heavy constant alpha 2 (A2m marker) / immunoglobulin heavy constant gamma 1 (G1m marker) / immunoglobulin heavy constant mu / similar to hCG1812074	3492 / 3493 / 3494 / 3500 / 3507 / 642131	2.49E-11	2.52E-10	-2.84
216984_x_at	*IGLV2-11 / IGLV2-18 / IGLV2-23*	immunoglobulin lambda variable 2-11 / immunoglobulin lambda variable 2-18 / immunoglobulin lambda variable 2-23	28813 / 28814 / 28816	1.71E-11	1.79E-10	-2.85
227929_at				3.74E-20	1.72E-18	-2.85
201506_at	*TGFBI*	transforming growth factor, beta-induced, 68kDa	7045	5.68E-11	5.36E-10	-2.86
229934_at				2.12E-14	3.84E-13	-2.86
221030_s_at	*ARHGAP24*	Rho GTPase activating protein 24	83478	2.59E-29	3.68E-27	-2.86
201131_s_at	*CDH1*	cadherin 1, type 1, E-cadherin (epithelial)	999	1.72E-11	1.80E-10	-2.86
221627_at	*TRIM10*	tripartite motif-containing 10	10107	2.90E-12	3.56E-11	-2.86
1566518_at				2.08E-16	5.41E-15	-2.86
224970_at	*NFIA*	nuclear factor I/A	4774	4.33E-14	7.41E-13	-2.87
226726_at	*MBOAT2*	membrane bound O-acyltransferase domain containing 2	129642	1.85E-27	2.17E-25	-2.87
223836_at	*FGFBP2*	fibroblast growth factor binding protein 2	83888	6.39E-07	2.72E-06	-2.87
202119_s_at	*CPNE3*	copine III	8895	5.39E-35	1.36E-32	-2.87
207926_at	*GP5*	glycoprotein V (platelet)	2814	2.97E-18	1.03E-16	-2.87
220945_x_at	*MANSC1*	MANSC domain containing 1	54682	7.84E-26	7.59E-24	-2.88
1553169_at	*LRRN4*	leucine rich repeat neuronal 4	164312	1.88E-15	4.07E-14	-2.88
213689_x_at	*FAM69A*	family with sequence similarity 69, member A	388650	3.76E-15	7.76E-14	-2.88

Probe Set	Gene Symbol	Gene Title	Entrez Gene	*p*-value	q-value	FC
219471_at	*C13orf18 / LOC728970*	chromosome 13 open reading frame 18 / hypothetical LOC728970	728970 / 80183	7.07E-20	3.08E-18	-2.88
227867_at	*LOC129293*	hypothetical protein LOC129293	129293	6.40E-16	1.52E-14	-2.88
204961_s_at	*LOC648998 / NCF1 / NCF1B / NCF1C*	similar to Neutrophil cytosol factor 1 (NCF-1) (Neutrophil NADPH oxidase factor 1) (47 kDa neutrophil oxidase factor) (p47-phox) (NCF-47K) (47 kDa autosomal chronic granulomatous disease protein) (NOXO2) / neutrophil cytosolic factor 1 / neutrophil cytosolic factor 1B pseudogene / neutrophil cytosolic factor 1C pseudogene	648998 / 653361 / 654816 / 654817	8.53E-22	4.99E-20	-2.88
233393_at				7.92E-16	1.85E-14	-2.88
206337_at	*CCR7*	chemokine (C-C motif) receptor 7	1236	5.40E-10	4.17E-09	-2.89
237119_at				4.06E-14	6.98E-13	-2.89
228067_at	*C2orf55*	chromosome 2 open reading frame 55	343990	9.71E-17	2.66E-15	-2.90
229970_at	*KBTBD7*	kelch repeat and BTB (POZ) domain containing 7	84078	4.08E-25	3.60E-23	-2.90
228031_at	*TTPAL*	tocopherol (alpha) transfer protein-like	79183	0.00E+00	0.00E+00	-2.90
241692_at				3.02E-17	8.88E-16	-2.90
231616_at				2.75E-07	1.26E-06	-2.91
240440_at	*NPL*	CDNA FLJ51988 complete cds, highly similar to Homo sapiens N-acetylneuraminate pyruvate lyase (dihydrodipicolinate synthase) (NPL), mRNA	80896	2.32E-19	9.33E-18	-2.92
1553706_at	*HTRA4*	HtrA serine peptidase 4	203100	3.60E-19	1.40E-17	-2.93
215449_at	*BZRPL1*	benzodiazapine receptor (peripheral)-like 1	222642	1.62E-10	1.38E-09	-2.93
211583_x_at	*NCR3*	natural cytotoxicity triggering receptor 3	259197	4.69E-15	9.48E-14	-2.93
205262_at	*KCNH2*	potassium voltage-gated channel, subfamily H (eag-related), member 2	3757	1.97E-17	5.91E-16	-2.94
234196_at				3.45E-17	1.01E-15	-2.94
206025_s_at	*TNFAIP6*	tumor necrosis factor, alpha-induced protein 6	7130	1.46E-09	1.05E-08	-2.94
206023_at	*NMU*	neuromedin U	10874	1.98E-06	7.59E-06	-2.94
205936_s_at	*HK3*	hexokinase 3 (white cell)	3101	7.28E-21	3.77E-19	-2.94
218805_at	*GIMAP5*	GTPase, IMAP family member 5	55340	5.00E-11	4.77E-10	-2.95
1556762_a_at				8.73E-14	1.42E-12	-2.95
205922_at	*VNN2*	vanin 2	8875	1.07E-11	1.17E-10	-2.95
210451_at	*PKLR*	pyruvate kinase, liver and RBC	5313	8.17E-13	1.13E-11	-2.96
236045_x_at				2.13E-14	3.85E-13	-2.96
216829_at	*IGK@ / IGKC / IGKV1-5 / LOC647506 / LOC652694*	immunoglobulin kappa locus / immunoglobulin kappa constant / immunoglobulin kappa variable 1-5 / similar to Ig kappa chain V-I region HK101 precursor / similar to Ig kappa chain V-I region HK102 precursor	28299 / 3514 / 50802 / 647506 / 652694	1.48E-10	1.27E-09	-2.96
205110_s_at	*FGF13*	fibroblast growth factor 13	2258	2.36E-09	1.63E-08	-2.96
243178_at				4.43E-18	1.49E-16	-2.97
211643_x_at	*IGK@ / IGKV3-20 / IGKV3D-11 / IGKV3D-15 / LOC440871*	immunoglobulin kappa locus / immunoglobulin kappa variable 3-20 / immunoglobulin kappa variable 3D-11 / immunoglobulin kappa variable 3D-15 (gene/pseudogene) / similar to hCG2043206	28875 / 28876 / 28912 / 440871 / 50802	3.11E-07	1.41E-06	-2.97
230464_at	*S1PR5*	sphingosine-1-phosphate receptor 5	53637	4.45E-07	1.96E-06	-2.98
207460_at	*GZMM*	granzyme M (lymphocyte met-ase 1)	3004	1.75E-11	1.83E-10	-2.98
206440_at	*LIN7A*	lin-7 homolog A (C. elegans)	8825	1.08E-21	6.18E-20	-2.99
239946_at				6.91E-19	2.59E-17	-3.00
213674_x_at	*IGHD*	immunoglobulin heavy constant delta	3495	1.44E-09	1.04E-08	-3.01
228585_at	*ENTPD1*	ectonucleoside triphosphate diphosphohydrolase 1	953	3.36E-26	3.44E-24	-3.02
226682_at	*RORA*	RAR-related orphan receptor A	6095	1.20E-10	1.05E-09	-3.02
232958_at				3.59E-24	2.84E-22	-3.02
220005_at	*P2RY13*	purinergic receptor P2Y, G-protein coupled, 13	53829	1.07E-21	6.18E-20	-3.03

Probe Set	Gene Symbol	Gene Title	Entrez Gene	*p*-value	q-value	FC
1558549_s_at	*VNN1*	vanin 1	8876	3.76E-09	2.49E-08	-3.03
1559131_a_at				1.08E-21	6.21E-20	-3.03
213836_s_at	*WIPI1*	WD repeat domain, phosphoinositide interacting 1	55062	1.40E-45	9.72E-43	-3.03
216012_at				1.36E-17	4.19E-16	-3.04
204581_at	*CD22*	CD22 molecule	933	3.07E-14	5.38E-13	-3.04
206150_at	*CD27*	CD27 molecule	939	6.38E-20	2.82E-18	-3.04
219528_s_at	*BCL11B*	B-cell CLL/lymphoma 11B (zinc finger protein)	64919	5.08E-13	7.30E-12	-3.04
207339_s_at	*LTB*	lymphotoxin beta (TNF superfamily, member 3)	4050	1.81E-18	6.49E-17	-3.05
229029_at				1.86E-17	5.63E-16	-3.05
205403_at	*IL1R2*	interleukin 1 receptor, type II	7850	3.33E-07	1.51E-06	-3.05
227948_at	*FGD4*	FYVE, RhoGEF and PH domain containing 4	121512	2.14E-25	1.94E-23	-3.06
219159_s_at	*SLAMF7*	SLAM family member 7	57823	6.32E-13	8.93E-12	-3.06
210915_x_at	*TRBC1*	T cell receptor beta constant 1	28639	4.32E-17	1.24E-15	-3.07
220615_s_at	*FAR2*	fatty acyl CoA reductase 2	55711	2.36E-26	2.50E-24	-3.07
230012_at	*C17orf44*	chromosome 17 open reading frame 44	284029	6.14E-22	3.66E-20	-3.09
240019_at				2.93E-21	1.60E-19	-3.09
241652_x_at				3.87E-28	4.99E-26	-3.09
203923_s_at	*CYBB*	cytochrome b-245, beta polypeptide	1536	4.08E-28	5.24E-26	-3.10
229967_at	*CMTM2*	CKLF-like MARVEL transmembrane domain containing 2	146225	4.21E-12	5.02E-11	-3.10
1555691_a_at	*KLRC4 / KLRK1*	killer cell lectin-like receptor subfamily C, member 4 / killer cell lectin-like receptor subfamily K, member 1	22914 / 8302	3.84E-15	7.91E-14	-3.10
231133_at	*C2orf39*	chromosome 2 open reading frame 39	92749	4.46E-31	7.48E-29	-3.11
207651_at	*GPR171*	G protein-coupled receptor 171	29909	1.28E-06	5.14E-06	-3.11
206881_s_at	*LILRA3*	leukocyte immunoglobulin-like receptor, subfamily A (without TM domain), member 3	11026	2.31E-08	1.31E-07	-3.11
241871_at	*CAMK4*	calcium/calmodulin-dependent protein kinase IV	814	1.92E-15	4.15E-14	-3.11
227354_at	*PAG1*	phosphoprotein associated with glycosphingolipid microdomains 1	55824	1.10E-32	2.09E-30	-3.12
230983_at	*FAM129C*	family with sequence similarity 129, member C	199786	1.53E-10	1.31E-09	-3.12
235651_at				3.47E-17	1.01E-15	-3.13
1560034_a_at				1.12E-19	4.67E-18	-3.13
226047_at	*MRVI1*	murine retrovirus integration site 1 homolog	10335	8.61E-25	7.27E-23	-3.13
202018_s_at	*LTF*	lactotransferrin	4057	1.53E-34	3.53E-32	-3.13
208022_s_at	*CDC14B*	CDC14 cell division cycle 14 homolog B (S. cerevisiae)	8555	1.18E-28	1.55E-26	-3.13
206641_at	*TNFRSF17*	tumor necrosis factor receptor superfamily, member 17	608	1.11E-08	6.71E-08	-3.13
204351_at	*S100P*	S100 calcium binding protein P	6286	5.29E-37	1.74E-34	-3.13
239196_at	*ANKRD22*	ankyrin repeat domain 22	118932	2.46E-39	1.00E-36	-3.14
227396_at	*PTPRJ*	protein tyrosine phosphatase, receptor type, J	5795	1.44E-28	1.88E-26	-3.14
202118_s_at	*CPNE3*	copine III	8895	4.54E-36	1.37E-33	-3.15
231688_at	*MMP8*	matrix metallopeptidase 8 (neutrophil collagenase)	4317	1.20E-30	1.94E-28	-3.15
239913_at	*SLC10A4*	solute carrier family 10 (sodium/bile acid cotransporter family), member 4	201780	2.33E-10	1.92E-09	-3.16
228071_at	*GIMAP7*	GTPase, IMAP family member 7	168537	1.93E-12	2.48E-11	-3.17
241155_at				3.73E-09	2.47E-08	-3.17
206026_s_at	*TNFAIP6*	tumor necrosis factor, alpha-induced protein 6	7130	2.67E-10	2.18E-09	-3.19
228298_at	*FAM113B*	family with sequence similarity 113, member B	91523	2.38E-17	7.05E-16	-3.19
204890_s_at	*LCK*	lymphocyte-specific protein tyrosine kinase	3932	2.92E-11	2.91E-10	-3.19
203413_at	*NELL2*	NEL-like 2 (chicken)	4753	1.27E-15	2.87E-14	-3.19
235372_at	*FCRLA*	Fc receptor-like A	84824	3.24E-22	1.99E-20	-3.19

Probe Set	Gene Symbol	Gene Title	Entrez Gene	*p*-value	q-value	FC
224832_at	*DUSP16*	dual specificity phosphatase 16	80824	2.00E-20	9.67E-19	-3.19
237194_at				4.43E-15	8.98E-14	-3.21
236305_at	*RFESD*	Rieske (Fe-S) domain containing	317671	3.37E-17	9.84E-16	-3.21
226039_at	*MGAT4A*	mannosyl (alpha-1,3-)-glycoprotein beta-1,4-N-acetylglucosaminyltransferase, isozyme A	11320	2.98E-18	1.04E-16	-3.21
206255_at	*BLK*	B lymphoid tyrosine kinase	640	1.51E-17	4.61E-16	-3.22
224412_s_at	*TRPM6*	transient receptor potential cation channel, subfamily M, member 6	140803	3.35E-09	2.24E-08	-3.22
219998_at	*HSPC159*	galectin-related protein	29094	2.53E-23	1.79E-21	-3.23
222896_at	*TMEM38A*	transmembrane protein 38A	79041	2.08E-18	7.40E-17	-3.23
211372_s_at	*IL1R2*	interleukin 1 receptor, type II	7850	6.07E-05	1.65E-04	-3.24
214777_at	*IGKV4-1*	immunoglobulin kappa variable 4-1	28908	1.58E-14	2.92E-13	-3.25
1562529_s_at				1.20E-11	1.30E-10	-3.26
1559520_at	*GYPA*	Glycophorin A	2993	1.06E-12	1.43E-11	-3.26
205495_s_at	*GNLY*	granulysin	10578	1.15E-10	1.02E-09	-3.26
228376_at	*GGTA1*	glycoprotein, alpha-galactosyltransferase 1	2681	2.48E-25	2.23E-23	-3.26
205944_s_at	*CLTCL1*	clathrin, heavy chain-like 1	8218	1.76E-36	5.57E-34	-3.26
210031_at	*CD247*	CD247 molecule	919	6.56E-15	1.30E-13	-3.26
210164_at	*GZMB*	granzyme B (granzyme 2, cytotoxic T-lymphocyte-associated serine esterase 1)	3002	4.52E-14	7.72E-13	-3.27
240061_at	*PION*	CDNA clone IMAGE:6060501	54103	1.57E-12	2.05E-11	-3.27
203922_s_at	*CYBB*	cytochrome b-245, beta polypeptide	1536	1.98E-33	4.07E-31	-3.27
244235_at	*IVNS1ABP*	influenza virus NS1A binding protein	10625	1.78E-24	1.46E-22	-3.27
221234_s_at	*BACH2*	BTB and CNC homology 1, basic leucine zipper transcription factor 2	60468	8.47E-23	5.63E-21	-3.28
232623_at	*LOC100128751*	INM04	100128751	8.19E-12	9.25E-11	-3.28
204865_at	*CA3*	carbonic anhydrase III, muscle specific	761	1.42E-06	5.64E-06	-3.28
237544_at				7.58E-20	3.27E-18	-3.29
201363_s_at	*IVNS1ABP*	influenza virus NS1A binding protein	10625	6.83E-40	2.88E-37	-3.29
234980_at	*TMEM56*	transmembrane protein 56	148534	7.40E-18	2.38E-16	-3.29
214769_at	*CLCN4*	chloride channel 4	1183	3.77E-17	1.09E-15	-3.30
229377_at	*GRTP1*	growth hormone regulated TBC protein 1	79774	1.09E-08	6.56E-08	-3.30
239152_at				4.74E-15	9.55E-14	-3.31
209791_at	*PADI2*	peptidyl arginine deiminase, type II	11240	1.76E-26	1.92E-24	-3.31
235380_at				4.32E-26	4.33E-24	-3.31
222895_s_at	*BCL11B*	B-cell CLL/lymphoma 11B (zinc finger protein)	64919	7.08E-12	8.09E-11	-3.32
204959_at	*MNDA*	myeloid cell nuclear differentiation antigen	4332	2.60E-38	9.41E-36	-3.32
221958_s_at	*GPR177*	G protein-coupled receptor 177	79971	7.48E-21	3.87E-19	-3.33
228377_at	*KLHL14*	kelch-like 14 (Drosophila)	57565	4.16E-29	5.77E-27	-3.35
222838_at	*SLAMF7*	SLAM family member 7	57823	2.20E-18	7.81E-17	-3.35
231969_at	*STOX2*	storkhead box 2	56977	2.06E-26	2.22E-24	-3.36
37145_at	*GNLY*	granulysin	10578	6.98E-15	1.37E-13	-3.37
207992_s_at	*AMPD3*	adenosine monophosphate deaminase (isoform E)	272	0.00E+00	0.00E+00	-3.37
206222_at	*TNFRSF10C*	tumor necrosis factor receptor superfamily, member 10c, decoy without an intracellular domain	8794	1.61E-18	5.82E-17	-3.37
235568_at	*C19orf59*	chromosome 19 open reading frame 59	199675	1.90E-36	5.94E-34	-3.38
212912_at	*RPS6KA2*	ribosomal protein S6 kinase, 90kDa, polypeptide 2	6196	2.35E-23	1.69E-21	-3.39
203021_at	*SLPI*	secretory leukocyte peptidase inhibitor	6590	3.34E-23	2.34E-21	-3.39
204446_s_at	*ALOX5*	arachidonate 5-lipoxygenase	240	1.42E-38	5.27E-36	-3.40
223412_at	*KBTBD7*	kelch repeat and BTB (POZ) domain containing 7	84078	3.41E-34	7.54E-32	-3.40
1559391_s_at				1.06E-17	3.36E-16	-3.40
236083_at	*BCL2L15*	BCL2-like 15	440603	3.61E-38	1.29E-35	-3.40

Probe Set	Gene Symbol	Gene Title	Entrez Gene	*p*-value	q-value	FC
212805_at	*PRUNE2*	prune homolog 2 (Drosophila)	158471	6.33E-17	1.76E-15	-3.41
210321_at	*GZMH*	granzyme H (cathepsin G-like 2, protein h-CCPX)	2999	7.34E-08	3.76E-07	-3.41
232122_s_at	*VEPH1*	ventricular zone expressed PH domain homolog 1 (zebrafish)	79674	5.99E-25	5.14E-23	-3.42
207717_s_at	*PKP2*	plakophilin 2	5318	4.40E-23	3.05E-21	-3.42
226806_s_at				1.24E-21	7.08E-20	-3.43
200998_s_at	*CKAP4*	cytoskeleton-associated protein 4	10970	4.66E-36	1.38E-33	-3.45
230833_at	*ACRBP*	acrosin binding protein	84519	8.44E-19	3.14E-17	-3.45
1569225_a_at	*SCML4*	sex comb on midleg-like 4 (Drosophila)	256380	3.47E-20	1.61E-18	-3.45
231166_at	*GPR155*	G protein-coupled receptor 155	151556	9.61E-25	8.06E-23	-3.46
1554952_s_at	*NLRP12*	NLR family, pyrin domain containing 12	91662	1.52E-15	3.39E-14	-3.47
1565544_at	*RNF141*	ring finger protein 141	50862	2.98E-24	2.39E-22	-3.47
230856_at				5.25E-43	2.87E-40	-3.47
205590_at	*RASGRP1*	RAS guanyl releasing protein 1 (calcium and DAG-regulated)	10125	1.78E-15	3.88E-14	-3.48
216560_x_at	*IGL@*	immunoglobulin lambda locus	3535	1.33E-11	1.43E-10	-3.49
227646_at	*EBF1*	early B-cell factor 1	1879	1.57E-20	7.70E-19	-3.50
227312_at	*SNTB2*	syntrophin, beta 2 (dystrophin-associated protein A1, 59kDa, basic component 2)	6645	1.22E-31	2.16E-29	-3.51
1562289_at				5.97E-22	3.56E-20	-3.51
204546_at	*KIAA0513*	KIAA0513	9764	4.52E-33	8.79E-31	-3.51
207206_s_at	*ALOX12*	arachidonate 12-lipoxygenase	239	4.33E-25	3.80E-23	-3.51
227498_at				2.81E-19	1.12E-17	-3.52
204006_s_at	*FCGR3A / FCGR3B*	Fc fragment of IgG, low affinity IIIa, receptor (CD16a) / Fc fragment of IgG, low affinity IIIb, receptor (CD16b)	2214 / 2215	3.96E-07	1.76E-06	-3.52
215332_s_at	*CD8B*	CD8b molecule	926	1.02E-16	2.78E-15	-3.52
1569666_s_at				8.80E-26	8.41E-24	-3.53
204891_s_at	*LCK*	lymphocyte-specific protein tyrosine kinase	3932	1.71E-11	1.79E-10	-3.53
201362_at	*IVNS1ABP*	influenza virus NS1A binding protein	10625	9.81E-45	6.14E-42	-3.54
211890_x_at	*CAPN3*	calpain 3, (p94)	825	2.53E-25	2.26E-23	-3.55
205442_at	*MFAP3L*	microfibrillar-associated protein 3-like	9848	1.67E-27	1.98E-25	-3.55
223767_at	*GPR84*	G protein-coupled receptor 84	53831	1.52E-26	1.66E-24	-3.55
210789_x_at	*CEACAM3*	carcinoembryonic antigen-related cell adhesion molecule 3	1084	6.50E-32	1.16E-29	-3.56
220418_at	*UBASH3A*	ubiquitin associated and SH3 domain containing, A	53347	4.38E-18	1.48E-16	-3.56
204007_at	*FCGR3B*	Fc fragment of IgG, low affinity IIIb, receptor (CD16b)	2215	5.28E-11	5.01E-10	-3.57
211596_s_at	*LRIG1*	leucine-rich repeats and immunoglobulin-like domains 1	26018	5.22E-32	9.42E-30	-3.57
1570585_at	*MPZL3*	myelin protein zero-like 3	196264	1.47E-24	1.21E-22	-3.57
204995_at	*CDK5R1*	cyclin-dependent kinase 5, regulatory subunit 1 (p35)	8851	1.08E-22	7.05E-21	-3.58
1558971_at	*C6orf190*	chromosome 6 open reading frame 190	387357	6.42E-13	9.05E-12	-3.60
207329_at	*MMP8*	matrix metallopeptidase 8 (neutrophil collagenase)	4317	4.22E-22	2.56E-20	-3.61
222078_at	*PKLR*	pyruvate kinase, liver and RBC	5313	1.28E-30	2.06E-28	-3.62
222071_s_at	*SLCO4C1*	solute carrier organic anion transporter family, member 4C1	353189	3.24E-30	5.13E-28	-3.62
231093_at	*FCRL3*	Fc receptor-like 3	115352	3.34E-20	1.56E-18	-3.62
219812_at	*PVRIG*	poliovirus receptor related immunoglobulin domain containing	79037	7.52E-21	3.88E-19	-3.62
206666_at	*GZMK*	granzyme K (granzyme 3; tryptase II)	3003	4.52E-09	2.95E-08	-3.62
220421_at	*BTNL8 / LOC653117*	butyrophilin-like 8 / similar to B7h.4	653117 / 79908	2.76E-18	9.65E-17	-3.62
209771_x_at	*CD24*	CD24 molecule	100133941	0.00E+00	0.00E+00	-3.62

Probe Set	Gene Symbol	Gene Title	Entrez Gene	*p*-value	q-value	FC
203765_at	*GCA*	grancalcin, EF-hand calcium binding protein	25801	0.00E+00	0.00E+00	-3.63
205831_at	*CD2*	CD2 molecule	914	5.73E-14	9.60E-13	-3.63
226733_at	*PFKFB2*	6-phosphofructo-2-kinase/fructose-2,6-biphosphatase 2	5208	1.33E-23	9.81E-22	-3.63
237590_at				8.35E-21	4.29E-19	-3.63
204881_s_at	*UGCG*	UDP-glucose ceramide glucosyltransferase	7357	7.08E-35	1.73E-32	-3.63
214470_at	*KLRB1*	killer cell lectin-like receptor subfamily B, member 1	3820	6.92E-18	2.23E-16	-3.63
229778_at	*C12orf39*	chromosome 12 open reading frame 39	80763	1.20E-22	7.73E-21	-3.63
209212_s_at	*KLF5*	Kruppel-like factor 5 (intestinal)	688	7.56E-27	8.51E-25	-3.63
237515_at	*TMEM56*	transmembrane protein 56	148534	2.32E-19	9.33E-18	-3.65
205484_at	*SIT1*	signaling threshold regulating transmembrane adaptor 1	27240	3.06E-16	7.72E-15	-3.65
1559050_at	*HCG27*	HLA complex group 27	253018	1.36E-22	8.73E-21	-3.65
224967_at	*UGCG*	UDP-glucose ceramide glucosyltransferase	7357	0.00E+00	0.00E+00	-3.66
236796_at				1.70E-16	4.50E-15	-3.66
214735_at	*PIP3-E*	phosphoinositide-binding protein PIP3-E	26034	2.24E-34	4.99E-32	-3.66
205927_s_at	*CTSE*	cathepsin E	1510	2.02E-12	2.58E-11	-3.66
206371_at	*FOLR3*	folate receptor 3 (gamma)	2352	2.86E-07	1.31E-06	-3.67
209992_at	*PFKFB2*	6-phosphofructo-2-kinase/fructose-2,6-biphosphatase 2	5208	7.60E-37	2.47E-34	-3.67
203986_at	*STBD1*	starch binding domain 1	8987	5.73E-33	1.10E-30	-3.69
218373_at	*AKTIP*	AKT interacting protein	64400	2.94E-41	1.37E-38	-3.69
206785_s_at	*KLRC1 / KLRC2*	killer cell lectin-like receptor subfamily C, member 1 / killer cell lectin-like receptor subfamily C, member 2	3821 / 3822	2.71E-13	4.06E-12	-3.71
205758_at	*CD8A*	CD8a molecule	925	1.60E-14	2.96E-13	-3.72
210606_x_at	*KLRD1*	killer cell lectin-like receptor subfamily D, member 1	3824	5.63E-17	1.58E-15	-3.73
200999_s_at	*CKAP4*	cytoskeleton-associated protein 4	10970	6.92E-43	3.70E-40	-3.73
236979_at	*BCL2L15*	BCL2-like 15	440603	1.54E-44	9.42E-42	-3.75
238029_s_at	*SLC16A14*	solute carrier family 16, member 14 (monocarboxylic acid transporter 14)	151473	9.43E-21	4.79E-19	-3.75
220646_s_at	*KLRF1*	killer cell lectin-like receptor subfamily F, member 1	51348	2.67E-14	4.73E-13	-3.75
224707_at	*C5orf32*	chromosome 5 open reading frame 32	84418	0.00E+00	0.00E+00	-3.76
226479_at	*KBTBD6*	kelch repeat and BTB (POZ) domain containing 6	89890	5.38E-36	1.57E-33	-3.76
219529_at	*CLIC3*	chloride intracellular channel 3	9022	3.36E-11	3.32E-10	-3.77
208650_s_at	*CD24*	CD24 molecule	100133941	7.20E-35	1.74E-32	-3.78
203757_s_at	*CEACAM6*	carcinoembryonic antigen-related cell adhesion molecule 6 (non-specific cross reacting antigen)	4680	1.24E-30	2.00E-28	-3.79
210772_at	*FPR2*	formyl peptide receptor 2	2358	1.49E-22	9.47E-21	-3.79
236981_at	*C17orf99*	chromosome 17 open reading frame 99	100141515	1.13E-15	2.58E-14	-3.80
236199_at				1.11E-28	1.47E-26	-3.80
232784_at				2.43E-23	1.73E-21	-3.80
216379_x_at	*CD24*	CD24 molecule	100133941	0.00E+00	0.00E+00	-3.80
221558_s_at	*LEF1*	lymphoid enhancer-binding factor 1	51176	2.10E-19	8.56E-18	-3.81
214475_x_at	*CAPN3*	calpain 3, (p94)	825	9.91E-28	1.22E-25	-3.82
1553111_a_at	*KBTBD6*	kelch repeat and BTB (POZ) domain containing 6	89890	3.43E-30	5.40E-28	-3.83
215184_at	*DAPK2*	death-associated protein kinase 2	23604	8.92E-38	3.09E-35	-3.83
235816_s_at	*RGL4*	ral guanine nucleotide dissociation stimulator-like 4	266747	1.64E-35	4.34E-33	-3.84
214798_at	*ATP2C2*	ATPase, Ca++ transporting, type 2C, member 2	9914	1.83E-42	9.42E-40	-3.84
220485_s_at	*SIRPG*	signal-regulatory protein gamma	55423	2.03E-24	1.65E-22	-3.84
206804_at	*CD3G*	CD3g molecule, gamma (CD3-TCR complex)	917	1.24E-16	3.33E-15	-3.86
221142_s_at	*PECR*	peroxisomal trans-2-enoyl-CoA reductase	55825	2.40E-38	8.81E-36	-3.86
244387_at				4.91E-18	1.62E-16	-3.86

Probe Set	Gene Symbol	Gene Title	Entrez Gene	*p*-value	q-value	FC
211657_at	*CEACAM6*	carcinoembryonic antigen-related cell adhesion molecule 6 (non-specific cross reacting antigen)	4680	6.50E-35	1.62E-32	-3.87
44790_s_at	*C13orf18 / LOC728970*	chromosome 13 open reading frame 18 / hypothetical LOC728970	728970 / 80183	1.96E-21	1.09E-19	-3.87
210610_at	*CEACAM1*	carcinoembryonic antigen-related cell adhesion molecule 1 (biliary glycoprotein)	634	1.71E-34	3.88E-32	-3.87
229230_at	*OSTalpha*	organic solute transporter alpha	200931	6.06E-14	1.01E-12	-3.87
207795_s_at	*KLRD1*	killer cell lectin-like receptor subfamily D, member 1	3824	1.53E-15	3.40E-14	-3.87
215894_at	*PTGDR*	prostaglandin D2 receptor (DP)	5729	1.05E-19	4.40E-18	-3.88
211902_x_at	*TRA@*	T cell receptor alpha locus	6955	3.26E-18	1.12E-16	-3.88
1569387_at				4.14E-19	1.60E-17	-3.88
214617_at	*PRF1*	perforin 1 (pore forming protein)	5551	5.92E-16	1.42E-14	-3.89
204411_at	*KIF21B*	kinesin family member 21B	23046	2.56E-41	1.21E-38	-3.90
211816_x_at	*FCAR*	Fc fragment of IgA, receptor for	2204	2.10E-26	2.24E-24	-3.90
206676_at	*CEACAM8*	carcinoembryonic antigen-related cell adhesion molecule 8	1088	1.40E-45	9.72E-43	-3.91
202761_s_at	*SYNE2*	spectrin repeat containing, nuclear envelope 2	23224	1.42E-29	2.07E-27	-3.91
229584_at	*LRRK2*	leucine-rich repeat kinase 2	120892	1.66E-17	5.06E-16	-3.91
210452_x_at	*CYP4F2*	cytochrome P450, family 4, subfamily F, polypeptide 2	8529	1.10E-26	1.22E-24	-3.92
217977_at	*SEPX1*	selenoprotein X, 1	51734	4.73E-34	1.02E-31	-3.93
228599_at	*MS4A1*	membrane-spanning 4-domains, subfamily A, member 1	931	3.59E-12	4.33E-11	-3.93
225987_at	*STEAP4*	STEAP family member 4	79689	2.56E-09	1.75E-08	-3.93
227030_at				2.05E-14	3.72E-13	-3.93
206522_at	*LOC642103 / MGAM*	similar to Maltase-glucoamylase, intestinal / maltase-glucoamylase (alpha-glucosidase)	642103 / 8972	1.41E-21	7.93E-20	-3.94
207840_at	*CD160*	CD160 molecule	11126	1.44E-23	1.06E-21	-3.94
242197_x_at	*CD36*	CD36 antigen	948	1.09E-10	9.66E-10	-3.94
266_s_at	*CD24*	CD24 molecule	100133941	0.00E+00	0.00E+00	-3.95
209447_at	*SYNE1*	spectrin repeat containing, nuclear envelope 1	23345	2.18E-41	1.06E-38	-3.98
210773_s_at	*FPR2*	formyl peptide receptor 2	2358	1.12E-19	4.67E-18	-3.99
220000_at	*SIGLEC5*	sialic acid binding Ig-like lectin 5	8778	4.47E-29	6.17E-27	-3.99
205513_at	*TCN1*	transcobalamin I (vitamin B12 binding protein, R binder family)	6947	2.93E-32	5.37E-30	-4.00
205119_s_at	*FPR1*	formyl peptide receptor 1	2357	6.51E-31	1.08E-28	-4.00
205174_s_at	*QPCT*	glutaminyl-peptide cyclotransferase	25797	2.77E-31	4.78E-29	-4.01
205863_at	*S100A12*	S100 calcium binding protein A12	6283	0.00E+00	0.00E+00	-4.02
205267_at	*POU2AF1*	POU class 2 associating factor 1	5450	2.73E-19	1.09E-17	-4.03
223422_s_at	*ARHGAP24*	Rho GTPase activating protein 24	83478	2.11E-33	4.29E-31	-4.03
211806_s_at	*KCNJ15*	potassium inwardly-rectifying channel, subfamily J, member 15	3772	6.29E-12	7.27E-11	-4.04
221765_at	*UGCG*	UDP-glucose ceramide glucosyltransferase	7357	1.35E-37	4.60E-35	-4.06
206576_s_at	*CEACAM1*	carcinoembryonic antigen-related cell adhesion molecule 1 (biliary glycoprotein)	634	3.11E-29	4.33E-27	-4.08
235146_at	*TMCC3*	transmembrane and coiled-coil domain family 3	57458	5.50E-17	1.54E-15	-4.08
208651_x_at	*CD24*	CD24 molecule	100133941	7.76E-43	4.07E-40	-4.13
222934_s_at	*CLEC4E*	C-type lectin domain family 4, member E	26253	6.93E-18	2.23E-16	-4.14
211307_s_at	*FCAR*	Fc fragment of IgA, receptor for	2204	1.04E-19	4.38E-18	-4.14
209211_at	*KLF5*	Kruppel-like factor 5 (intestinal)	688	2.31E-32	4.33E-30	-4.14
205291_at	*IL2RB*	interleukin 2 receptor, beta	3560	2.77E-19	1.10E-17	-4.15
242013_at	*BCL2L15*	BCL2-like 15	440603	5.61E-45	3.79E-42	-4.15
214907_at	*CEACAM21*	carcinoembryonic antigen-related cell adhesion molecule 21	90273	5.52E-36	1.57E-33	-4.16
230690_at	*TUBB1*	tubulin, beta 1	81027	2.46E-29	3.51E-27	-4.16

Probe Set	Gene Symbol	Gene Title	Entrez Gene	*p*-value	q-value	FC
205627_at	*CDA*	cytidine deaminase	978	7.60E-41	3.42E-38	-4.17
205821_at	*KLRK1*	killer cell lectin-like receptor subfamily K, member 1	22914	1.99E-17	5.96E-16	-4.18
226489_at	*TMCC3*	transmembrane and coiled-coil domain family 3	57458	9.28E-15	1.78E-13	-4.21
221969_at				8.98E-23	5.93E-21	-4.22
209772_s_at	*CD24*	CD24 molecule	100133941	2.67E-31	4.63E-29	-4.23
216605_s_at	*CEACAM21*	carcinoembryonic antigen-related cell adhesion molecule 21	90273	8.35E-31	1.37E-28	-4.25
215102_at	*DPY19L1P1*	dpy-19-like 1 pseudogene 1 (C. elegans)	100129460	1.53E-33	3.19E-31	-4.26
1553177_at	*SH2D1B*	SH2 domain containing 1B	117157	4.56E-13	6.60E-12	-4.27
218454_at	*FLJ22662*	hypothetical protein FLJ22662	79887	0.00E+00	0.00E+00	-4.27
211883_x_at	*CEACAM1*	carcinoembryonic antigen-related cell adhesion molecule 1 (biliary glycoprotein)	634	1.89E-34	4.27E-32	-4.27
231644_at				6.64E-25	5.64E-23	-4.28
203434_s_at	*MME*	membrane metallo-endopeptidase	4311	2.01E-11	2.08E-10	-4.28
210972_x_at	*TRA@ / TRAC / TRAJ17 / TRAV20*	T cell receptor alpha locus / T cell receptor alpha constant / T cell receptor alpha joining 17 / T cell receptor alpha variable 20	28663 / 28738 / 28755 / 6955	3.13E-18	1.08E-16	-4.29
223894_s_at	*AKTIP*	AKT interacting protein	64400	1.40E-45	9.72E-43	-4.30
221556_at	*CDC14B*	CDC14 cell division cycle 14 homolog B (S. cerevisiae)	8555	0.00E+00	0.00E+00	-4.31
207674_at	*FCAR*	Fc fragment of IgA, receptor for	2204	2.18E-25	1.97E-23	-4.31
235764_at				5.08E-32	9.26E-30	-4.31
209671_x_at	*TRA@ / TRAC*	T cell receptor alpha locus / T cell receptor alpha constant	28755 / 6955	1.20E-17	3.73E-16	-4.32
207979_s_at	*CD8B*	CD8b molecule	926	2.13E-12	2.70E-11	-4.32
233302_at				2.77E-12	3.42E-11	-4.34
220570_at	*RETN*	resistin	56729	7.34E-36	2.02E-33	-4.37
214523_at	*CEBPE*	CCAAT/enhancer binding protein (C/EBP), epsilon	1053	8.18E-39	3.13E-36	-4.37
205488_at	*GZMA*	granzyme A (granzyme 1, cytotoxic T-lymphocyte-associated serine esterase 3)	3001	1.01E-14	1.93E-13	-4.37
206177_s_at	*ARG1*	arginase, liver	383	6.87E-44	4.01E-41	-4.39
207890_s_at	*MMP25*	matrix metallopeptidase 25	64386	6.93E-21	3.61E-19	-4.43
205798_at	*IL7R*	interleukin 7 receptor	3575	1.54E-17	4.71E-16	-4.46
1552553_a_at	*NLRC4*	NLR family, CARD domain containing 4	58484	8.42E-26	8.10E-24	-4.47
206324_s_at	*DAPK2*	death-associated protein kinase 2	23604	3.61E-36	1.12E-33	-4.48
236782_at	*SAMD3*	sterile alpha motif domain containing 3	154075	7.33E-14	1.21E-12	-4.51
233289_at				5.87E-30	8.92E-28	-4.52
242827_x_at				2.42E-23	1.73E-21	-4.53
1552772_at	*CLEC4D*	C-type lectin domain family 4, member D	338339	5.96E-30	8.99E-28	-4.53
220474_at	*SLC25A21*	solute carrier family 25 (mitochondrial oxodicarboxylate carrier), member 21	89874	4.74E-18	1.58E-16	-4.54
210944_s_at	*CAPN3*	calpain 3, (p94)	825	0.00E+00	0.00E+00	-4.55
211889_x_at	*CEACAM1*	carcinoembryonic antigen-related cell adhesion molecule 1 (biliary glycoprotein)	634	2.79E-35	7.24E-33	-4.56
1566825_at				7.01E-26	6.84E-24	-4.58
209369_at	*ANXA3*	annexin A3	306	0.00E+00	0.00E+00	-4.58
242397_at				7.98E-14	1.30E-12	-4.59
226188_at	*HSPC159*	galectin-related protein	29094	0.00E+00	0.00E+00	-4.60
1552348_at	*PRSS33*	protease, serine, 33	260429	1.36E-26	1.50E-24	-4.61
242824_at				3.53E-20	1.63E-18	-4.64
209670_at	*TRAC*	T cell receptor alpha constant	28755	4.25E-19	1.64E-17	-4.65
227182_at	*SUSD3*	sushi domain containing 3	203328	2.98E-39	1.19E-36	-4.67
1558972_s_at	*C6orf190*	chromosome 6 open reading frame 190	387357	5.08E-13	7.30E-12	-4.70

Probe Set	Gene Symbol	Gene Title	Entrez Gene	*p*-value	q-value	FC
206043_s_at	*ATP2C2*	ATPase, Ca++ transporting, type 2C, member 2	9914	3.80E-42	1.91E-39	-4.71
232277_at				3.23E-33	6.43E-31	-4.76
229759_s_at	*VEPH1*	ventricular zone expressed PH domain homolog 1 (zebrafish)	79674	8.58E-24	6.52E-22	-4.76
213906_at	*MYBL1*	v-myb myeloblastosis viral oncogene homolog (avian)-like 1	4603	1.16E-12	1.56E-11	-4.80
210356_x_at	*MS4A1*	membrane-spanning 4-domains, subfamily A, member 1	931	4.91E-14	8.32E-13	-4.81
211413_s_at	*PADI4*	peptidyl arginine deiminase, type IV	23569	2.00E-27	2.34E-25	-4.83
230383_x_at				3.16E-13	4.69E-12	-4.86
214295_at	*KIAA0485*	hypothetical LOC57235	57235	6.15E-23	4.18E-21	-4.88
218330_s_at	*NAV2*	neuron navigator 2	89797	3.06E-29	4.29E-27	-4.93
217418_x_at	*MS4A1*	membrane-spanning 4-domains, subfamily A, member 1	931	3.96E-13	5.77E-12	-4.94
210004_at	*OLR1*	oxidized low density lipoprotein (lectin-like) receptor 1	4973	8.05E-41	3.56E-38	-4.99
202286_s_at	*TACSTD2*	tumor-associated calcium signal transducer 2	4070	2.67E-35	6.99E-33	-5.01
237154_at				7.87E-27	8.82E-25	-5.03
226218_at	*IL7R*	interleukin 7 receptor	3575	4.92E-21	2.62E-19	-5.07
226685_at	*SNTB2*	syntrophin, beta 2 (dystrophin-associated protein A1, 59kDa, basic component 2)	6645	1.69E-34	3.88E-32	-5.08
1553295_at	*ABCA13*	ATP-binding cassette, sub-family A (ABC1), member 13	154664	1.40E-45	9.72E-43	-5.08
207815_at	*PF4V1*	platelet factor 4 variant 1	5197	4.57E-16	1.12E-14	-5.09
1552773_at	*CLEC4D*	C-type lectin domain family 4, member D	338339	9.07E-34	1.94E-31	-5.09
211163_s_at	*TNFRSF10C*	tumor necrosis factor receptor superfamily, member 10c, decoy without an intracellular domain	8794	2.67E-18	9.36E-17	-5.10
238439_at	*ANKRD22*	ankyrin repeat domain 22	118932	6.84E-40	2.88E-37	-5.20
223552_at	*LRRC4*	leucine rich repeat containing 4	64101	9.13E-30	1.34E-27	-5.21
206208_at	*CA4*	carbonic anhydrase IV	762	6.24E-25	5.32E-23	-5.21
228592_at	*MS4A1*	membrane-spanning 4-domains, subfamily A, member 1	931	2.10E-18	7.45E-17	-5.22
219243_at	*GIMAP4*	GTPase, IMAP family member 4	55303	2.80E-12	3.45E-11	-5.24
235982_at	*FCRL1*	Fc receptor-like 1	115350	4.39E-34	9.55E-32	-5.26
229770_at	*GLT1D1*	glycosyltransferase 1 domain containing 1	144423	7.01E-45	4.50E-42	-5.28
220811_at	*PRG3*	proteoglycan 3	10394	2.28E-14	4.10E-13	-5.35
220001_at	*PADI4*	peptidyl arginine deiminase, type IV	23569	0.00E+00	0.00E+00	-5.42
206464_at	*BMX*	BMX non-receptor tyrosine kinase	660	2.01E-25	1.83E-23	-5.43
209396_s_at	*CHI3L1*	chitinase 3-like 1 (cartilage glycoprotein-39)	1116	3.57E-37	1.19E-34	-5.44
203435_s_at	*MME*	membrane metallo-endopeptidase	4311	1.19E-09	8.71E-09	-5.48
203828_s_at	*IL32*	interleukin 32	9235	1.56E-08	9.14E-08	-5.53
206514_s_at	*CYP4F2 / CYP4F3*	cytochrome P450, family 4, subfamily F, polypeptide 2 / cytochrome P450, family 4, subfamily F, polypeptide 3	4051 / 8529	4.05E-34	8.88E-32	-5.57
223304_at	*SLC37A3*	solute carrier family 37 (glycerol-3-phosphate transporter), member 3	84255	1.38E-24	1.14E-22	-5.75
211412_at	*PADI4*	peptidyl arginine deiminase, type IV	23569	4.51E-29	6.19E-27	-5.79
207094_at	*IL8RA*	interleukin 8 receptor, alpha	3577	8.07E-17	2.23E-15	-5.83
206697_s_at	*HP / HPR*	haptoglobin / haptoglobin-related protein	3240 / 3250	0.00E+00	0.00E+00	-5.85
243968_x_at	*FCRL1*	Fc receptor-like 1	115350	3.44E-37	1.16E-34	-6.01
221211_s_at	*C21orf7*	chromosome 21 open reading frame 7	56911	5.12E-18	1.69E-16	-6.01
204636_at	*COL17A1*	collagen, type XVII, alpha 1	1308	5.34E-38	1.88E-35	-6.03
220528_at	*VNN3*	vanin 3	55350	1.20E-20	5.97E-19	-6.21
236422_at				8.71E-30	1.30E-27	-6.26

Probe Set	Gene Symbol	Gene Title	Entrez Gene	*p*-value	q-value	FC
1553604_at	*ABCA13*	ATP-binding cassette, sub-family A (ABC1), member 13	154664	6.20E-42	3.06E-39	-6.26
238066_at	*RBP7*	retinol binding protein 7, cellular	116362	4.98E-41	2.28E-38	-6.28
206209_s_at	*CA4*	carbonic anhydrase IV	762	1.86E-24	1.52E-22	-6.29
207072_at	*IL18RAP*	interleukin 18 receptor accessory protein	8807	7.15E-36	2.00E-33	-6.30
209395_at	*CHI3L1*	chitinase 3-like 1 (cartilage glycoprotein-39)	1116	1.40E-45	9.72E-43	-6.33
210119_at	*KCNJ15*	potassium inwardly-rectifying channel, subfamily J, member 15	3772	2.58E-16	6.61E-15	-6.41
220068_at	*VPREB3*	pre-B lymphocyte 3	29802	2.35E-29	3.36E-27	-6.45
207384_at	*PGLYRP1*	peptidoglycan recognition protein 1	8993	0.00E+00	0.00E+00	-6.48
209498_at	*CEACAM1*	carcinoembryonic antigen-related cell adhesion molecule 1 (biliary glycoprotein)	634	4.48E-44	2.68E-41	-6.54
208470_s_at	*HP / HPR*	haptoglobin / haptoglobin-related protein	3240 / 3250	0.00E+00	0.00E+00	-6.61
232027_at	*SYNE1*	Spectrin repeat containing, nuclear envelope 1, mRNA (cDNA clone IMAGE:4830497)	23345	7.01E-45	4.50E-42	-6.70
207008_at	*IL8RB*	interleukin 8 receptor, beta	3579	3.83E-10	3.03E-09	-6.73
1553605_a_at	*ABCA13*	ATP-binding cassette, sub-family A (ABC1), member 13	154664	1.99E-43	1.13E-40	-7.09
231771_at	*GJB6*	gap junction protein, beta 6, 30kDa	10804	2.12E-22	1.33E-20	-7.32
203936_s_at	*MMP9*	matrix metallopeptidase 9 (gelatinase B, 92kDa gelatinase, 92kDa type IV collagenase)	4318	0.00E+00	0.00E+00	-7.58
234632_x_at				1.69E-23	1.23E-21	-8.02
229738_at	*DNAH10*	dynein, axonemal, heavy chain 10	196385	0.00E+00	0.00E+00	-8.33
1553518_at	*DEFT1P*	defensin, theta 1 pseudogene	170949	0.00E+00	0.00E+00	-8.96
204623_at	*TFF3*	trefoil factor 3 (intestinal)	7033	0.00E+00	0.00E+00	-9.30
210262_at	*CRISP2*	cysteine-rich secretory protein 2	7180	9.93E-41	4.32E-38	-9.75
212531_at	*LCN2*	lipocalin 2	3934	0.00E+00	0.00E+00	-10.01
208168_s_at	*CHIT1*	chitinase 1 (chitotriosidase)	1118	1.52E-33	3.19E-31	-10.22
39318_at	*TCL1A*	T-cell leukemia/lymphoma 1A	8115	9.24E-23	6.07E-21	-10.30
219669_at	*CD177*	CD177 molecule	57126	2.90E-32	5.35E-30	-10.72
209995_s_at	*TCL1A*	T-cell leukemia/lymphoma 1A	8115	9.17E-26	8.69E-24	-11.53
207802_at	*CRISP3*	cysteine-rich secretory protein 3	10321	0.00E+00	0.00E+00	-11.67
205041_s_at	*ORM1 / ORM2*	orosomucoid 1 / orosomucoid 2	5004 / 5005	0.00E+00	0.00E+00	-11.69
205040_at	*ORM1*	orosomucoid 1	5004	0.00E+00	0.00E+00	-11.85
210244_at	*CAMP*	cathelicidin antimicrobial peptide	820	0.00E+00	0.00E+00	-12.13
206515_at	*CYP4F3*	cytochrome P450, family 4, subfamily F, polypeptide 3	4051	0.00E+00	0.00E+00	-12.58
214465_at	*ORM1 / ORM2*	orosomucoid 1 / orosomucoid 2	5004 / 5005	0.00E+00	0.00E+00	-13.03
212768_s_at	*OLFM4*	olfactomedin 4	10562	0.00E+00	0.00E+00	-14.31

Table S2. 24 genes that overlap between the AML-associated gene expression signature and a gene signature used to predict AML survival, as identified in Metzeler *et al.* [1].

Gene Symbol	Gene Title
BCAT1	branched chain amino-acid transaminase 1, cytosolic
CD109	CD109 molecule
COL24A1	collagen, type XXIV, alpha 1
FAM30A	family with sequence similarity 30, member A
FAM92A1	family with sequence similarity 92, member A1
FHL1	four and a half LIM domains 1
GPSM1	G-protein signaling modulator 1
GUCY1A3	guanylate cyclase 1, soluble, alpha 3
HIST1H2AD	histone cluster 1, H2ad
HOPX	HOP homeobox
IL23A	interleukin 23, alpha subunit p19
KIAA0125	*KIAA0125*
LAPTM4B	lysosomal protein transmembrane 4 beta
MAST4	microtubule associated serine/threonine kinase family member 4
MSI2	musashi homolog 2 (Drosophila)
NPDC1	neural proliferation, differentiation and control, 1
NPL	N-acetylneuraminate pyruvate lyase (dihydrodipicolinate synthase)
RAB34	RAB34, member RAS oncogene family
RUNX1	runt-related transcription factor 1
SCHIP1	schwannomin interacting protein 1
SHANK3	SH3 and multiple ankyrin repeat domains 3
TM4SF1	transmembrane 4 L six family member 1
TSC22D1	TSC22 domain family, member 1
ZBTB8	zinc finger and BTB domain containing 8

Table S3. 1589 genes targeted by AML-associated miRNAs. All genes are listed alongside the miRNA(s) predicted to target each gene, and the probability of preferentially conserved targeting (PCT) for each predicted miRNA-mRNA interaction.

Gene Symbol	Reference Sequence	miRNA(s) Predicted to Target the Gene	miR-125b P_{CT}	miR-126 P_{CT}	miR-142-3p P_{CT}	miR-155 P_{CT}	miR-223 P_{CT}	miR-29a P_{CT}	miR-29b P_{CT}
ABCB6	NM_005689	miR-29a						0.77	
ABCC4	NM_005845	miR-125b	0.69						
ABCE1	NM_001040876	miR-29a						0.78	
ABHD4	NM_022060	miR-29a						0.83	
ABHD5	NM_016006	miR-29a						0.99	
ABL2	NM_001136000	miR-142-3p, miR-155			0.82	0.66			
ACACA	NM_198834	miR-29a						0.83	
ACBD5	NM_001042473	miR-142-3p			0.7				
ACCN2	NM_001095	miR-29a						0.78	
ACER2	NM_001010887	miR-125b	0.97						
ACPL2	NM_001037172	miR-29b							0.87
ACSL4	NM_004458	miR-142-3p			0.72				
ACTA1	NM_001100	miR-155				0.55			
ACVR2A	NM_001616	miR-142-3p			0.56				
ADAM12	NM_003474	miR-29b							0.83
ADAM19	NM_033274	miR-29a						0.94	
ADAM9	NM_003816	miR-126		0.55					
ADAMTS10	NM_030957	miR-29a						0.91	
ADAMTS17	NM_139057	miR-29b							
ADAMTS18	NM_199355	miR-29b							0.91
ADAMTS19	NM_133638	miR-29a						0.85	
ADAMTS2	NM_014244	miR-29a						0.98	
ADAMTS3	NM_014243	miR-142-3p			0.8				
ADAMTS5	NM_007038	miR-29a						0.87	
ADAMTS6	NM_197941	miR-29b							0.67
ADAMTS7	NM_014272	miR-29b							0.79
ADAMTS9	NM_182920	miR-29b							
ADCY7	NM_001114	miR-223					0.61		
ADCY9	NM_001116	miR-142-3p			0.82				
ADCYAP1R1	NM_001118	miR-29a						0.83	
AFF1	NM_001166693	miR-142-3p			0.94				
AFF2	NM_001169122	miR-142-3p			0.7				
AFF4	NM_014423	miR-29a						0.78	
AGPAT4	NM_020133	miR-29a						0.83	
AGTRAP	NM_001040194	miR-155				0.55			
AHR	NM_001621	miR-29a						0.59	
AICDA	NM_020661	miR-155				0.55			
AIM1	NM_001624	miR-29b							0.68
AK1	NM_000476	miR-29b							0.78
AKAP13	NM_006738	miR-125b, miR-126, miR-29a	0.87	0.55				0.84	
AKAP2	NM_001004065	miR-29a						0.52	
AKAP5	NM_004857	miR-29a						0.94	
AKIRIN2	NM_018064	miR-142-3p			0.59				
AKT1S1	NM_001098632	miR-142-3p			0.82				
AKT3	NM_005465	miR-29a						0.77	

Gene Symbol	Reference Sequence	miRNA(s) Predicted to Target the Gene	miR-125b P_{CT}	miR-126 P_{CT}	miR-142-3p P_{CT}	miR-155 P_{CT}	miR-223 P_{CT}	miR-29a P_{CT}	miR-29b P_{CT}
AMFR	NM_001144	miR-29a						0.69	
AMMECR1	NM_001025580	miR-29a						0.89	
AMMECR1L	NM_001199140	miR-29b							0.82
AMOT	NM_001113490	miR-29a						0.79	
ANK1	NM_000037	miR-29a						0.83	
ANK3	NM_001149	miR-142-3p, miR-29a			0.83			0.83	
ANKFY1	NM_016376	miR-142-3p, miR-155			0.69	0.58			
ANKRD11	NM_013275	miR-142-3p			0.87				
ANKRD13B	NM_152345	miR-29a						0.92	
ANKRD13C	NM_030816	miR-29a						0.93	
ANKRD17	NM_032217	miR-223					0.52		
ANKRD40	NM_052855	miR-142-3p			0.7				
ANKRD49	NM_017704	miR-29a						0.83	
ANKRD52	NM_173595	miR-29a						> 0.99	
ANKRD57	NM_023016	miR-29a						0.67	
ANKS1A	NM_015245	miR-142-3p			0.78				
ANTXR2	NM_058172	miR-29a						0.97	
AP4E1	NM_007347	miR-29a						0.81	
APBA1	NM_001163	miR-125b	0.78						
APBB2	NM_001166050	miR-155				0.56			
APC	NM_000038	miR-142-3p, miR-155			0.83	0.54			
AQP4	NM_001650	miR-29b							0.81
ARF5	NM_001662	miR-29a						0.74	
ARFGEF2	NM_006420	miR-29a						0.75	
ARHGAP20	NM_020809	miR-29a						0.78	
ARHGAP31	NM_020754	miR-29a						0.81	
ARHGAP36	NM_144967	miR-29a						0.81	
ARHGEF10	NM_014629	miR-29a						0.84	
ARHGEF12	NM_001198665	miR-142-3p			0.77				
ARHGEF2	NM_001162383	miR-142-3p			0.82				
ARID2	NM_152641	miR-155				0.67			
ARID3A	NM_005224	miR-125b	0.67						
ARID5B	NM_032199	miR-142-3p			0.76				
ARL1	NM_001177	miR-142-3p			0.83				
ARL15	NM_019087	miR-142-3p			0.82				
ARL4A	NM_001037164	miR-29a						0.8	
ARMC8	NM_015396	miR-29b							0.65
ARNT	NM_001197325	miR-29a						0.83	
ARNTL	NM_001030272	miR-142-3p			0.79				
ARPP19	NM_006628	miR-29a						0.82	
ARRB2	NM_004313	miR-155				0.55			
ARRDC3	NM_020801	miR-142-3p, miR-29a			0.66			0.85	
ARRDC4	NM_183376	miR-29a						0.82	
ARSB	NM_000046	miR-29a						0.92	
ARVCF	NM_001670	miR-29a						0.82	
ASAP2	NM_001135191	miR-29a						0.87	
ASB7	NM_198243	miR-142-3p			0.8				
ASH1L	NM_018489	miR-142-3p							
ASPH	NM_001164756	miR-29a						0.62	

Gene Symbol	Reference Sequence	miRNA(s) Predicted to Target the Gene	miR-125b P_{CT}	miR-126 P_{CT}	miR-142-3p P_{CT}	miR-155 P_{CT}	miR-223 P_{CT}	miR-29a P_{CT}	miR-29b P_{CT}
ASXL1	NM_015338	miR-155				0.53			
ATAD2B	NM_001242338	miR-29a						0.99	
ATF7IP	NM_018179	miR-142-3p			0.83				
ATG14	NM_014924	miR-29a						0.83	
ATG16L1	NM_001190266	miR-142-3p			0.83				
ATG9A	NM_001077198	miR-29a						0.92	
ATP10D	NM_020453	miR-125b	0.87						
ATP1B1	NM_001677	miR-29a						0.81	
ATP1B4	NM_001142447	miR-29a						0.93	
ATP2B3	NM_001001344	miR-125b	0.68						
ATP2B4	NM_001001396	miR-29a						0.82	
ATP6V1A	NM_001690	miR-29a						0.52	
ATP6V1C1	NM_001695	miR-155				0.53			
ATP6V1G1	NM_004888	miR-155				0.55			
ATP7A	NM_000052	miR-223, miR-29a					0.57	0.86	
ATRN	NM_139321	miR-29a						0.69	
ATXN1	NM_000332	miR-125b, miR-29a	0.96					0.81	
ATXN1L	NM_001137675	miR-142-3p			0.86				
ATXN3	NM_001127696	miR-125b	0.7						
ATXN7	NM_000333	miR-142-3p			0.64				
ATXN7L1	NM_020725	miR-223					0.69		
B3GNT5	NM_032047	miR-29a						0.81	
BACE1	NM_001207048	miR-29a						0.97	
BACH1	NM_001186	miR-142-3p, miR-155			0.83	0.64			
BACH2	NM_001170794	miR-142-3p, miR-29a			0.87			0.93	
BAHD1	NM_014952	miR-29b							0.52
BAIAP2	NM_017450	miR-29a						0.81	
BAK1	NM_001188	miR-125b, miR-29a	0.95					0.92	
BAZ1A	NM_013448	miR-142-3p			0.8				
BBC3	NM_001127240	miR-125b, miR-29a	0.68					0.82	
BCL11A	NM_022893	miR-29b							0.81
BCL11B	NM_022898	miR-29b							0.79
BCL2	NM_000633	miR-125b	0.9						
BCL2L2	NM_001199839	miR-29a						0.92	
BCL9L	NM_182557	miR-29b							0.83
BCLAF1	NM_001077440	miR-142-3p			0.82				
BCORL1	NM_021946	miR-29b							0.97
BDH1	NM_004051	miR-29a						0.54	
BDNF	NM_001143805	miR-155				0.55			
BEAN1	NM_001136106	miR-29a						0.65	
BLMH	NM_000386	miR-29b							0.82
BMF	NM_001003940	miR-29b							0.93
BMP1	NM_001199	miR-29a						0.72	
BNC2	NM_017637	miR-142-3p			0.93				
BOD1	NM_001159651	miR-142-3p			0.96				
BPIL1	NM_025227	miR-155				0.53			
BRD3	NM_007371	miR-29b							0.97
BRPF3	NM_015695	miR-223					0.6		
BRWD1	NM_033656	miR-125b, miR-29a	0.91					0.83	

Gene Symbol	Reference Sequence	miRNA(s) Predicted to Target the Gene	miR-125b P_{CT}	miR-126 P_{CT}	miR-142-3p P_{CT}	miR-155 P_{CT}	miR-223 P_{CT}	miR-29a P_{CT}	miR-29b P_{CT}
BRWD3	NM_153252	miR-142-3p, miR-155, miR-29a			0.87	0.71		0.87	
BSDC1	NM_001143888	miR-29a						0.83	
BTBD7	NM_001002860	miR-142-3p, miR-29a			0.82			0.85	
BTG2	NM_006763	miR-29a						0.95	
BVES	NM_001199563	miR-29a						0.81	
C10orf12	NM_015652	miR-155				0.51			
C10orf18	NM_017782	miR-142-3p			0.91				
C10orf26	NM_001083913	miR-29a						0.93	
C10orf46	NM_153810	miR-155				0.68			
C11orf30	NM_020193	miR-29a						0.83	
C11orf41	NM_012194	miR-29b							0.89
C11orf57	NM_001082969	miR-29a						0.9	
C11orf87	NM_207645	miR-29a						0.89	
C13orf23	NM_025138	miR-29b							0.82
C14orf43	NM_001043318	miR-29a						0.81	
C15orf27	NM_152335	miR-29a						0.57	
C16orf70	NM_025187	miR-142-3p			0.77				
C16orf72	NM_014117	miR-29b							0.99
C16orf88	NM_001012991	miR-29a						0.94	
C17orf103	NM_152914	miR-125b	0.52						
C18orf1	NM_001003674	miR-29a						0.83	
C18orf25	NM_001008239	miR-142-3p			0.83				
C18orf42	NM_001145194	miR-29a						0.68	
C18orf62	NM_001037331	miR-29b							0.85
C19orf22	NM_138774	miR-29a						0.83	
C19orf39	NM_175871	miR-125b	0.59						
C19orf54	NM_198476	miR-125b	0.74						
C19orf6	NM_001033026	miR-29a						0.7	
C1orf130	NM_001010980	miR-29a						0.88	
C1orf144	NM_001114600	miR-125b	0.89						
C1orf183	NM_019099	miR-155				0.53			
C1orf21	NM_030806	miR-29b							0.81
C1orf212	NM_001164824	miR-29a						0.83	
C1orf9	NM_014283	miR-142-3p			0.63				
C1orf96	NM_145257	miR-29a						0.72	
C1QL2	NM_182528	miR-155				0.53			
C1QTNF6	NM_031910	miR-29b							0.96
C20orf11	NM_017896	miR-29a						0.83	
C20orf112	NM_080616	miR-142-3p, miR-29a			0.63			0.83	
C20orf194	NM_001009984	miR-142-3p			0.97				
C21orf91	NM_001100420	miR-29a						0.79	
C2orf88	NM_001042519	miR-29a						0.94	
C3orf18	NM_001171740	miR-155				0.55			
C4orf32	NM_152400	miR-142-3p, miR-29a			0.56			0.87	
C5orf13	NM_001142474	miR-29b							0.89
C5orf15	NM_020199	miR-29a						0.58	
C5orf24	NM_001135586	miR-29a						0.54	
C6orf168	NM_032511	miR-29b							0.98
C6orf204	NM_001042475	miR-29a						0.93	

Gene Symbol	Reference Sequence	miRNA(s) Predicted to Target the Gene	miR-125b P_{CT}	miR-126 P_{CT}	miR-142-3p P_{CT}	miR-155 P_{CT}	miR-223 P_{CT}	miR-29a P_{CT}	miR-29b P_{CT}
C6orf47	NM_021184	miR-125b, miR-142-3p	0.96		0.58				
C7orf60	NM_152556	miR-29a						0.85	
C9orf5	NM_032012	miR-155				0.55			
C9orf69	NM_152833	miR-155, miR-29a				0.55		0.83	
C9orf7	NM_001135775	miR-29b							0.79
C9orf72	NM_018325	miR-142-3p			0.77				
C9orf86	NM_001173988	miR-125b	0.94						
CA2	NM_000067	miR-29b							0.51
CA5B	NM_007220	miR-142-3p			0.67				
CACNA1C	NM_000719	miR-155				0.55			
CACNB2	NM_000724	miR-125b	0.55						
CACNG4	NM_014405	miR-29b							0.7
CALCR	NM_001164737	miR-29a						0.92	
CALM3	NM_005184	miR-29a						0.92	
CALU	NM_001130674	miR-29a						0.76	
CAMK1D	NM_153498	miR-29b							0.83
CAMK2G	NM_001204492	miR-29b							0.89
CAMKK2	NM_006549	miR-29a						0.93	
CAMSAP1	NM_015447	miR-126		0.56					
CAMSAP1L1	NM_203459	miR-29b							0.5
CAMTA1	NM_015215	miR-29a						0.78	
CANX	NM_001024649	miR-29a						0.84	
CAPN7	NM_014296	miR-29a						0.83	
CAPRIN1	NM_203364	miR-29a						0.58	
CARD11	NM_032415	miR-155				0.55			
CARHSP1	NM_001042476	miR-155				0.55			
CASK	NM_001126054	miR-142-3p			0.87				
CASP2	NM_001224	miR-125b	0.96						
CASP7	NM_001227	miR-29a						0.8	
CASZ1	NM_001079843	miR-142-3p			0.64				
CAV2	NM_001206747	miR-29b							0.83
CBL	NM_005188	miR-155				0.68			
CBLL1	NM_024814	miR-125b	0.66						
CBX1	NM_001127228	miR-29a						0.75	
CBX2	NM_005189	miR-29a						0.81	
CBX5	NM_001127321	miR-223, miR-29b					0.56		0.99
CBX6	NM_014292	miR-29b							
CCDC117	NM_173510	miR-29a						0.92	
CCDC28B	NM_024296	miR-29b							0.92
CCDC50	NM_174908	miR-29a						0.67	
CCDC6	NM_005436	miR-142-3p			0.95				
CCDC85C	NM_001144995	miR-223					0.5		
CCDC88A	NM_001135597	miR-142-3p, miR-29a			0.72			0.86	
CCND1	NM_053056	miR-155				0.54			
CCND2	NM_001759	miR-29a						0.76	
CCNJ	NM_001134375	miR-142-3p			0.81				
CCNL2	NM_030937	miR-29a						0.93	
CCNT2	NM_001241	miR-142-3p, miR-29b			0.84				0.83
CCNYL1	NM_001142300	miR-29b							0.83

Gene Symbol	Reference Sequence	miRNA(s) Predicted to Target the Gene	miR-125b P_{CT}	miR-126 P_{CT}	miR-142-3p P_{CT}	miR-155 P_{CT}	miR-223 P_{CT}	miR-29a P_{CT}	miR-29b P_{CT}
CD274	NM_014143	miR-155				0.57			
CD276	NM_001024736	miR-29a						0.82	
CD84	NM_001184879	miR-142-3p			0.73				
CD8A	NM_001145873	miR-142-3p			0.59				
CD99L2	NM_001184808	miR-155				0.55			
CDC42BPA	NM_003607	miR-29a						0.83	
CDC42SE1	NM_001038707	miR-125b	0.95						
CDC7	NM_001134419	miR-29a						0.76	
CDH5	NM_001795	miR-125b	0.97						
CDK19	NM_015076	miR-125b	0.61						
CDK6	NM_001145306	miR-29a						> 0.99	
CDKL2	NM_003948	miR-29a						0.75	
CDS2	NM_003818	miR-125b	0.73						
CEACAM1	NM_001024912	miR-29a						0.68	
CEBPB	NM_005194	miR-155				0.65			
CELF2	NM_001025076	miR-29a						0.8	
CELF6	NM_001172684	miR-29b							0.75
CELSR2	NM_001408	miR-125b	0.65						
CEP68	NM_015147	miR-29b							0.83
CEP97	NM_024548	miR-29a						0.88	
CFL2	NM_021914	miR-142-3p			0.68				
CHCHD7	NM_001011667	miR-155				0.55			
CHFR	NM_001161344	miR-29a						0.83	
CHIC2	NM_012110	miR-29a						0.79	
CHMP6	NM_024591	miR-29a						0.77	
CHRNE	NM_000080	miR-142-3p			0.77				
CHSY1	NM_014918	miR-29a						0.81	
CHURC1-FNTB	NM_001202558	miR-142-3p			0.83				
CIITA	NM_000246	miR-142-3p			0.74				
CILP2	NM_153221	miR-29a						0.83	
CLCC1	NM_001048210	miR-142-3p			0.56				
CLCN5	NM_000084	miR-142-3p, miR-29a			0.72			> 0.99	
CLDN1	NM_021101	miR-29a						0.82	
CLDN12	NM_001185072	miR-125b	0.52						
CLEC2L	NM_001080511	miR-29b							0.95
CLEC3A	NM_005752	miR-29a						0.84	
CLIC4	NM_013943	miR-142-3p			0.82				
CLMN	NM_024734	miR-29a						0.94	
CLSTN1	NM_001009566	miR-223					0.52		
CMPK1	NM_001136140	miR-29a						0.64	
CNNM1	NM_020348	miR-125b	0.66						
CNOT6	NM_015455	miR-29a						0.73	
CNOT8	NM_004779	miR-29b							0.53
CNR1	NM_001160226	miR-29b							0.83
COG4	NM_001195139	miR-142-3p			0.77				
COL11A1	NM_001190709	miR-29a						0.93	
COL15A1	NM_001855	miR-29b							0.92
COL16A1	NM_001856	miR-29a						0.76	
COL19A1	NM_001858	miR-29a						0.99	

Gene Symbol	Reference Sequence	miRNA(s) Predicted to Target the Gene	miR-125b P_{CT}	miR-126 P_{CT}	miR-142-3p P_{CT}	miR-155 P_{CT}	miR-223 P_{CT}	miR-29a P_{CT}	miR-29b P_{CT}
COL1A1	NM_000088	miR-29a						> 0.99	
COL1A2	NM_000089	miR-29a						0.96	
COL21A1	NM_030820	miR-29a						0.79	
COL22A1	NM_152888	miR-29a						0.9	
COL24A1	NM_152890	miR-142-3p, miR-29a			0.75			0.77	
COL2A1	NM_001844	miR-29b							0.9
COL3A1	NM_000090	miR-29b							0.98
COL4A1	NM_001845	miR-29b							0.99
COL4A2	NM_001846	miR-29a						0.83	
COL4A3	NM_000091	miR-29a						0.97	
COL4A4	NM_000092	miR-29a						0.97	
COL4A5	NM_000495	miR-29b							
COL4A6	NM_001847	miR-29b							0.92
COL5A1	NM_000093	miR-29a						> 0.99	
COL5A2	NM_000393	miR-29a						> 0.99	
COL5A3	NM_015719	miR-29a						> 0.99	
COL6A2	NM_001849	miR-29a						0.83	
COL6A3	NM_004369	miR-29a						0.92	
COL6A6	NM_001102608	miR-29a						0.6	
COL7A1	NM_000094	miR-29b							0.97
COMMD2	NM_016094	miR-29a						0.87	
COPG	NM_016128	miR-142-3p			0.82				
COPS3	NM_001199125	miR-155				0.55			
CORO2B	NM_001190456	miR-125b	0.56						
CORO6	NM_032854	miR-29a						0.76	
CPEB2	NM_001177381	miR-142-3p			0.78				
CPM	NM_001005502	miR-125b	0.64						
CPS1	NM_001122633	miR-29a						0.79	
CPSF6	NM_007007	miR-125b	0.97						
CPSF7	NM_001136040	miR-29a						0.82	
CPT2	NM_000098	miR-29a						0.83	
CRAMP1L	NM_020825	miR-125b	0.53						
CREB1	NM_004379	miR-155				0.58			
CREB5	NM_001011666	miR-29a						0.83	
CREBZF	NM_001039618	miR-223					0.52		
CRISPLD1	NM_031461	miR-29a						0.83	
CRK	NM_005206	miR-126, miR-142-3p		0.55	0.81				
CRTAM	NM_019604	miR-142-3p			0.83				
CSDA	NM_001145426	miR-29a						0.9	
CSF1R	NM_005211	miR-155				0.55			
CSF2RB	NM_000395	miR-155				0.52			
CSNK1G1	NM_022048	miR-223, miR-29a					0.52	0.67	
CSNK2A1	NM_001895	miR-125b	0.79						
CSPG4	NM_001897	miR-29a						0.68	
CSRNP2	NM_030809	miR-29a						0.83	
CTC1	NM_025099	miR-29a						0.92	
CTNNBIP1	NM_001012329	miR-29a						0.83	
CTNND1	NM_001085458	miR-142-3p, miR-29a			0.82			0.83	
CTSL2	NM_001201575	miR-223					0.58		

**Gene Symbol**	**Reference Sequence**	**miRNA(s) Predicted to Target the Gene**	**miR-125b P_{CT}**	**miR-126 P_{CT}**	**miR-142-3p P_{CT}**	**miR-155 P_{CT}**	**miR-223 P_{CT}**	**miR-29a P_{CT}**	**miR-29b P_{CT}**
CTTN	NM_005231	miR-142-3p			0.86				
CUEDC1	NM_017949	miR-29a						0.79	
CUL5	NM_003478	miR-142-3p			0.64				
CUX1	NM_001202543	miR-142-3p, miR-155			0.75	0.58			
CX3CL1	NM_002996	miR-29a						0.94	
CYCS	NM_018947	miR-29b							0.8
CYTH1	NM_004762	miR-125b	0.96						
CYTH3	NM_004227	miR-29a						0.78	
DAAM2	NM_001201427	miR-29a						0.82	
DAB2IP	NM_032552	miR-29a						0.83	
DBT	NM_001918	miR-29a						0.83	
DCAF12	NM_015397	miR-29a						0.94	
DCAF5	NM_003861	miR-29b							0.83
DCAF7	NM_005828	miR-29a						0.83	
DCLK3	NM_033403	miR-29a						0.81	
DCP2	NM_001242377	miR-29a						0.86	
DCUN1D3	NM_173475	miR-155				0.55			
DCUN1D4	NM_001040402	miR-142-3p, miR-29a			0.86			0.9	
DCUN1D5	NM_032299	miR-29a						0.7	
DCX	NM_000555	miR-29a						0.84	
DDX39B	NM_004640	miR-142-3p			0.82				
DDX3X	NM_001193416	miR-29a						0.89	
DDX42	NM_007372	miR-125b	0.67						
DDX46	NM_014829	miR-29a						0.83	
DEDD	NM_001039711	miR-29a						0.81	
DEF8	NM_001242816	miR-29a						0.8	
DENND1B	NM_001195215	miR-29a						0.83	
DENND5B	NM_144973	miR-223					0.64		
DGKB	NM_004080	miR-155				0.55			
DGKD	NM_003648	miR-29a						0.93	
DHTKD1	NM_018706	miR-29a						0.79	
DIABLO	NM_019887	miR-29a						0.61	
DICER1	NM_001195573	miR-29a						0.92	
DIO2	NM_000793	miR-29a						0.9	
DIP2B	NM_173602	miR-29a						0.8	
DIP2C	NM_014974	miR-126, miR-29a		0.55				0.8	
DIRC2	NM_032839	miR-142-3p			0.83				
DLG2	NM_001142699	miR-29a						> 0.99	
DLGAP1	NM_001003809	miR-29b							0.68
DLGAP2	NM_004745	miR-29b							0.99
DMTF1	NM_001142326	miR-142-3p			0.64				
DNAJB1	NM_006145	miR-155				0.54			
DNAJB11	NM_016306	miR-29a						0.78	
DNAL1	NM_001201366	miR-125b, miR-29a	0.57					0.97	
DNM3	NM_001136127	miR-29a						0.72	
DNMT3A	NM_022552	miR-29a						0.8	
DNMT3B	NM_001207055	miR-29a						0.94	
DOK6	NM_152721	miR-155				0.58			
DOLPP1	NM_001135917	miR-29a						0.82	

**Gene Symbol**	**Reference Sequence**	**miRNA(s) Predicted to Target the Gene**	**miR-125b P_{CT}**	**miR-126 P_{CT}**	**miR-142-3p P_{CT}**	**miR-155 P_{CT}**	**miR-223 P_{CT}**	**miR-29a P_{CT}**	**miR-29b P_{CT}**
DOT1L	NM_032482	miR-29b							
DPF1	NM_001135155	miR-29a						0.63	
DPP4	NM_001935	miR-29a						0.9	
DPP9	NM_139159	miR-125b	0.9						
DPYSL2	NM_001197293	miR-29a						0.81	
DPYSL5	NM_020134	miR-29a						0.92	
DRAM2	NM_178454	miR-125b	0.88						
DRD1	NM_000794	miR-29a						0.83	
DRP2	NM_001171184	miR-29a						0.83	
DSC2	NM_004949	miR-29a						0.63	
DSEL	NM_032160	miR-223					0.5		
DTWD2	NM_173666	miR-29a						0.91	
DTX4	NM_015177	miR-29a						0.91	
DUSP2	NM_004418	miR-29a						0.83	
DVL3	NM_004423	miR-125b	0.82						
DYNC1LI2	NM_006141	miR-142-3p			0.76				
DYNLT1	NM_006519	miR-29b							0.93
DYNLT3	NM_006520	miR-125b	0.63						
E2F2	NM_004091	miR-155				0.68			
E2F7	NM_203394	miR-29a						0.94	
EDC3	NM_001142443	miR-29a						0.96	
EDEM3	NM_025191	miR-142-3p			0.82				
EFHD2	NM_024329	miR-126		0.56					
EFNA5	NM_001962	miR-29a						0.9	
EGFL7	NM_016215	miR-126		0.55					
EGR2	NM_000399	miR-142-3p			0.77				
EHD1	NM_006795	miR-155				0.55			
EHD2	NM_014601	miR-29a						0.91	
EHF	NM_001206615	miR-142-3p, miR-155			0.83	0.55			
EIF2C2	NM_001164623	miR-125b	0.68						
EIF2S2	NM_003908	miR-29b							0.8
EIF4E3	NM_001134649	miR-223					0.61		
EIF5A2	NM_020390	miR-125b	0.92						
ELF2	NM_006874	miR-29a						0.89	
ELK4	NM_001973	miR-223					0.57		
ELL2	NM_012081	miR-223, miR-29a					0.52	0.76	
ELMO2	NM_133171	miR-29a						0.78	
ELN	NM_000501	miR-29b							
ELOVL4	NM_022726	miR-29a						0.77	
EML4	NM_001145076	miR-142-3p, miR-29a			0.96			0.83	
EML5	NM_183387	miR-29b							0.83
EML6	NM_001039753	miR-29a						0.94	
EMP1	NM_001423	miR-29a						0.94	
EMP2	NM_001424	miR-155, miR-29a				0.55		0.56	
EN1	NM_001426	miR-29a						0.83	
ENAH	NM_001008493	miR-29a						0.83	
ENHO	NM_198573	miR-29a						0.83	
ENTPD1	NM_001098175	miR-125b	0.6						
ENTPD4	NM_001128930	miR-125b	0.92						

Gene Symbol	Reference Sequence	miRNA(s) Predicted to Target the Gene	miR-125b P_{CT}	miR-126 P_{CT}	miR-142-3p P_{CT}	miR-155 P_{CT}	miR-223 P_{CT}	miR-29a P_{CT}	miR-29b P_{CT}
ENTPD7	NM_020354	miR-155, miR-29b				0.54			0.94
EPHA10	NM_001099439	miR-29b							0.94
EPHB3	NM_004443	miR-29a						0.62	
EPN1	NM_001130071	miR-142-3p			0.78				
EPS15	NM_001159969	miR-29a						0.79	
ERC1	NM_178039	miR-142-3p, miR-29b			0.85				0.94
ERCC6	NM_000124	miR-29a						0.83	
ERG	NM_001136154	miR-142-3p			0.83				
ERLIN2	NM_007175	miR-29a						0.92	
ERP44	NM_015051	miR-29b							0.94
ETNK2	NM_018208	miR-155				0.55			
ETS1	NM_001143820	miR-155				0.64			
ETV4	NM_001079675	miR-29a						0.82	
ETV6	NM_001987	miR-29a						0.93	
EYA2	NM_005244	miR-155				0.52			
EYA3	NM_001990	miR-223					0.67		
F11R	NM_016946	miR-29a						0.94	
F3	NM_001178096	miR-223					0.52		
FAM105B	NM_138348	miR-29a						0.59	
FAM114A1	NM_138389	miR-142-3p			0.97				
FAM116A	NM_152678	miR-29a						0.83	
FAM118A	NM_001104595	miR-125b	0.86						
FAM123B	NM_152424	miR-29a						0.89	
FAM124A	NM_001242312	miR-29a						0.81	
FAM126B	NM_173822	miR-29b							0.78
FAM127A	NM_001078171	miR-142-3p			0.72				
FAM131B	NM_001031690	miR-29a						0.75	
FAM134A	NM_024293	miR-125b	0.97						
FAM136A	NM_032822	miR-29a						0.88	
FAM13B	NM_001101800	miR-29a						0.71	
FAM167A	NM_053279	miR-29a						0.83	
FAM168B	NM_001009993	miR-29a						0.96	
FAM174B	NM_207446	miR-125b	0.66						
FAM176A	NM_001135032	miR-29a						0.57	
FAM176B	NM_018166	miR-29a						0.72	
FAM190B	NM_018999	miR-155, miR-29a				0.5		0.54	
FAM193B	NM_001190946	miR-29a						0.92	
FAM199X	NM_207318	miR-223					0.59		
FAM46A	NM_017633	miR-125b	0.54						
FAM53C	NM_001135647	miR-29b							0.68
FAM57B	NM_031478	miR-29a						0.92	
FAM65B	NM_014722	miR-125b, miR-29a	0.87					0.82	
FAM70A	NM_001104544	miR-29b							0.58
FAT4	NM_024582	miR-125b	0.65						
FBN1	NM_000138	miR-29a						> 0.99	
FBRS	NM_001105079	miR-29a						0.83	
FBXL18	NM_024963	miR-29a						0.94	
FBXL20	NM_001184906	miR-29a						0.83	
FBXO21	NM_015002	miR-142-3p			0.82				

Gene Symbol	Reference Sequence	miRNA(s) Predicted to Target the Gene	miR-125b P_{CT}	miR-126 P_{CT}	miR-142-3p P_{CT}	miR-155 P_{CT}	miR-223 P_{CT}	miR-29a P_{CT}	miR-29b P_{CT}
FBXO33	NM_203301	miR-126		0.55					
FBXO41	NM_001080410	miR-29a						0.61	
FBXO45	NM_001105573	miR-142-3p			0.77				
FBXW2	NM_012164	miR-29a						0.99	
FBXW7	NM_001013415	miR-223, miR-29a					0.87	0.89	
FBXW9	NM_032301	miR-29b							0.83
FCHSD1	NM_033449	miR-29b							0.87
FEM1B	NM_015322	miR-29a						0.65	
FGA	NM_000508	miR-29a						0.92	
FGD4	NM_139241	miR-29a						0.94	
FGF7	NM_002009	miR-155				0.85			
FGF9	NM_002010	miR-155				0.59			
FIGN	NM_018086	miR-29a						0.82	
FIGNL2	NM_001013690	miR-223					0.52		
FKBP4	NM_002014	miR-29a						0.75	
FLJ36031	NM_175884	miR-125b	0.83						
FLVCR1	NM_014053	miR-142-3p			0.82				
FMN1	NM_001103184	miR-29a						0.94	
FMNL2	NM_052905	miR-142-3p			0.7				
FNDC3A	NM_001079673	miR-142-3p			0.81				
FNDC3B	NM_001135095	miR-125b	0.91						
FNDC5	NM_001171940	miR-29a						0.67	
FNTB	NM_002028	miR-142-3p			0.83				
FOS	NM_005252	miR-29a						0.71	
FOXJ2	NM_018416	miR-29a						0.86	
FOXK1	NM_001037165	miR-155				0.5			
FOXN3	NM_001085471	miR-29a						0.83	
FOXO1	NM_002015	miR-142-3p			0.74				
FOXO3	NM_001455	miR-29b							0.83
FOXO4	NM_001170931	miR-142-3p, miR-29a			0.85			0.69	
FRAS1	NM_025074	miR-29b							0.83
FRAT2	NM_012083	miR-29a						0.93	
FREM1	NM_001177704	miR-29a						0.77	
FREM2	NM_207361	miR-29a						0.97	
FRMD4A	NM_018027	miR-223, miR-29a					0.61	0.94	
FSTL1	NM_007085	miR-29a						0.94	
FZD4	NM_012193	miR-29a						0.82	
FZD5	NM_003468	miR-155				0.57			
G6PC	NM_000151	miR-29a						0.83	
GAB1	NM_002039	miR-142-3p, miR-29a			0.83			0.83	
GAB3	NM_001081573	miR-155				0.55			
GALNT7	NM_017423	miR-125b	0.9						
GAS7	NM_001130831	miR-29a						0.92	
GCC2	NM_181453	miR-29a						0.63	
GCSH	NM_004483	miR-29a						0.74	
GDF6	NM_001001557	miR-155				0.55			
GDNF	NM_000514	miR-125b	0.68						
GFAP	NM_001242376	miR-142-3p			0.66				
GFI1	NM_001127215	miR-142-3p			0.8				

Gene Symbol	Reference Sequence	miRNA(s) Predicted to Target the Gene	miR-125b P_{CT}	miR-126 P_{CT}	miR-142-3p P_{CT}	miR-155 P_{CT}	miR-223 P_{CT}	miR-29a P_{CT}	miR-29b P_{CT}
GGA1	NM_001001560	miR-29a						0.81	
GGCT	NM_001199815	miR-29a						0.65	
GLIS2	NM_032575	miR-29a						0.89	
GLIS3	NM_001042413	miR-29a						0.78	
GLS	NM_014905	miR-125b	0.71						
GLTP	NM_016433	miR-125b	0.63						
GMFB	NM_004124	miR-29a						0.76	
GNA13	NM_006572	miR-126, miR-29b		0.56					0.74
GNAQ	NM_002072	miR-142-3p			0.85				
GNB2	NM_005273	miR-142-3p			0.84				
GNB4	NM_021629	miR-29a						0.83	
GNG12	NM_018841	miR-29b							0.91
GNG2	NM_053064	miR-29a						0.83	
GNS	NM_002076	miR-29a						0.73	
GOLGA1	NM_002077	miR-142-3p			0.69				
GPAM	NM_020918	miR-29a						0.79	
GPATCH2	NM_018040	miR-29a						0.92	
GPATCH8	NM_001002909	miR-125b	0.56						
GPCPD1	NM_019593	miR-142-3p, miR-29a			0.71			0.93	
GPI	NM_000175	miR-29a						0.81	
GPR153	NM_207370	miR-125b	0.56						
GPR85	NM_001146265	miR-142-3p, miR-29b			0.62				0.94
GPX7	NM_015696	miR-29b							0.82
GRAMD4	NM_015124	miR-223					0.52		
GRHL1	NM_198182	miR-125b	0.71						
GRIP1	NM_001178074	miR-29a						0.88	
GRM4	NM_000841	miR-29a						0.83	
GRPEL2	NM_152407	miR-29b							0.68
GRSF1	NM_001098477	miR-125b	0.91						
GSK3B	NM_001146156	miR-155, miR-29a				0.55		0.83	
GSTA4	NM_001512	miR-29a						0.78	
GTF2A1	NM_015859	miR-142-3p			0.75				
GTPBP2	NM_019096	miR-29a						0.94	
GXYLT2	NM_001080393	miR-29a						0.54	
H2AFY	NM_001040158	miR-29a						0.73	
HAPLN1	NM_001884	miR-125b, miR-29a	0.89					0.8	
HAPLN2	NM_021817	miR-155				0.54			
HAS3	NM_001199280	miR-29b							0.84
HBEGF	NM_001945	miR-29a						0.89	
HBP1	NM_012257	miR-29b							0.89
HDAC4	NM_006037	miR-29b							0.83
HDGF	NM_001126050	miR-29a						0.93	
HECTD1	NM_015382	miR-142-3p			0.8				
HELZ	NM_014877	miR-29a						0.83	
HGS	NM_004712	miR-142-3p			0.74				
HHEX	NM_002729	miR-223					0.51		
HIATL1	NM_032558	miR-125b, miR-29a	0.5					0.83	
HIF3A	NM_022462	miR-29a						> 0.99	
HINFP	NM_015517	miR-125b	0.67						

Gene Symbol	Reference Sequence	miRNA(s) Predicted to Target the Gene	miR-125b P_{CT}	miR-126 P_{CT}	miR-142-3p P_{CT}	miR-155 P_{CT}	miR-223 P_{CT}	miR-29a P_{CT}	miR-29b P_{CT}
HIP1	NM_005338	miR-142-3p, miR-29a			0.56			0.77	
HIPK1	NM_181358	miR-142-3p			0.52				
HIVEP2	NM_006734	miR-155				0.57			
HLF	NM_002126	miR-223, miR-29a					0.83	0.92	
HMCN1	NM_031935	miR-29b							0.83
HMGA1	NM_002131	miR-142-3p			0.8				
HMGA2	NM_003483	miR-142-3p			0.64				
HMGB1	NM_002128	miR-142-3p			0.83				
HMGCR	NM_000859	miR-29a						0.66	
HMGCS1	NM_001098272	miR-29a						0.79	
HMGN3	NM_001201362	miR-29a						0.63	
HN1	NM_001002032	miR-29a						0.82	
HNF4G	NM_004133	miR-29a						0.83	
HNRNPA3	NM_194247	miR-155				0.56			
HNRNPF	NM_001098204	miR-29b							0.87
HNRNPUL1	NM_007040	miR-29a						0.58	
HOMEZ	NM_020834	miR-125b	0.87						
HOOK3	NM_032410	miR-29a						> 0.99	
HOXB3	NM_002146	miR-125b	0.88						
HOXC13	NM_017410	miR-29a						0.71	
HPCAL4	NM_016257	miR-29b							0.91
HS3ST3B1	NM_006041	miR-29a						0.7	
HSPE1-MOBKL3	NM_001202485	miR-142-3p			0.83				
HSPG2	NM_005529	miR-29a						0.92	
HTR7	NM_000872	miR-29a						0.71	
ICOS	NM_012092	miR-29a						0.8	
IDE	NM_001165946	miR-29a						0.78	
IER3	NM_003897	miR-142-3p			0.75				
IFFO1	NM_001039670	miR-29a						0.83	
IFFO2	NM_001136265	miR-29a						0.83	
IFI30	NM_006332	miR-29a						0.92	
IGF1	NM_000618	miR-29a						> 0.99	
IGF2BP1	NM_001160423	miR-29a						0.73	
IGSF11	NM_001015887	miR-125b	0.57						
IKZF1	NM_001220765	miR-142-3p			0.68				
IKZF4	NM_022465	miR-125b, miR-29a	0.94					0.74	
IL17RD	NM_017563	miR-29a						0.83	
IL1RAP	NM_001167928	miR-29a						0.51	
IL6R	NM_000565	miR-125b	0.78						
IL6ST	NM_001190981	miR-142-3p, miR-155, miR-223			0.77	0.62	0.51		
ILDR2	NM_199351	miR-29a						0.83	
IMPDH1	NM_000883	miR-29b							0.8
INA	NM_032727	miR-29b							0.91
INADL	NM_176877	miR-155				0.58			
INO80D	NM_017759	miR-125b, miR-29a	0.99					0.78	
INPP5A	NM_005539	miR-142-3p			0.83				
INPP5B	NM_005540	miR-223					0.52		
INPP5D	NM_001017915	miR-155				0.58			

Gene Symbol	Reference Sequence	miRNA(s) Predicted to Target the Gene	miR-125b P_{CT}	miR-126 P_{CT}	miR-142-3p P_{CT}	miR-155 P_{CT}	miR-223 P_{CT}	miR-29a P_{CT}	miR-29b P_{CT}
INPP5F	NM_014937	miR-142-3p			0.57				
INSIG1	NM_005542	miR-29a						0.83	
INTS7	NM_001199809	miR-125b	0.83						
IPMK	NM_152230	miR-125b, miR-142-3p	0.59		0.82				
IPO7	NM_006391	miR-142-3p			0.76				
IQCJ-SCHIP1	NM_001197113	miR-29b							0.88
IRAK1	NM_001025242	miR-142-3p			0.87				
IREB2	NM_004136	miR-29a						0.94	
IRF2BP2	NM_001077397	miR-155				0.55			
IRGQ	NM_001007561	miR-29a						0.81	
IRS1	NM_005544	miR-126, miR-29b		0.56					0.54
ISG20L2	NM_030980	miR-29a						0.92	
ISL1	NM_002202	miR-29a						0.81	
ISLR2	NM_001130136	miR-29a						0.83	
ITGA11	NM_001004439	miR-29a						0.87	
ITGA6	NM_000210	miR-29b							0.85
ITGAV	NM_001144999	miR-142-3p			0.87				
ITGB1	NM_002211	miR-29a						0.81	
ITGB8	NM_002214	miR-142-3p			0.99				
ITPKB	NM_002221	miR-142-3p			0.77				
ITPR3	NM_002224	miR-142-3p			0.76				
JARID2	NM_004973	miR-155, miR-29a				0.74		0.78	
JAZF1	NM_175061	miR-29a						0.77	
JHDM1D	NM_030647	miR-155				0.6			
JMJD1C	NM_004241	miR-142-3p			0.7				
JMY	NM_152405	miR-29a						0.83	
JOSD1	NM_014876	miR-29a						0.83	
KAT2A	NM_021078	miR-155				0.54			
KAT2B	NM_003884	miR-142-3p			0.73				
KAT7	NM_001199155	miR-142-3p			0.81				
KBTBD8	NM_032505	miR-29a						0.68	
KCNIP2	NM_014591	miR-29a						0.83	
KCNJ12	NM_021012	miR-29b							0.83
KCNK10	NM_021161	miR-125b, miR-29a	0.98					0.83	
KCNMA1	NM_001014797	miR-29a						0.54	
KCTD15	NM_001129994	miR-29a						0.83	
KCTD20	NM_173562	miR-29a						0.97	
KCTD21	NM_001029859	miR-125b	0.69						
KCTD3	NM_016121	miR-29a						0.92	
KCTD5	NM_018992	miR-29a						0.92	
KCTD7	NM_001167961	miR-155				0.58			
KDELR2	NM_001100603	miR-142-3p			0.7				
KDM2A	NM_012308	miR-29a						0.78	
KDM4B	NM_015015	miR-29a						0.94	
KDM5A	NM_001042603	miR-29a						0.76	
KDM5B	NM_006618	miR-155, miR-29b				0.61			0.99
KDM5C	NM_001146702	miR-29a						0.83	
KDM6A	NM_021140	miR-142-3p			0.81				
KDM6B	NM_001080424	miR-29a						0.83	

Gene Symbol	Reference Sequence	miRNA(s) Predicted to Target the Gene	miR-125b P_{CT}	miR-126 P_{CT}	miR-142-3p P_{CT}	miR-155 P_{CT}	miR-223 P_{CT}	miR-29a P_{CT}	miR-29b P_{CT}
KIAA0355	NM_014686	miR-29a						0.83	
KIAA0895	NM_001100425	miR-29a						0.9	
KIAA0895L	NM_001040715	miR-29a						0.82	
KIAA1024	NM_015206	miR-29a						> 0.99	
KIAA1199	NM_018689	miR-29a						0.94	
KIAA1267	NM_001193465	miR-155				0.55			
KIAA1274	NM_014431	miR-155				0.55			
KIAA1432	NM_001206557	miR-29a						0.8	
KIAA1462	NM_020848	miR-155				0.52			
KIAA1549	NM_001164665	miR-29a						0.91	
KIAA1644	NM_001099294	miR-125b, miR-29a	0.72					0.85	
KIAA2018	NM_001009899	miR-29a						0.82	
KIAA2022	NM_001008537	miR-29b							0.98
KIF26A	NM_015656	miR-29a						0.92	
KIF26B	NM_018012	miR-29a						0.9	
KIF3B	NM_004798	miR-29a						0.82	
KIF5B	NM_004521	miR-142-3p, miR-29a			0.76			0.65	
KIFC2	NM_145754	miR-29a						0.92	
KIRREL	NM_018240	miR-29a						0.83	
KLC2	NM_001134774	miR-125b	0.8						
KLF12	NM_007249	miR-29a						0.87	
KLF13	NM_015995	miR-142-3p			0.77				
KLF3	NM_016531	miR-155				0.81			
KLF4	NM_004235	miR-29a						0.81	
KLHDC10	NM_014997	miR-29a						0.79	
KLHDC3	NM_001242872	miR-29a						0.83	
KLHDC5	NM_020782	miR-29a						0.56	
KLHL25	NM_022480	miR-29a						0.8	
KLHL28	NM_017658	miR-29a						0.94	
KLHL8	NM_020803	miR-29a						0.82	
KLHL9	NM_018847	miR-29a						0.81	
KPNA1	NM_002264	miR-155, miR-29a				0.74		0.87	
KPNA4	NM_002268	miR-155, miR-29a				0.55		0.83	
KPNA6	NM_012316	miR-125b	0.9						
KSR1	NM_014238	miR-155				0.66			
KY	NM_178554	miR-29a						0.94	
L3MBTL4	NM_173464	miR-29a						0.77	
LACTB	NM_032857	miR-125b	0.94						
LAMC1	NM_002293	miR-29b							0.83
LAMC2	NM_005562	miR-29a						0.57	
LARP4	NM_001170803	miR-29a						0.94	
LARP4B	NM_015155	miR-29a						0.71	
LASP1	NM_006148	miR-29a						0.99	
LCOR	NM_001170765	miR-142-3p			0.98				
LDLRAP1	NM_015627	miR-29a						0.5	
LDOC1L	NM_032287	miR-29a						0.83	
LEP	NM_000230	miR-29a						0.69	
LGI2	NM_018176	miR-125b, miR-29a	0.71					0.52	
LIF	NM_002309	miR-29a						0.83	

Gene Symbol	Reference Sequence	miRNA(s) Predicted to Target the Gene	miR-125b P_{CT}	miR-126 P_{CT}	miR-142-3p P_{CT}	miR-155 P_{CT}	miR-223 P_{CT}	miR-29a P_{CT}	miR-29b P_{CT}
LIFR	NM_001127671	miR-125b, miR-142-3p	0.91		0.53				
LIMS1	NM_001193482	miR-29a						0.83	
LLGL2	NM_001031803	miR-142-3p			0.83				
LMLN	NM_001136049	miR-29a						0.83	
LMO3	NM_001001395	miR-142-3p			0.73				
LNPEP	NM_005575	miR-125b, miR-29a	0.92					0.93	
LOC100507421	NM_001195278	miR-29a						0.93	
LOC147670	NM_001193628	miR-29a						0.92	
LOX	NM_001178102	miR-29b							
LOXL2	NM_002318	miR-29a						0.75	
LOXL4	NM_032211	miR-29a						0.81	
LPGAT1	NM_014873	miR-29a						0.71	
LPL	NM_000237	miR-29a						0.86	
LPP	NM_001167671	miR-142-3p			0.82				
LPPR4	NM_001166252	miR-29a						0.75	
LPPR5	NM_001010861	miR-29a						0.83	
LRP1B	NM_018557	miR-142-3p			0.69				
LRP6	NM_002336	miR-126, miR-29b		0.56					0.97
LRRC1	NM_018214	miR-142-3p			0.8				
LRRC32	NM_001128922	miR-142-3p			0.83				
LRRC58	NM_001099678	miR-29b							0.88
LRRC59	NM_018509	miR-142-3p, miR-155			0.88	0.51			
LRRC68	NM_019121	miR-142-3p			0.93				
LRRC8B	NM_001134476	miR-125b	0.97						
LSM11	NM_173491	miR-29a						0.65	
LSM14B	NM_144703	miR-29a						0.83	
LTBR	NM_002342	miR-29a						0.76	
LUZP1	NM_001142546	miR-29a						0.82	
LYPLA1	NM_006330	miR-29a						0.72	
LYSMD1	NM_001136543	miR-29a						0.92	
MAF	NM_001031804	miR-125b	0.68						
MAFB	NM_005461	miR-155, miR-29b				0.55			0.83
MAFG	NM_002359	miR-29a						0.84	
MALT1	NM_006785	miR-142-3p			0.6				
MAN1B1	NM_016219	miR-125b	0.95						
MAP2K4	NM_003010	miR-29a						0.54	
MAP2K6	NM_002758	miR-29b							0.84
MAP2K7	NM_145185	miR-125b	0.93						
MAP3K1	NM_005921	miR-125b	0.5						
MAP3K11	NM_002419	miR-142-3p			0.83				
MAP3K13	NM_001242314	miR-155				0.55			
MAP3K14	NM_003954	miR-155				0.62			
MAP3K2	NM_006609	miR-29a						0.75	
MAP3K3	NM_002401	miR-125b	0.62						
MAP4K3	NM_003618	miR-142-3p			0.54				
MAP4K4	NM_001242559	miR-155, miR-29a				0.5		> 0.99	
MAP6D1	NM_024871	miR-29a						0.92	
MAPK10	NM_002753	miR-29b							0.77
MAPKBP1	NM_001128608	miR-29a						0.83	

Gene Symbol	Reference Sequence	miRNA(s) Predicted to Target the Gene	miR-125b P_{CT}	miR-126 P_{CT}	miR-142-3p P_{CT}	miR-155 P_{CT}	miR-223 P_{CT}	miR-29a P_{CT}	miR-29b P_{CT}
MAPRE1	NM_012325	miR-29a						0.72	
MAPRE2	NM_001143826	miR-29a						0.82	
MARCH1	NM_001166373	miR-142-3p, miR-29a			0.76			0.94	
MARCKS	NM_002356	miR-142-3p			0.53				
MARK2	NM_001039469	miR-155				0.55			
MARK3	NM_001128918	miR-142-3p, miR-29a			0.66			0.73	
MAST4	NM_001164664	miR-29a						0.76	
MAT1A	NM_000429	miR-29a						0.51	
MAU2	NM_015329	miR-29a						0.8	
MAZ	NM_001042539	miR-29a						0.7	
MBD6	NM_052897	miR-142-3p			0.83				
MBLAC2	NM_203406	miR-29a						0.79	
MBNL3	NM_001170701	miR-223					0.65		
MBTD1	NM_017643	miR-29a						0.84	
MCL1	NM_001197320	miR-29a						0.92	
MED12L	NM_053002	miR-29a						0.82	
MED26	NM_004831	miR-29a						0.93	
MEGF6	NM_001409	miR-29a						0.92	
MEGF8	NM_001410	miR-125b	0.67						
MEGF9	NM_001080497	miR-125b	0.89						
MEST	NM_002402	miR-29a						0.83	
METAP2	NM_006838	miR-29a						0.78	
METTL21D	NM_001040662	miR-29a						0.78	
MEX3B	NM_032246	miR-29a						0.85	
MFAP2	NM_001135247	miR-29b							0.77
MFAP3	NM_001135037	miR-29a						0.94	
MGA	NM_001080541	miR-29a						0.91	
MGAT4A	NM_012214	miR-125b, miR-142-3p, miR-29b	0.74		0.82				0.79
MIB1	NM_020774	miR-125b	0.87						
MID2	NM_012216	miR-155				0.55			
MIDN	NM_177401	miR-155				0.69			
MIER3	NM_152622	miR-29a						0.6	
MLF1	NM_001130156	miR-29a						0.83	
MLIP	NM_138569	miR-29a						0.61	
MLLT1	NM_005934	miR-142-3p			0.67				
MLLT11	NM_006818	miR-29a						0.83	
MLXIP	NM_014938	miR-29a						0.83	
MMD	NM_012329	miR-142-3p			0.63				
MMGT1	NM_173470	miR-142-3p			0.73				
MMP16	NM_005941	miR-223					0.59		
MMP2	NM_001127891	miR-29a						0.83	
MMP24	NM_006690	miR-29a						0.78	
MOBKL3	NM_001100819	miR-142-3p			0.83				
MOG	NM_001008228	miR-29a						0.88	
MON1A	NM_001142501	miR-155				0.55			
MORF4L1	NM_006791	miR-142-3p			0.54				
MORF4L2	NM_001142418	miR-142-3p, miR-29a			0.85			0.82	
MPEG1	NM_001039396	miR-155				0.59			
MRE11A	NM_005590	miR-29a						0.69	

Gene Symbol	Reference Sequence	miRNA(s) Predicted to Target the Gene	miR-125b P_{CT}	miR-126 P_{CT}	miR-142-3p P_{CT}	miR-155 P_{CT}	miR-223 P_{CT}	miR-29a P_{CT}	miR-29b P_{CT}
MRFAP1	NM_033296	miR-142-3p			0.67				
MSL1	NM_001012241	miR-29a						0.87	
MSRB3	NM_001031679	miR-125b	0.87						
MSTN	NM_005259	miR-29a						0.69	
MTCH1	NM_014341	miR-142-3p			0.82				
MTCP1	NM_001018025	miR-29a						0.81	
MTF1	NM_005955	miR-125b	0.97						
MTMR4	NM_004687	miR-29a						0.57	
MTMR9	NM_015458	miR-142-3p			0.56				
MTSS1L	NM_138383	miR-29a						0.83	
MTX3	NM_001010891	miR-29a						0.8	
MXD1	NM_001202513	miR-29a						0.97	
MYBL2	NM_002466	miR-29a						0.83	
MYCN	NM_005378	miR-29a						0.72	
MYH10	NM_005964	miR-142-3p, miR-223			0.85		0.51		
MYH9	NM_002473	miR-142-3p			0.62				
MYLK	NM_053025	miR-142-3p, miR-155			0.83	0.58			
MYO10	NM_012334	miR-155				0.65			
MYO16	NM_001198950	miR-29a						0.83	
MYO1D	NM_015194	miR-155				0.63			
MYO5A	NM_000259	miR-142-3p			0.85				
N4BP2	NM_018177	miR-29a						0.83	
N4BP2L1	NM_001079691	miR-29a						0.83	
NAA40	NM_024771	miR-29b							0.83
NAA50	NM_025146	miR-155, miR-223				0.52	0.51		
NANOS1	NM_199461	miR-29a						0.83	
NANP	NM_152667	miR-29a						0.69	
NAP1L3	NM_004538	miR-29a						0.74	
NAPB	NM_022080	miR-29a						0.91	
NASP	NM_001195193	miR-29a						0.91	
NAT15	NM_001083600	miR-29a						0.93	
NAV1	NM_001167738	miR-29b							
NAV2	NM_001111018	miR-29a						0.95	
NAV3	NM_014903	miR-29a						0.98	
NCAN	NM_004386	miR-125b	0.96						
NCKAP5	NM_207363	miR-29a						0.5	
NCKAP5L	NM_001037806	miR-29b							0.96
NCLN	NM_020170	miR-125b	0.68						
NCOA3	NM_001174087	miR-29a						0.81	
NCOA4	NM_001145260	miR-29a						0.83	
NCOR2	NM_001077261	miR-125b, miR-29a	0.88					0.83	
NCS1	NM_001128826	miR-223					0.58		
NDN	NM_002487	miR-29a						0.71	
NDST1	NM_001543	miR-29a						0.92	
NEBL	NM_001173484	miR-29b							0.83
NEO1	NM_001172623	miR-125b	0.6						
NFASC	NM_001005388	miR-155, miR-223				0.55	0.52		
NFAT5	NM_001113178	miR-29a						0.97	
NFATC3	NM_004555	miR-29a						0.84	

Gene Symbol	Reference Sequence	miRNA(s) Predicted to Target the Gene	miR-125b P_{CT}	miR-126 P_{CT}	miR-142-3p P_{CT}	miR-155 P_{CT}	miR-223 P_{CT}	miR-29a P_{CT}	miR-29b P_{CT}
NFATC4	NM_001136022	miR-29a						0.83	
NFIA	NM_001134673	miR-155, miR-223, miR-29a				0.63	0.67	> 0.99	
NFIB	NM_001190737	miR-142-3p, miR-29b			0.55				0.69
NFIX	NM_002501	miR-29a						0.83	
NGEF	NM_001114090	miR-155				0.55			
NHSL1	NM_001144060	miR-142-3p			0.74				
NID1	NM_002508	miR-29a						0.78	
NID2	NM_007361	miR-29a						0.8	
NIPA1	NM_001142275	miR-125b	0.96						
NKAIN1	NM_024522	miR-29a						0.83	
NKIRAS2	NM_001001349	miR-29a						0.94	
NKRF	NM_001173487	miR-29b							0.67
NKTR	NM_005385	miR-29a						0.83	
NKX2-3	NM_145285	miR-142-3p			0.74				
NLGN3	NM_001166660	miR-29a						0.73	
NOTCH2	NM_024408	miR-29a						0.83	
NPAS3	NM_001164749	miR-29a						0.85	
NPAS4	NM_178864	miR-29a						0.75	
NPL	NM_001200050	miR-125b	0.93						
NR1D2	NM_001145425	miR-142-3p			0.58				
NR2F6	NM_005234	miR-142-3p			0.78				
NR3C1	NM_000176	miR-142-3p, miR-29a			0.77			0.71	
NRAS	NM_002524	miR-29b							0.84
NRG3	NM_001010848	miR-125b, miR-155	0.52			0.55			
NRSN1	NM_080723	miR-29a						0.81	
NRXN1	NM_001135659	miR-125b	0.85						
NSD1	NM_022455	miR-29a						> 0.99	
NSF	NM_006178	miR-142-3p			0.65				
NUCKS1	NM_022731	miR-142-3p, miR-223, miR-29a			0.54		0.52	0.69	
NUDT11	NM_018159	miR-142-3p, miR-29a			0.72			0.9	
NUFIP2	NM_020772	miR-29a						0.98	
NUP160	NM_015231	miR-29a						0.88	
OLFML2A	NM_182487	miR-125b	0.99						
OLFML3	NM_020190	miR-155				0.55			
ONECUT2	NM_004852	miR-125b, miR-29a	0.54					0.83	
OSBP	NM_002556	miR-29a						0.6	
OSBPL2	NM_014835	miR-155				0.55			
OSBPL3	NM_015550	miR-29a						0.73	
OSBPL6	NM_001201480	miR-29a						0.83	
OSTC	NM_021227	miR-29a						0.63	
OTUB2	NM_023112	miR-29a						0.83	
OTUD1	NM_001145373	miR-29a						0.75	
OXTR	NM_000916	miR-29a						0.63	
PAFAH1B1	NM_000430	miR-125b	0.61						
PAFAH1B2	NM_002572	miR-142-3p			0.77				
PAG1	NM_018440	miR-29b							0.97
PAIP2	NM_001033112	miR-29b							0.58
PALM	NM_001040134	miR-29a						0.9	

Gene Symbol	Reference Sequence	miRNA(s) Predicted to Target the Gene	miR-125b P_{CT}	miR-126 P_{CT}	miR-142-3p P_{CT}	miR-155 P_{CT}	miR-223 P_{CT}	miR-29a P_{CT}	miR-29b P_{CT}
PALM2	NM_001037293	miR-155, miR-29a				0.52		0.83	
PALM2-AKAP2	NM_007203	miR-29a						0.52	
PAN2	NM_001127460	miR-29a						0.91	
PANK1	NM_138316	miR-155				0.58			
PAPD7	NM_001171805	miR-142-3p			0.55				
PAPOLG	NM_022894	miR-29a						0.83	
PAQR3	NM_001040202	miR-29a						0.72	
PARD6B	NM_032521	miR-142-3p			0.81				
PARG	NM_003631	miR-29a						0.58	
PARP8	NM_001178055	miR-142-3p			0.74				
PATZ1	NM_014323	miR-142-3p			0.62				
PCDH7	NM_001173523	miR-126		0.55					
PCDHA1	NM_018900	miR-29a						0.61	
PCDHA10	NM_018901	miR-29a						0.61	
PCDHA11	NM_018902	miR-29a						0.61	
PCDHA12	NM_018903	miR-29a						0.61	
PCDHA13	NM_018904	miR-29a						0.61	
PCDHA2	NM_018905	miR-29a						0.61	
PCDHA3	NM_018906	miR-29a						0.61	
PCDHA4	NM_018907	miR-29a						0.61	
PCDHA5	NM_018908	miR-29a						0.61	
PCDHA6	NM_018909	miR-29a						0.61	
PCDHA7	NM_018910	miR-29a						0.61	
PCDHA8	NM_018911	miR-29a						0.61	
PCDHA9	NM_031857	miR-29a						0.61	
PCDHAC1	NM_018898	miR-29a						0.61	
PCDHAC2	NM_018899	miR-29a						0.61	
PCGF3	NM_006315	miR-142-3p, miR-29a			0.86			0.92	
PCYT1B	NM_001163264	miR-29a						0.99	
PDE4D	NM_001104631	miR-223					0.52		
PDE8A	NM_002605	miR-142-3p			0.82				
PDGFB	NM_002608	miR-29a						0.83	
PDGFC	NM_016205	miR-29a						0.66	
PDGFRB	NM_002609	miR-29a						0.81	
PDHX	NM_001135024	miR-29a						0.83	
PDIK1L	NM_152835	miR-29a						0.83	
PDK3	NM_001142386	miR-125b	0.87						
PDP2	NM_020786	miR-29a						0.78	
PEAK1	NM_024776	miR-29b							0.93
PER1	NM_002616	miR-29a						0.83	
PER3	NM_016831	miR-29b							0.93
PEX5	NM_000319	miR-29a						0.81	
PGAP1	NM_024989	miR-29a						0.83	
PGAP2	NM_001145438	miR-29a						0.92	
PGRMC2	NM_006320	miR-142-3p			0.75				
PHACTR2	NM_001100164	miR-29a						0.82	
PHC1	NM_004426	miR-29a						0.83	
PHC2	NM_004427	miR-155				0.5			
PHF19	NM_015651	miR-223					0.75		

Gene Symbol	Reference Sequence	miRNA(s) Predicted to Target the Gene	miR-125b P_{CT}	miR-126 P_{CT}	miR-142-3p P_{CT}	miR-155 P_{CT}	miR-223 P_{CT}	miR-29a P_{CT}	miR-29b P_{CT}
PHF21A	NM_001101802	miR-29a						0.85	
PHIP	NM_017934	miR-223					0.6		
PHLDB3	NM_198850	miR-29a						0.69	
PHLPP2	NM_015020	miR-29a						0.84	
PHYHIP	NM_001099335	miR-125b	0.54						
PI15	NM_015886	miR-29b							
PIAS4	NM_015897	miR-29a						0.83	
PICALM	NM_001008660	miR-142-3p			0.65				
PIK3C2B	NM_002646	miR-125b	0.54						
PIK3CD	NM_005026	miR-125b	0.57						
PIK3CG	NM_002649	miR-142-3p			0.7				
PIK3R1	NM_001242466	miR-29a						0.83	
PIK3R3	NM_001114172	miR-29a						0.83	
PIK3R6	NM_001010855	miR-142-3p			0.82				
PIP4K2B	NM_003559	miR-125b	0.9						
PITPNA	NM_006224	miR-29a						0.8	
PITPNM2	NM_020845	miR-29a						0.92	
PITPNM3	NM_001165966	miR-29a						0.79	
PKNOX2	NM_022062	miR-29b							0.78
PL-5283	NM_001130929	miR-29a						0.92	
PLAG1	NM_001114634	miR-29a						0.89	
PLAGL2	NM_002657	miR-223					0.51		
PLCB1	NM_015192	miR-142-3p			0.53				
PLEKHA1	NM_001001974	miR-29b							0.78
PLEKHA8	NM_001197026	miR-125b, miR-29b	0.97						0.94
PLK2	NM_006622	miR-126		0.55					
PLP1	NM_000533	miR-29a						0.82	
PLSCR3	NM_001201576	miR-29a						0.78	
PLXNA1	NM_032242	miR-29a						0.93	
PLXNA4	NM_020911	miR-29a						0.56	
PLXNB2	NM_012401	miR-126		0.55					
PMAIP1	NM_021127	miR-142-3p			0.58				
PMP22	NM_000304	miR-29a						0.79	
POLE3	NM_017443	miR-29a						0.74	
POLR1D	NM_001206559	miR-29a						0.74	
POU2F1	NM_001198783	miR-29a						0.81	
POU2F2	NM_001207025	miR-29a						0.77	
PPARA	NM_001001928	miR-125b, miR-142-3p	0.61		0.56				
PPARD	NM_001171818	miR-29a						0.81	
PPFIA1	NM_003626	miR-142-3p			0.81				
PPIC	NM_000943	miR-29b							0.92
PPIP5K2	NM_015216	miR-29a						0.62	
PPM1A	NM_021003	miR-125b	0.77						
PPM1D	NM_003620	miR-29b							0.83
PPM1E	NM_014906	miR-29a						0.83	
PPM1H	NM_020700	miR-125b	0.59						
PPP1R10	NM_002714	miR-142-3p			0.86				
PPP1R13B	NM_015316	miR-29a						0.83	
PPP1R15B	NM_032833	miR-29a						0.83	

Gene Symbol	Reference Sequence	miRNA(s) Predicted to Target the Gene	miR-125b P_{CT}	miR-126 P_{CT}	miR-142-3p P_{CT}	miR-155 P_{CT}	miR-223 P_{CT}	miR-29a P_{CT}	miR-29b P_{CT}
PPP1R3D	NM_006242	miR-29b							0.9
PPP2CA	NM_002715	miR-125b	0.59						
PPP2R5C	NM_001161725	miR-125b	0.67						
PPP3CA	NM_000944	miR-142-3p			0.66				
PPP3R1	NM_000945	miR-142-3p			0.62				
PPT1	NM_000310	miR-125b	0.68						
PRDM1	NM_001198	miR-125b	0.69						
PRICKLE2	NM_198859	miR-29a						0.83	
PRKAB2	NM_005399	miR-29a						0.82	
PRKG1	NM_001098512	miR-29a						0.77	
PRKRA	NM_001139517	miR-29a						0.83	
PRLR	NM_000949	miR-142-3p			0.62				
PROM1	NM_001145847	miR-142-3p			0.61				
PRR14L	NM_173566	miR-29a						0.99	
PRR3	NM_001077497	miR-29b							0.83
PRRC1	NM_130809	miR-125b	0.73						
PRRC2C	NM_015172	miR-29b							0.81
PRRG1	NM_000950	miR-142-3p			0.61				
PRSS35	NM_001170423	miR-125b	0.83						
PRTG	NM_173814	miR-125b							
PSIP1	NM_021144	miR-142-3p			0.74				
PSMA5	NM_001199772	miR-223					0.5		
PSRC1	NM_001005290	miR-142-3p			0.54				
PTAR1	NM_001099666	miR-125b	0.97						
PTEN	NM_000314	miR-29a						0.84	
PTP4A1	NM_003463	miR-29a						0.63	
PTPN1	NM_002827	miR-29b							0.54
PTPN14	NM_005401	miR-223					0.52		
PTPN2	NM_001207013	miR-155				0.56			
PTPN23	NM_015466	miR-142-3p			0.86				
PTPRD	NM_001040712	miR-29a						0.89	
PTPRJ	NM_002843	miR-155				0.53			
PTRF	NM_012232	miR-29a						0.83	
PTX3	NM_002852	miR-29a						0.69	
PUM1	NM_001020658	miR-142-3p			0.6				
PURA	NM_005859	miR-29a						0.55	
PURB	NM_033224	miR-142-3p, miR-223			0.8		0.59		
PURG	NM_001015508	miR-29a						0.97	
PVRL1	NM_002855	miR-29a						0.68	
PXDN	NM_012293	miR-29a						0.93	
QKI	NM_006775	miR-142-3p			0.85				
RAB11A	NM_001206836	miR-142-3p			0.7				
RAB11FIP2	NM_014904	miR-155				0.5			
RAB14	NM_016322	miR-29b							0.76
RAB15	NM_198686	miR-29a						> 0.99	
RAB1A	NM_004161	miR-142-3p			0.7				
RAB2A	NM_001242644	miR-142-3p			0.83				
RAB30	NM_014488	miR-29a						0.78	
RAB34	NM_001142624	miR-155				0.55			

Gene Symbol	Reference Sequence	miRNA(s) Predicted to Target the Gene	miR-125b P_{CT}	miR-126 P_{CT}	miR-142-3p P_{CT}	miR-155 P_{CT}	miR-223 P_{CT}	miR-29a P_{CT}	miR-29b P_{CT}
RAB3A	NM_002866	miR-142-3p			0.71				
RAB3D	NM_004283	miR-125b, miR-142-3p	0.92		0.58				
RAB40C	NM_001172663	miR-142-3p, miR-29b			0.81				0.77
RAB6B	NM_016577	miR-29a						0.83	
RAB8B	NM_016530	miR-125b	0.86						
RAC1	NM_006908	miR-142-3p			0.72				
RALGPS1	NM_014636	miR-29a						0.94	
RALGPS2	NM_152663	miR-223					0.52		
RAP1B	NM_001010942	miR-155				0.55			
RAP1GAP2	NM_001100398	miR-142-3p			0.83				
RAP1GDS1	NM_001100426	miR-29a						0.83	
RAPGEF5	NM_012294	miR-155				0.55			
RAPGEFL1	NM_016339	miR-29a						0.92	
RAPH1	NM_213589	miR-155, miR-29a				0.6		0.93	
RARB	NM_000965	miR-29b							0.75
RARG	NM_000966	miR-142-3p			0.85				
RASAL2	NM_004841	miR-29a						0.83	
RASGEF1A	NM_145313	miR-125b	0.69						
RASSF3	NM_001242691	miR-125b, miR-142-3p	0.71		0.73				
RBAK	NM_001204456	miR-125b	0.64						
RBBP4	NM_001135255	miR-223					0.51		
RBM20	NM_001134363	miR-125b	0.97						
RBM24	NM_001143941	miR-125b	0.52						
RBM27	NM_018989	miR-142-3p			0.57				
RBM47	NM_001098634	miR-142-3p			0.83				
RBMS1	NM_002897	miR-142-3p			0.66				
RBMS3	NM_001003792	miR-155				0.72			
RCC2	NM_001136204	miR-29a						0.6	
RCOR1	NM_015156	miR-155, miR-29a				0.64		0.82	
RELA	NM_001145138	miR-155				0.53			
REPS2	NM_001080975	miR-29b							0.93
RERE	NM_001042681	miR-142-3p, miR-29b			0.83				0.94
REST	NM_001193508	miR-29a						0.63	
REV3L	NM_002912	miR-29a						0.97	
RFWD3	NM_018124	miR-142-3p			0.62				
RGAG4	NM_001024455	miR-29a						0.64	
RGL2	NM_004761	miR-142-3p			0.82				
RGS3	NM_021106	miR-126		0.56					
RHBDD1	NM_001167608	miR-29a						0.76	
RHEB	NM_005614	miR-142-3p, miR-155			0.83	0.55			
RHOB	NM_004040	miR-223					0.52		
RHOBTB3	NM_014899	miR-142-3p			0.82				
RHOQ	NM_012249	miR-155				0.55			
RIC3	NM_001135109	miR-29a						0.77	
RIC8A	NM_021932	miR-29a						0.83	
RICTOR	NM_152756	miR-142-3p, miR-155				0.64			
RIMKLA	NM_173642	miR-125b	0.74						
RIOK3	NM_003831	miR-29a						0.83	
RIT1	NM_006912	miR-125b, miR-29b	0.76						0.81

Gene Symbol	Reference Sequence	miRNA(s) Predicted to Target the Gene	miR-125b P_{CT}	miR-126 P_{CT}	miR-142-3p P_{CT}	miR-155 P_{CT}	miR-223 P_{CT}	miR-29a P_{CT}	miR-29b P_{CT}
RLF	NM_012421	miR-142-3p, miR-29a			0.8			0.9	
RLIM	NM_016120	miR-155, miR-29a				0.55		0.83	
RMND5A	NM_022780	miR-125b	0.74						
RNF103-VPS24	NM_001198954	miR-142-3p			0.73				
RNF122	NM_024787	miR-29b							0.88
RNF123	NM_022064	miR-155				0.61			
RNF138	NM_001191324	miR-29a						0.81	
RNF141	NM_016422	miR-29a						0.75	
RNF144B	NM_182757	miR-29a						0.79	
RNF157	NM_052916	miR-142-3p			0.73				
RNF165	NM_152470	miR-126, miR-29b		0.56					0.97
RNF169	NM_001098638	miR-29b							0.97
RNF19A	NM_015435	miR-29a						0.81	
RNF214	NM_001077239	miR-29a						0.78	
RNF24	NM_001134337	miR-142-3p			0.7				
RNF39	NM_025236	miR-29a						0.82	
ROBO1	NM_001145845	miR-29b							0.82
ROCK2	NM_004850	miR-142-3p			0.61				
ROD1	NM_001163788	miR-29a						0.72	
RORA	NM_002943	miR-29b							
RPRD1A	NM_018170	miR-142-3p			0.81				
RPS6KA1	NM_001006665	miR-125b	0.76						
RPS6KA3	NM_004586	miR-29a						0.76	
RPS6KA6	NM_014496	miR-155				0.56			
RREB1	NM_001003698	miR-142-3p, miR-155			0.77	0.69			
RSPO2	NM_178565	miR-155				0.55			
RUNX1T1	NM_001198625	miR-29a						0.82	
RYBP	NM_012234	miR-29a						0.82	
S100PBP	NM_022753	miR-155				0.53			
S1PR3	NM_005226	miR-142-3p			0.75				
SALL1	NM_001127892	miR-155				0.53			
SAMD4A	NM_001161576	miR-142-3p, miR-29a			0.59			0.83	
SAP30L	NM_001131062	miR-29a						0.87	
SCAF11	NM_004719	miR-29a						0.72	
SCAI	NM_001144877	miR-29a						0.83	
SCAMP5	NM_001178111	miR-29a						0.94	
SCHIP1	NM_001197107	miR-29b							0.88
SCMH1	NM_001031694	miR-29a						0.75	
SCML2	NM_006089	miR-29b							0.77
SCN3A	NM_001081676	miR-223					0.58		
SCN3B	NM_001040151	miR-29a						0.83	
SCN4B	NM_001142348	miR-125b	0.85						
SCN7A	NM_002976	miR-29a						0.8	
SDC4	NM_002999	miR-142-3p			0.67				
SDK1	NM_152744	miR-29a						0.93	
SEC14L5	NM_014692	miR-155				0.5			
SEL1L	NM_005065	miR-125b	0.7						
SEMA4B	NM_020210	miR-125b	0.95						
SEMA5B	NM_001031702	miR-29a						0.67	

Gene Symbol	Reference Sequence	miRNA(s) Predicted to Target the Gene	miR-125b P_{CT}	miR-126 P_{CT}	miR-142-3p P_{CT}	miR-155 P_{CT}	miR-223 P_{CT}	miR-29a P_{CT}	miR-29b P_{CT}
SENP1	NM_014554	miR-29a						0.83	
SEPT3	NM_019106	miR-29a						0.88	
SEPT6	NM_015129	miR-223					0.51		
SERBP1	NM_001018067	miR-29a						0.92	
SERINC5	NM_001174072	miR-29a						0.83	
SERPINH1	NM_001207014	miR-29b							0.94
SERTAD2	NM_014755	miR-155				0.57			
SESTD1	NM_178123	miR-125b, miR-29a	0.93					> 0.99	
SETBP1	NM_015559	miR-142-3p, miR-223			0.65		0.66		
SETD1B	NM_015048	miR-142-3p			0.84				
SETD7	NM_030648	miR-155				0.55			
SETD8	NM_020382	miR-29a						0.92	
SETDB1	NM_001145415	miR-29a						0.94	
SETDB2	NM_001160308	miR-29b							0.99
SFTA3	NM_001101341	miR-29a						0.71	
SGCB	NM_000232	miR-29a						0.69	
SGK1	NM_001143676	miR-29a						0.74	
SGK494	NM_001174103	miR-29a						0.83	
SGMS2	NM_001136257	miR-29a						0.94	
SGPL1	NM_003901	miR-125b	0.99						
SH2B1	NM_001145795	miR-142-3p			0.62				
SH2B3	NM_005475	miR-125b	0.62						
SH3BP4	NM_014521	miR-125b	0.92						
SH3BP5L	NM_030645	miR-29a						0.98	
SH3GLB1	NM_001206651	miR-142-3p, miR-29a			0.82			0.83	
SH3PXD2A	NM_014631	miR-155, miR-29b				0.55			0.99
SH3RF3	NM_001099289	miR-29a						0.84	
SH3TC2	NM_024577	miR-125b	0.99						
SHOX2	NM_001163678	miR-223					0.52		
SHPRH	NM_001042683	miR-29a						0.93	
SHROOM2	NM_001649	miR-29b							0.83
SHROOM4	NM_020717	miR-29b							0.94
SIAH3	NM_198849	miR-29a						0.66	
SIDT1	NM_017699	miR-29a						0.81	
SIDT2	NM_001040455	miR-29a						0.89	
SIK1	NM_173354	miR-142-3p			0.97				
SIK2	NM_015191	miR-142-3p			0.83				
SIKE1	NM_001102396	miR-29a						0.92	
SIM2	NM_005069	miR-155				0.52			
SIX5	NM_175875	miR-29a						0.81	
SKI	NM_003036	miR-29a						0.83	
SLC16A1	NM_001166496	miR-29a						0.83	
SLC16A14	NM_152527	miR-29a						0.9	
SLC16A2	NM_006517	miR-29b							0.91
SLC16A6	NM_001174166	miR-125b	0.91						
SLC17A5	NM_012434	miR-142-3p			0.77				
SLC17A7	NM_020309	miR-125b	0.8						
SLC1A2	NM_001195728	miR-29a						0.78	
SLC1A3	NM_001166695	miR-142-3p			0.63				

Gene Symbol	Reference Sequence	miRNA(s) Predicted to Target the Gene	miR-125b P_{CT}	miR-126 P_{CT}	miR-142-3p P_{CT}	miR-155 P_{CT}	miR-223 P_{CT}	miR-29a P_{CT}	miR-29b P_{CT}
SLC23A2	NM_005116	miR-223					0.63		
SLC24A4	NM_153646	miR-29b							0.93
SLC25A15	NM_014252	miR-125b, miR-29a	0.91					0.7	
SLC25A22	NM_001191060	miR-142-3p, miR-29b			0.81				0.8
SLC25A44	NM_014655	miR-142-3p, miR-29a			0.73			0.93	
SLC26A6	NM_001040454	miR-125b	0.89						
SLC2A12	NM_145176	miR-155				0.62			
SLC30A3	NM_003459	miR-29a						0.96	
SLC30A7	NM_133496	miR-142-3p			0.73				
SLC31A1	NM_001859	miR-29a						0.97	
SLC35B2	NM_178148	miR-29a						0.76	
SLC35F5	NM_025181	miR-142-3p			0.84				
SLC36A1	NM_078483	miR-29a						0.76	
SLC37A3	NM_032295	miR-142-3p, miR-223			0.86		0.68		
SLC39A1	NM_014437	miR-223					0.51		
SLC39A10	NM_001127257	miR-142-3p			0.72				
SLC39A9	NM_018375	miR-29a						0.82	
SLC43A1	NM_001198810	miR-29a						0.5	
SLC43A2	NM_152346	miR-29a						0.93	
SLC5A8	NM_145913	miR-29a						0.78	
SLC7A11	NM_014331	miR-142-3p			0.56				
SLC7A5	NM_003486	miR-126		0.56					
SLC7A6	NM_001076785	miR-29a						0.98	
SLC8A1	NM_001112800	miR-223					0.57		
SLCO4C1	NM_180991	miR-142-3p			0.82				
SLITRK4	NM_001184749	miR-29a						0.8	
SMAD2	NM_001003652	miR-125b, miR-155	0.72			0.84			
SMAD4	NM_005359	miR-125b	0.58						
SMARCA4	NM_001128844	miR-155				0.55			
SMARCD2	NM_001098426	miR-125b	0.92						
SMCR8	NM_144775	miR-125b	0.89						
SMEK2	NM_001122964	miR-29a						0.78	
SMG1	NM_015092	miR-142-3p			0.8				
SMPD3	NM_018667	miR-29a						0.86	
SMTNL2	NM_001114974	miR-29a						0.86	
SMUG1	NM_014311	miR-142-3p			0.73				
SNIP1	NM_024700	miR-29a						0.6	
SNRK	NM_001100594	miR-29a						0.83	
SNTB2	NM_006750	miR-29a						0.98	
SNX18	NM_001102575	miR-142-3p			0.83				
SNX24	NM_014035	miR-29a						0.81	
SNX4	NM_003794	miR-29a						0.68	
SOCS1	NM_003745	miR-155				0.52			
SOCS6	NM_004232	miR-142-3p, miR-155			0.81	0.55			
SOCS7	NM_014598	miR-29b							0.97
SOS1	NM_005633	miR-155				0.55			
SOX1	NM_005986	miR-155				0.64			
SOX10	NM_006941	miR-155				0.55			
SOX11	NM_003108	miR-125b, miR-142-3p	0.68		0.77				

Gene Symbol	Reference Sequence	miRNA(s) Predicted to Target the Gene	miR-125b P_{CT}	miR-126 P_{CT}	miR-142-3p P_{CT}	miR-155 P_{CT}	miR-223 P_{CT}	miR-29a P_{CT}	miR-29b P_{CT}
SOX12	NM_006943	miR-29b							0.83
SOX6	NM_001145811	miR-223					0.56		
SP1	NM_003109	miR-155, miR-29b				0.57			0.9
SPARC	NM_003118	miR-29a						> 0.99	
SPAST	NM_014946	miR-29a						0.83	
SPATA2L	NM_152339	miR-29a						0.82	
SPEG	NM_001173476	miR-125b	0.87						
SPNS1	NM_001142448	miR-142-3p, miR-29a			0.78			0.94	
SPPL2B	NM_001077238	miR-29a						0.84	
SPRED1	NM_152594	miR-126, miR-142-3p, miR-155		0.56	0.82	0.61			
SPRY1	NM_005841	miR-29a						0.83	
SPRY3	NM_005840	miR-223					0.62		
SPRY4	NM_001127496	miR-29a						0.93	
SPSB4	NM_080862	miR-29a						0.92	
SPTLC2	NM_004863	miR-223, miR-29a					0.52	0.83	
SPTY2D1	NM_194285	miR-155, miR-29a				0.55		0.83	
SRCRB4D	NM_080744	miR-29a						0.82	
SRSF6	NM_006275	miR-125b	0.53						
SS18L1	NM_198935	miR-29b							0.85
ST8SIA3	NM_015879	miR-223					0.54		
ST8SIA4	NM_005668	miR-125b	0.97						
STAG2	NM_001042749	miR-29a						0.81	
STAM	NM_003473	miR-142-3p			0.87				
STAM2	NM_005843	miR-142-3p			0.56				
STAMBP	NM_006463	miR-29a						0.78	
STARD9	NM_020759	miR-29b							0.76
STAT3	NM_003150	miR-125b, miR-29a	0.94					0.71	
STK35	NM_080836	miR-29a						0.65	
STK38L	NM_015000	miR-29b							0.76
STMN2	NM_001199214	miR-29a						0.8	
STMN3	NM_015894	miR-125b	0.56						
STRN	NM_003162	miR-29a						0.97	
STRN3	NM_001083893	miR-142-3p, miR-29a			0.77			0.7	
STRN4	NM_001039877	miR-29a						0.89	
STX12	NM_177424	miR-142-3p			0.73				
STX16	NM_001001433	miR-29a						0.8	
STX17	NM_017919	miR-29a						0.96	
STX1A	NM_001165903	miR-29a						0.83	
STX6	NM_005819	miR-125b	0.65						
STX7	NM_003569	miR-142-3p			0.69				
STXBP5L	NM_014980	miR-155				0.58			
SUB1	NM_006713	miR-29a						0.64	
SUFU	NM_016169	miR-155				0.55			
SUN1	NM_001130965	miR-125b, miR-29a	0.72					0.83	
SUV420H1	NM_017635	miR-29a						0.78	
SUV420H2	NM_032701	miR-29a						0.99	
SVEP1	NM_153366	miR-29a						0.81	
SVIL	NM_003174	miR-29a						0.92	
SYNCRIP	NM_001159673	miR-29a						0.75	

Gene Symbol	Reference Sequence	miRNA(s) Predicted to Target the Gene	miR-125b P_{CT}	miR-126 P_{CT}	miR-142-3p P_{CT}	miR-155 P_{CT}	miR-223 P_{CT}	miR-29a P_{CT}	miR-29b P_{CT}
SYNRG	NM_001163544	miR-223					0.55		
SYPL1	NM_006754	miR-142-3p			0.8				
SYPL2	NM_001040709	miR-29a						0.97	
SYT14	NM_001146261	miR-29a						0.65	
SYT2	NM_001136504	miR-29a						0.82	
SYT7	NM_004200	miR-29a						0.94	
SYT9	NM_175733	miR-29a						0.89	
SYVN1	NM_032431	miR-155				0.55			
TAB2	NM_015093	miR-142-3p			0.79				
TACC1	NM_001122824	miR-29a						0.83	
TACC2	NM_006997	miR-125b	0.51						
TAF11	NM_005643	miR-29b							0.75
TAGAP	NM_054114	miR-142-3p			0.75				
TANC1	NM_001145909	miR-29a						0.94	
TAOK1	NM_020791	miR-125b, miR-142-3p	0.61		0.9				
TARDBP	NM_007375	miR-142-3p			0.67				
TATDN2	NM_014760	miR-29a						0.93	
TBC1D1	NM_015173	miR-125b	0.97						
TBC1D15	NM_001146213	miR-155, miR-223				0.57	0.5		
TBC1D2B	NM_015079	miR-142-3p			0.77				
TBC1D7	NM_001143964	miR-29a						0.83	
TBC1D8B	NM_017752	miR-125b	0.62						
TBL1X	NM_001139466	miR-142-3p			0.87				
TBX21	NM_013351	miR-29a						0.83	
TBX4	NM_018488	miR-125b	0.76						
TCEB3	NM_003198	miR-142-3p			0.83				
TCF4	NM_001083962	miR-155				0.53			
TDG	NM_003211	miR-29a						0.82	
TEAD1	NM_021961	miR-142-3p			0.82				
TESK2	NM_007170	miR-142-3p			0.67				
TET1	NM_030625	miR-29a						> 0.99	
TET2	NM_001127208	miR-29a						> 0.99	
TET3	NM_144993	miR-142-3p, miR-29a			0.76			> 0.99	
TEX2	NM_018469	miR-142-3p			0.5				
TFAP2C	NM_003222	miR-29b							0.87
TFDP2	NM_001178138	miR-155				0.55			
TFEB	NM_001167827	miR-29a						0.91	
TFEC	NM_001018058	miR-29a						0.92	
TFG	NM_001007565	miR-142-3p			0.72				
TGFB2	NM_001135599	miR-142-3p, miR-29a			0.71			0.83	
TGFBR1	NM_001130916	miR-142-3p			0.87				
TGIF2	NM_001199513	miR-29a						0.77	
THAP6	NM_144721	miR-126		0.54					
TIAM1	NM_003253	miR-29b							0.64
TIMM17B	NM_001167947	miR-125b	0.7						
TIPARP	NM_001184717	miR-142-3p			0.81				
TIRAP	NM_001039661	miR-142-3p			0.81				
TLE4	NM_007005	miR-155				0.56			
TLL1	NM_012464	miR-29a						0.93	

Gene Symbol	Reference Sequence	miRNA(s) Predicted to Target the Gene	miR-125b P_{CT}	miR-126 P_{CT}	miR-142-3p P_{CT}	miR-155 P_{CT}	miR-223 P_{CT}	miR-29a P_{CT}	miR-29b P_{CT}
TMCC2	NM_001242925	miR-125b	0.68						
TMED9	NM_017510	miR-125b	0.63						
TMEM110	NM_198563	miR-142-3p			0.77				
TMEM115	NM_007024	miR-142-3p			0.77				
TMEM127	NM_001193304	miR-29a						0.83	
TMEM132A	NM_017870	miR-29a						0.58	
TMEM135	NM_001168724	miR-125b	0.94						
TMEM136	NM_001198670	miR-155				0.55			
TMEM151B	NM_001137560	miR-29a						0.83	
TMEM161B	NM_153354	miR-125b	0.65						
TMEM168	NM_022484	miR-125b	0.91						
TMEM169	NM_001142310	miR-29b							0.97
TMEM170B	NM_001100829	miR-125b	0.69						
TMEM180	NM_024789	miR-125b	0.9						
TMEM183A	NM_138391	miR-29a						0.83	
TMEM183B	NM_001079809	miR-29a						0.83	
TMEM200B	NM_001003682	miR-142-3p			0.81				
TMEM201	NM_001130924	miR-29a						0.82	
TMEM229B	NM_182526	miR-29a						0.83	
TMEM236	NM_001098844	miR-29a						0.89	
TMEM33	NM_018126	miR-29a						0.8	
TMEM64	NM_001008495	miR-223					0.5		
TMEM65	NM_194291	miR-29a						0.97	
TMEM86A	NM_153347	miR-29a						0.83	
TMOD3	NM_014547	miR-29a						0.83	
TMPRSS13	NM_001077263	miR-125b	0.95						
TMTC3	NM_181783	miR-29a						0.83	
TNFAIP1	NM_021137	miR-29a						0.78	
TNFAIP3	NM_006290	miR-29a						0.94	
TNFRSF1A	NM_001065	miR-29a						0.82	
TNKS	NM_003747	miR-142-3p			0.83				
TNRC18	NM_001080495	miR-142-3p, miR-29a			0.82			0.83	
TNRC6B	NM_001024843	miR-29a						0.94	
TOM1	NM_001135729	miR-126		0.54					
TOMM20	NM_014765	miR-155				0.59			
TOR2A	NM_001085347	miR-125b	0.87						
TP53INP1	NM_001135733	miR-155, miR-29a				0.64		0.89	
TP53INP2	NM_021202	miR-142-3p, miR-29b			0.8				0.81
TPK1	NM_001042482	miR-29a						0.61	
TPM4	NM_001145160	miR-142-3p			0.83				
TRA2B	NM_004593	miR-29a						0.83	
TRAF3	NM_001199427	miR-29a						> 0.99	
TRAF4	NM_004295	miR-29a						0.9	
TRAF5	NM_001033910	miR-29a						0.61	
TRAF6	NM_004620	miR-125b	0.77						
TRAF7	NM_032271	miR-126		0.56					
TRAFD1	NM_001143906	miR-29b							0.66
TRAM2	NM_012288	miR-29a						0.67	
TRIAP1	NM_016399	miR-125b	0.82						

Gene Symbol	Reference Sequence	miRNA(s) Predicted to Target the Gene	miR-125b P_{CT}	miR-126 P_{CT}	miR-142-3p P_{CT}	miR-155 P_{CT}	miR-223 P_{CT}	miR-29a P_{CT}	miR-29b P_{CT}
TRIB2	NM_021643	miR-125b, miR-29a	0.54					0.99	
TRIL	NM_014817	miR-29b							0.85
TRIM32	NM_001099679	miR-155				0.54			
TRIM37	NM_015294	miR-29a						0.65	
TRIM44	NM_017583	miR-155				0.62			
TRIM63	NM_032588	miR-29a						0.83	
TRIM66	NM_014818	miR-29a						0.73	
TRIM9	NM_015163	miR-29a						0.94	
TRPC6	NM_004621	miR-29a						0.53	
TRPM8	NM_024080	miR-29b							0.85
TRPS1	NM_014112	miR-155				0.82			
TSC22D3	NM_001015881	miR-29a						0.59	
TSEN34	NM_001077446	miR-142-3p			0.59				
TSHZ3	NM_020856	miR-155, miR-223				0.81	0.5		
TSPAN14	NM_001128309	miR-29a						0.98	
TSPAN4	NM_001025234	miR-29a						0.75	
TSPAN9	NM_001168320	miR-29a						0.94	
TSTA3	NM_003313	miR-125b	0.86						
TTC14	NM_001042601	miR-29b							0.83
TTC30B	NM_152517	miR-29a						0.69	
TTC9	NM_015351	miR-29a						0.85	
TTPA	NM_000370	miR-125b	0.67						
TTYH2	NM_032646	miR-29a						0.83	
TUBB2A	NM_001069	miR-29a						0.57	
TUBB2B	NM_178012	miR-29a						0.83	
TWF1	NM_001242397	miR-142-3p			0.73				
TXLNG	NM_001168683	miR-29a						0.68	
U2SURP	NM_001080415	miR-29b							0.83
UACA	NM_001008224	miR-29a						0.74	
UBASH3B	NM_032873	miR-125b	0.71						
UBE2G1	NM_003342	miR-125b	0.72						
UBE2H	NM_001202498	miR-29a						0.86	
UBE2K	NM_001111112	miR-29a						0.83	
UBE2R2	NM_017811	miR-125b							
UBE2W	NM_001001481	miR-223					0.51		
UBFD1	NM_019116	miR-29a						0.8	
UBIAD1	NM_013319	miR-29a						0.67	
UBN2	NM_173569	miR-29a						0.81	
UBR7	NM_175748	miR-125b	0.71						
UBTD1	NM_024954	miR-125b	0.6						
UBTD2	NM_152277	miR-29a						0.82	
UBTF	NM_001076683	miR-29b							0.95
UHMK1	NM_001184763	miR-223					0.52		
UHRF1BP1	NM_017754	miR-29a						0.89	
ULK2	NM_014683	miR-155				0.55			
UNC13B	NM_006377	miR-29a						0.74	
UNK	NM_001080419	miR-29a						0.72	
UNKL	NM_001193388	miR-142-3p, miR-29b			0.62				0.71
UPK1B	NM_006952	miR-29a						0.74	

Gene Symbol	Reference Sequence	miRNA(s) Predicted to Target the Gene	miR-125b P_{CT}	miR-126 P_{CT}	miR-142-3p P_{CT}	miR-155 P_{CT}	miR-223 P_{CT}	miR-29a P_{CT}	miR-29b P_{CT}
UPP2	NM_001135098	miR-155				0.54			
UQCC	NM_001184977	miR-223					0.51		
UQCR11	NM_006830	miR-155				0.52			
URM1	NM_001135947	miR-29a						0.86	
USP2	NM_004205	miR-125b, miR-29a	0.94					0.82	
USP31	NM_020718	miR-29a						0.94	
USP34	NM_014709	miR-29a						0.8	
USP37	NM_020935	miR-29a						0.83	
USP42	NM_032172	miR-29a						0.79	
USP48	NM_032236	miR-155				0.57			
USP6NL	NM_001080491	miR-142-3p, miR-29a			0.88			0.82	
UST	NM_005715	miR-155				0.55			
UTRN	NM_007124	miR-142-3p			0.73				
UTY	NM_007125	miR-142-3p			0.83				
UVRAG	NM_003369	miR-125b	0.77						
VAMP3	NM_004781	miR-142-3p			0.82				
VAMP7	NM_001145149	miR-29a						0.77	
VANGL1	NM_001172411	miR-29a						0.83	
VANGL2	NM_020335	miR-125b	0.62						
VASH1	NM_014909	miR-29a						0.88	
VASH2	NM_001136474	miR-29a						0.83	
VAV3	NM_001079874	miR-125b	0.53						
VCL	NM_003373	miR-29b							0.83
VCPIP1	NM_025054	miR-125b, miR-223	0.93				0.58		
VEGFA	NM_001025366	miR-29a						0.92	
VHL	NM_000551	miR-29a						0.75	
VIPAR	NM_001193314	miR-29b							0.61
VPS18	NM_020857	miR-155				0.54			
VPS24	NM_001005753	miR-142-3p			0.73				
VPS25	NM_032353	miR-29a						0.74	
VPS36	NM_016075	miR-29a						0.8	
VPS37C	NM_017966	miR-29b							0.83
VTA1	NM_016485	miR-29a						0.8	
VTI1A	NM_145206	miR-155				0.55			
WAC	NM_016628	miR-29b							0.74
WASF1	NM_001024934	miR-29a						0.73	
WASF2	NM_001201404	miR-29a						0.91	
WASL	NM_003941	miR-142-3p			0.91				
WDFY1	NM_020830	miR-29a						0.93	
WDR26	NM_001115113	miR-29a						0.54	
WDR81	NM_001163673	miR-29a						0.63	
WEE1	NM_001143976	miR-155				0.55			
WIPF2	NM_133264	miR-142-3p			0.57				
WISP1	NM_001204869	miR-29b							0.82
WIZ	NM_021241	miR-142-3p			0.87				
WSCD2	NM_014653	miR-29a						0.6	
WWTR1	NM_001168278	miR-29a						0.84	
XIAP	NM_001167	miR-142-3p			0.63				
XKR4	NM_052898	miR-29a						0.79	

Gene Symbol	Reference Sequence	miRNA(s) Predicted to Target the Gene	miR-125b P_{CT}	miR-126 P_{CT}	miR-142-3p P_{CT}	miR-155 P_{CT}	miR-223 P_{CT}	miR-29a P_{CT}	miR-29b P_{CT}
XKR6	NM_173683	miR-29b							0.97
XKR7	NM_001011718	miR-29a						0.92	
XPNPEP1	NM_001167604	miR-29a						0.92	
XPO1	NM_003400	miR-142-3p			0.82				
XPO4	NM_022459	miR-29a						0.69	
XRN1	NM_001042604	miR-29a						0.78	
YPEL2	NM_001005404	miR-29a						0.97	
YRDC	NM_024640	miR-29a						0.7	
YWHAE	NM_006761	miR-29b							0.53
YY1	NM_003403	miR-29a						0.81	
ZBTB10	NM_001105539	miR-142-3p, miR-29a			0.81			0.59	
ZBTB20	NM_001164342	miR-142-3p			0.65				
ZBTB34	NM_001099270	miR-29a						> 0.99	
ZBTB37	NM_001122770	miR-125b	0.53						
ZBTB38	NM_001080412	miR-125b	0.6						
ZBTB40	NM_001083621	miR-29b							0.83
ZBTB41	NM_194314	miR-142-3p, miR-29a			0.82			0.73	
ZBTB43	NM_001135776	miR-29a						0.96	
ZBTB46	NM_025224	miR-29a						0.97	
ZBTB47	NM_145166	miR-29b							0.99
ZBTB5	NM_014872	miR-29b							0.96
ZBTB7A	NM_015898	miR-125b	0.94						
ZC4H2	NM_001178032	miR-29a						0.91	
ZCCHC14	NM_015144	miR-142-3p, miR-223			0.95		0.57		
ZCCHC17	NM_016505	miR-29b							0.76
ZCCHC24	NM_153367	miR-142-3p			0.74				
ZDHHC15	NM_001146256	miR-29b							0.73
ZDHHC21	NM_178566	miR-29a						0.83	
ZDHHC5	NM_015457	miR-29a						0.63	
ZEB1	NM_001128128	miR-142-3p			0.7				
ZEB2	NM_001171653	miR-142-3p			0.88				
ZER1	NM_006336	miR-29a						0.81	
ZFHX3	NM_001164766	miR-223					0.51		
ZFP36	NM_003407	miR-29b							0.83
ZFP36L1	NM_004926	miR-29b							0.81
ZFP62	NM_001172638	miR-125b	0.91						
ZFP91	NM_001197051	miR-142-3p, miR-29b			0.74				0.83
ZFX	NM_001178084	miR-29a						0.66	
ZFYVE1	NM_021260	miR-125b	0.94						
ZFYVE20	NM_022340	miR-142-3p, miR-29a			0.75			0.76	
ZFYVE26	NM_015346	miR-29a						0.81	
ZHX3	NM_015035	miR-29b							0.92
ZIC3	NM_003413	miR-155				0.64			
ZIC5	NM_033132	miR-29b							0.61
ZMIZ1	NM_020338	miR-29a						0.94	
ZMYM2	NM_001190964	miR-155, miR-29b				0.59			0.82
ZMYM3	NM_001171162	miR-29a						0.58	
ZMYND8	NM_012408	miR-142-3p			0.83				
ZNF12	NM_006956	miR-125b	0.65						

Gene Symbol	Reference Sequence	miRNA(s) Predicted to Target the Gene	miR-125b P_{CT}	miR-126 P_{CT}	miR-142-3p P_{CT}	miR-155 P_{CT}	miR-223 P_{CT}	miR-29a P_{CT}	miR-29b P_{CT}
ZNF167	NM_018651	miR-29a						0.74	
ZNF217	NM_006526	miR-142-3p			0.87				
ZNF219	NM_001101672	miR-126		0.54					
ZNF281	NM_012482	miR-125b	0.53						
ZNF282	NM_003575	miR-29a						> 0.99	
ZNF304	NM_020657	miR-125b	0.54						
ZNF346	NM_012279	miR-29a						> 0.99	
ZNF362	NM_152493	miR-29a						0.83	
ZNF385C	NM_001242704	miR-29a						0.78	
ZNF395	NM_018660	miR-142-3p			0.81				
ZNF469	NM_001127464	miR-29a						0.94	
ZNF512B	NM_020713	miR-29a						0.94	
ZNF518B	NM_053042	miR-29a						0.83	
ZNF532	NM_018181	miR-29a						0.87	
ZNF609	NM_015042	miR-29b							0.74
ZNF618	NM_133374	miR-142-3p, miR-155			0.82	0.66			
ZNF644	NM_016620	miR-155				0.55			
ZNF652	NM_001145365	miR-142-3p, miR-155			0.72	0.86			
ZNF703	NM_025069	miR-155				0.55			
ZNF704	NM_001033723	miR-29a						0.84	
ZNF827	NM_178835	miR-142-3p			0.76				
ZNF831	NM_178457	miR-142-3p			0.8				
ZNRF3	NM_001206998	miR-155				0.55			
ZSWIM4	NM_023072	miR-125b	0.73						
ZSWIM5	NM_020883	miR-125b	0.97						
ZXDC	NM_001040653	miR-223					0.52		
ZZZ3	NM_015534	miR-142-3p			0.59				

Table S4. The miRNA-mediated AML gene signature, consisting of 78 genes.

Gene Symbol	Reference Sequence	Gene Name	miRNA(s) Predicted to Target the Gene
ADAM19	NM_033274	ADAM metallopeptidase domain 19	miR-29a
AHR	NM_001621	aryl hydrocarbon receptor	miR-29a
ASPH	NM_001164756	aspartate beta-hydroxylase	miR-29a
ATP1B1	NM_001677	ATPase, Na+/K+ transporting, beta 1 polypeptide	miR-29a
BACH2	NM_001170794	BTB and CNC homology 1, basic leucine zipper transcription factor 2	miR-142-3p, miR-29a
BCL11B	NM_022898	B-cell CLL/lymphoma 11B (zinc finger protein)	miR-29b
BMP1	NM_001199	bone morphogenetic protein 1	miR-29a
C11orf57	NM_001082969	chromosome 11 open reading frame 57	miR-29a
CBX2	NM_005189	chromobox homolog 2	miR-29a
CD8A	NM_001145873	CD8a molecule	miR-142-3p
CDK6	NM_001145306	cyclin-dependent kinase 6	miR-29a
CEACAM1	NM_001024912	carcinoembryonic antigen-related cell adhesion molecule 1 (biliary glycoprotein)	miR-29a
COL24A1	NM_152890	collagen, type XXIV, alpha 1	miR-142-3p, miR-29a
COL4A5	NM_000495	collagen, type IV, alpha 5	miR-29b
DNMT3B	NM_001207055	DNA (cytosine-5-)-methyltransferase 3 beta	miR-29a
DSC2	NM_004949	desmocollin 2	miR-29a
EGFL7	NM_016215	EGF-like-domain, multiple 7	miR-126
EMP1	NM_001423	epithelial membrane protein 1	miR-29a
ENTPD1	NM_001098175	ectonucleoside triphosphate diphosphohydrolase 1	miR-125b
ETS1	NM_001143820	v-ets erythroblastosis virus E26 oncogene homolog 1 (avian)	miR-155
F3	NM_001178096	coagulation factor III (thromboplastin, tissue factor)	miR-223
FAM176B	NM_018166	family with sequence similarity 176, member B	miR-29a
FBXO41	NM_001080410	F-box protein 41	miR-29a
FGD4	NM_139241	FYVE, RhoGEF and PH domain containing 4	miR-29a
FRAT2	NM_012083	frequently rearranged in advanced T-cell lymphomas 2	miR-29a
HOXB3	NM_002146	homeobox B3	miR-125b
HSPG2	NM_005529	heparan sulfate proteoglycan 2	miR-29a
HTR7	NM_000872	5-hydroxytryptamine (serotonin) receptor 7 (adenylate cyclase-coupled)	miR-29a
IFFO2	NM_001136265	intermediate filament family orphan 2	miR-29a
IL1RAP	NM_001167928	interleukin 1 receptor accessory protein	miR-29a
JHDM1D	NM_030647	jumonji C domain containing histone demethylase 1 homolog D (S. cerevisiae)	miR-155
KAT2B	NM_003884	K(lysine) acetyltransferase 2B	miR-142-3p
KIAA1549	NM_001164665	KIAA1549	miR-29a
LUZP1	NM_001142546	leucine zipper protein 1	miR-29a
MAF	NM_001031804	v-maf musculoaponeurotic fibrosarcoma oncogene homolog (avian)	miR-125b
MAST4	NM_001164664	microtubule associated serine/threonine kinase family member 4	miR-29a
MEGF9	NM_001080497	multiple EGF-like-domains 9	miR-125b
MEST	NM_002402	mesoderm specific transcript homolog (mouse)	miR-29a
MEX3B	NM_032246	mex-3 homolog B (C. elegans)	miR-29a
MGAT4A	NM_012214	mannosyl (alpha-1,3-)-glycoprotein beta-1,4-N-acetylglucosaminyltransferase, isozyme A	miR-125b, miR-142-3p, miR-29b
MLLT11	NM_006818	myeloid/lymphoid or mixed-lineage leukemia (trithorax homolog, Drosophila); translocated to, 11	miR-29a
MMP2	NM_001127891	matrix metallopeptidase 2 (gelatinase A, 72kDa gelatinase, 72kDa type IV collagenase)	miR-29a
MSRB3	NM_001031679	methionine sulfoxide reductase B3	miR-125b
MYCN	NM_005378	v-myc myelocytomatosis viral related oncogene, neuroblastoma	miR-29a

Gene Symbol	Reference Sequence	Gene Name	miRNA(s) Predicted to Target the Gene
		derived (avian)	
MYH10	NM_005964	myosin, heavy chain 10, non-muscle	miR-142-3p, miR-223
NAV2	NM_001111018	neuron navigator 2	miR-29a
NFIA	NM_001134673	nuclear factor I/A	miR-155, miR-223, miR-29a
NKX2-3	NM_145285	NK2 homeobox 3	miR-142-3p
NPL	NM_001200050	N-acetylneuraminate pyruvate lyase (dihydrodipicolinate synthase)	miR-125b
OLFML2A	NM_182487	olfactomedin-like 2A	miR-125b
PAG1	NM_018440	phosphoprotein associated with glycosphingolipid microdomains 1	miR-29b
PALM	NM_001040134	paralemmin	miR-29a
PER1	NM_002616	period homolog 1 (Drosophila)	miR-29a
PHC1	NM_004426	polyhomeotic homolog 1 (Drosophila)	miR-29a
PHC2	NM_004427	polyhomeotic homolog 2 (Drosophila)	miR-155
PLK2	NM_006622	polo-like kinase 2	miR-126
PLXNB2	NM_012401	plexin B2	miR-126
PROM1	NM_001145847	prominin 1	miR-142-3p
PTPRJ	NM_002843	protein tyrosine phosphatase, receptor type, J	miR-155
PXDN	NM_012293	peroxidasin homolog (Drosophila)	miR-29a
RAB34	NM_001142624	RAB34, member RAS oncogene family	miR-155
RNF141	NM_016422	ring finger protein 141	miR-29a
RORA	NM_002943	RAR-related orphan receptor A	miR-29b
SCHIP1	NM_001197107	schwannomin interacting protein 1	miR-29b
SLC16A14	NM_152527	solute carrier family 16, member 14 (monocarboxylic acid transporter 14)	miR-29a
SLC16A6	NM_001174166	solute carrier family 16, member 6 (monocarboxylic acid transporter 7)	miR-125b
SLC37A3	NM_032295	solute carrier family 37 (glycerol-3-phosphate transporter), member 3	miR-142-3p, miR-223
SLCO4C1	NM_180991	solute carrier organic anion transporter family, member 4C1	miR-142-3p
SLITRK4	NM_001184749	SLIT and NTRK-like family, member 4	miR-29a
SNTB2	NM_006750	syntrophin, beta 2 (dystrophin-associated protein A1, 59kDa, basic component 2)	miR-29a
SOCS6	NM_004232	suppressor of cytokine signaling 6	miR-142-3p, miR-155
TANC1	NM_001145909	tetratricopeptide repeat, ankyrin repeat and coiled-coil containing 1	miR-29a
TPM4	NM_001145160	tropomyosin 4	miR-142-3p
WASF1	NM_001024934	WAS protein family, member 1	miR-29a
ZBTB46	NM_025224	zinc finger and BTB domain containing 46	miR-29a
ZNF618	NM_133374	zinc finger protein 618	miR-142-3p, miR-155
ZNF827	NM_178835	zinc finger protein 827	miR-142-3p
ZNF831	NM_178457	zinc finger protein 831	miR-142-3p

Table S5. Networks constructed using the miRNA-mediated AML gene signature.

Network Number	Molecules in Network	*p*-value
1	ADAM19, AHR, Ap1, ATP1B1, BCL11B, Cbp/p300, CBX2, CD3, CDK6, Collagen type IV, Cyclin E, DNMT3B, E2f, ENTPD1, Estrogen Receptor, ETS1, FSH, HISTONE, Histone h4, HOXB3, IL1RAP, KAT2B, MAF, MMP2, NFkB (complex), PAG1, PDGF BB, PHC1, PHC2, PLK2, PROM1, Rb, RORA, Tgf beta, Thymidine Kinase	1E-44
2	ADCY8, ADCY, ARRB1, ASPH, BACH2, Ca2+, CASQ2, CASQ, DSC2, EGFL7, ENTPD1, HNRNPA2B1, HOXA3, HTT, LDHC, LOX, LOXL2, MAST4, MEST, mir-22, miR-223, MLLT11, NAV2, NFIA, NKX2-3, OLFML2A, OSM, PALM, PER1, SLC16A6, SNTB2, TPM4, TRDN, tretinoin, ZNF263	1E-36
3	Akt, BMP1, CEACAM1 (includes others), COL24A1, COL4A5, collagen, Collagen type I, Collagen(s), EMP1, ERK1/2, F3, FGD4, Fibrinogen, hCG, HSPG2, HTR7, IL1, Immunoglobulin, Insulin, Integrin, Jnk, LDL, P38 MAPK, p85 (pik3r), Pdgf (complex), PI3K (complex), Pka, Pkc(s), PLXNB2, PTPRJ, Ras, Ras homolog, SOCS6, Vegf, WASF1	1E-26
4	ATP1B1, beta-estradiol, butyric acid, COLEC12, CYP1, FGFBP1, FMO2, FRAT2, GNE, HMGN2, HUNK, KIAA1549, MEX3B, miR-22, mir-22, mir-103, NPL, PERP, PLK2, progesterone, PROM1, PXDN, RNF141, SCHIP1, SLC37A3, SMARCA4, SOD2, SPOCK1, SULT1E1, TADA2B, TBXAS1, TGFBI, TP53 (includes EG:22059), UBE2D3, ZNF618	1E-23
5	CD8A, CD8B, CENPB, Ck2, CLDN4, ELP2, ENTPD1, ERBB2, ERK, FAU, FGF4, HES6, Histone h3, HMGN2, IL12 (complex), JHDM1D, LUZP1, MED1 (includes EG:19014), MGAT4A, MSRB3, MXD3, MYCN, MYH10, PTMA, PTPRCAP, RAB34, RNA polymerase II, RPL12, RPL13A, RPL35A, RPLP2, RPS25, SRF, TCR, TRPM7	1E-16

Table S6. Biological functions and disease signatures associated with the AML Signalisome.

Function or Disease Signature	*p*-value	Molecules
Cellular Development	0.000001	CEACAM1, BACH2, PHC2, MAF, NKX2-3, PALM, BCL11B, CD8A, DNMT3B, RORA, PLK2, PLXNB2, AHR, BMP1, ETS1, MYCN, MLLT11, ATP1B1, PHC1, CDK6, CBX2, MMP2, KAT2B, HSPG2, PAG1, NFIA
Cancer	0.000003	PER1, CEACAM1, MYH10, MEGF9, MAF, ZNF827, BCL11B, CD8A, PROM1, ZNF831, DNMT3B, HOXB3, PTPRJ, OLFML2A, RORA, PLK2, FGD4, PXDN, SCHIP1, PLXNB2, ASPH, IL1RAP, AHR, BMP1, ETS1, MYCN, ATP1B1, ENTPD1, CDK6, CBX2, MMP2, F3, EGFL7, EMP1, KAT2B, MEST
Hematological System Development and Function	0.000003	ETS1, CEACAM1, MYCN, MLLT11, BACH2, ENTPD1, PHC1, MAF, CDK6, NKX2-3, BCL11B, CD8A, F3, HOXB3, HSPG2, PTPRJ, RORA, PAG1, NFIA, IL1RAP, AHR
Hematopoiesis	0.000003	ETS1, MYCN, MLLT11, BACH2, PHC1, MAF, CDK6, NKX2-3, BCL11B, CD8A, HOXB3, RORA, NFIA, PAG1, AHR
Tissue Development	0.000009	MYH10, CEACAM1, PHC2, MAF, NKX2-3, PALM, BCL11B, HOXB3, PTPRJ, OLFML2A, RORA, ADAM19, PXDN, SCHIP1, DSC2, AHR, BMP1, ETS1, MYCN, ATP1B1, ENTPD1, SLITRK4, PHC1, CBX2, MMP2, F3, EGFL7, COL4A5, EMP1, HSPG2, NFIA, MEST
Cell Cycle	0.000016	ETS1, MYCN, PER1, PHC2, PHC1, CDK6, CBX2, DNMT3B, EMP1, HOXB3, KAT2B, PLK2, AHR
Connective Tissue Development and Function	0.000016	ETS1, MYCN, PHC2, PHC1, CDK6, CBX2, MMP2, F3, DNMT3B, HSPG2, PLK2, SCHIP1, AHR, BMP1
Embryonic Development	0.000016	ETS1, MYCN, MYH10, ATP1B1, PHC2, PHC1, MAF, CBX2, MMP2, NKX2-3, BCL11B, F3, DNMT3B, EMP1, HOXB3, HSPG2, PTPRJ, RORA, ADAM19, SCHIP1, PLXNB2, ASPH, DSC2, AHR
Organismal Development	0.000016	ETS1, CEACAM1, MYH10, MYCN, ATP1B1, PHC2, PHC1, MAF, CBX2, MMP2, NKX2-3, BCL11B, F3, DNMT3B, EMP1, HOXB3, HSPG2, PTPRJ, RORA, ADAM19, SCHIP1, ASPH, DSC2, AHR
Skeletal and Muscular System Development and Function	0.000016	ETS1, MYCN, PHC2, PHC1, CBX2, MMP2, F3, EGFL7, HOXB3, HSPG2, PLK2, SCHIP1, ASPH, DSC2, BMP1
Cell-mediated Immune Response	0.000040	ETS1, MLLT11, RORA, PAG1, MAF, BCL11B, CD8A, AHR
Cellular Function and Maintenance	0.000040	ETS1, CEACAM1, MYH10, MYCN, PER1, MLLT11, BACH2, ENTPD1, MAF, CBX2, NKX2-3, BCL11B, CD8A, F3, EGFL7, RORA, PAG1, FGD4, ASPH, DSC2, AHR
Lymphoid Tissue Structure and Development	0.000040	ETS1, MLLT11, RORA, PAG1, MAF, PHC1, CBX2, NKX2-3, BCL11B, CD8A, AHR
Cellular Growth and Proliferation	0.000072	PER1, MYH10, CEACAM1, BACH2, MAF, SOCS6, PALM, NKX2-3, BCL11B, CD8A, DNMT3B, HOXB3, PTPRJ, RORA, PLK2, PXDN, PLXNB2, ASPH, AHR, BMP1, ETS1, MYCN, MLLT11, ENTPD1, PHC1, CDK6, CBX2, MMP2, F3, EGFL7, EMP1, KAT2B, HSPG2, NFIA, PAG1, MEST
Cell-To-Cell Signaling and Interaction	0.000094	ETS1, CEACAM1, MYCN, PER1, ATP1B1, ENTPD1, PHC1, MAF, MMP2, CD8A, F3, COL4A5, HSPG2, PTPRJ, RORA, PAG1, IL1RAP, AHR
Cellular Movement	0.000094	ETS1, MYCN, MYH10, CEACAM1, PHC2, ENTPD1, WASF1, NKX2-3, MMP2, CD8A, F3, EGFL7, DNMT3B, PTPRJ, NFIA, ADAM19, SCHIP1, PLXNB2, AHR
Inflammatory Response	0.000094	ETS1, CEACAM1, BACH2, ENTPD1, MAF, WASF1, NKX2-3, MMP2, CD8A, F3, HSPG2, PTPRJ, RORA, PAG1, IL1RAP, AHR
Tissue Morphology	0.000094	ETS1, MYH10, ENTPD1, PHC2, PHC1, MAF, MMP2, NKX2-3, CD8A, F3, DNMT3B, HOXB3, HSPG2, NFIA, AHR

References

1. Metzeler, K.H.; Hummel, M.; Bloomfield, C.D.; Spiekermann, K.; Braess, J.; Sauerland, M.C.; Heinecke, A.; Radmacher, M.; Marcucci, G.; Whitman, S.P.; *et al.* An 86-probe-set gene-expression signature predicts survival in cytogenetically normal acute myeloid leukemia. *Blood* **2008**, *112*, 4193–4201.

© 2012 by the authors; licensee MDPI, Basel, Switzerland. This article is an open access article distributed under the terms and conditions of the Creative Commons Attribution license (http://creativecommons.org/licenses/by/3.0/).
